# Adaptation of the Wound Healing Questionnaire universal-reporter outcome measure for use in global surgery trials (TALON-1 study): mixed-methods study and Rasch analysis

**DOI:** 10.1093/bjs/znad058

**Published:** 2023-04-03

**Authors:** James Glasbey, NIHR Global Health Research Unit on Global Surgery, James Glasbey, Adesoji Ademuyiwa, Alisha Bhatt, Bruce Biccard, Jane Blazeby, Peter Brocklehurst, Sohini Chakrabortee, JC Allen Ingabire, Francis Moïse Dossou, Irani Durán, Rohini Dutta, Dhruva Ghosh, Frank Gyamfi, Parvez Haque, Pollyanna Hardy, Mike Horton, Gabriella Hyman, Ritu Jain, Oluwaseun Ladipo-Ajayi, Ismail Lawani, Souliath Lawani, Mwayi Kachapila, Rachel Lillywhite, Rhiannon Macefield, Laura Magill, Janet Martin, Jonathan Mathers, Kenneth McLean, Punam Mistry, Rohin Mittal, Mark Monahan, Rachel Moore, Dion Morton, Moyo Ojo, Faustin Ntirenganya, Emmanuel Ofori, Rupert Pearse, Alberto Peón, Thomas Pinkney, Antonio Ramos de la Medina, Tubasiime Ronald, David Roman, Emmy Runingamugabo, Alice Sitch, Anita Slade, Donna Smith, Stephen Tabiri, Aneel Bhangu, James Glasbey, Anita Slade, Mike Horton, Rhiannon Macefield, Aneel Bhangu, Pollyanna Hardy, Adesoji O Ademuyiwa, Lawani Ismail, Dhruva Ghosh, Antonio Ramos de la Medina, Rachel Moore, Faustin Ntirenganya, Stephen Tabiri, Emmy Runingamugabo, Simin Patrawala, Angela Prah, Christian Oko, Karolin Kroese, Ismaïl Lawani, Francis Moïse Dossou, Corinne Dzemta, Covalic Melic Bokossa Kandokponou, Souliath Lawani, Hulrich Behanzin, Cyrile Kpangon, Bernard Appiah Ofori, Stephen Tabiri, Abdul-Hafiz Saba, Gbana Limann, Daniel Kwesi Acquah, Shamudeen Mohammed Alhassan, Sheriff Mohammed, Owusu Abem Emmanuel, Yakubu Musah, Yenli Edwin, Sheba Kunfah, Yakubu Mustapha, Abantanga Atindaana Francis, Emmanuel Ayingayure, Gbana Limann, Forster Amponsah-Manu, Eric Agyemang, Vera Agyekum, Esther Adjei-Acquah, Emmanuel Yaw Twerefour, Barbra Koomson, Ruby Acheampong Boateng, Ato Oppong Acquah, Richard Ofosu-Akromah, Leslie Issa Adam-Zakariah, Nii Armah Adu-Aryee, Theodore Wordui, Coomson Christian Larbi, Akosa Appiah Enoch, Mensah Elijah, Kyeremeh Christian, Addo Gyambibi Kwame, Boakye Percy, Kontor Effah Bismark, Gyamfi Brian, Manu Ruth, Romeo Hussey, Samuel Dadzie, Akosua Dwamena Appiah, Grace Yeboah, Cynthia Yeboah, James Amoako, Regina Acquah, Naa Anyekaa Sowah, Atta Kusiwaa, Esther Asabre, Cletus Ballu, Charles Gyamfi Barimah, Frank Owusu, Clement Sie-Broni, Vivian Adobea, Prince Yeboah Owusu, Marshall Zume, Abdul-Hamid Labaran, Raphael Adu-Brobbey, Martin Tangnaa Morna, Samuel A Debrah, Patrick Opoku Manu Maison, Michael Nortey, Donald Enti, Mabel Pokuah Amoako-Boateng, Anthony Baffour Appiah, Emmanuel Owusu Ofori, Richard Kpankpari, Benedict Boakye, Elizabert Mercy Quartson, Patience Koggoh, Anita Eseenam Agbeko, Frank Enoch Gyamfi, Joshua Arthur, Joseph Yorke, Christian Kofi Gyasi-Sarpong, Charles Dally, Agbenya Kobla Lovi, Michael Amoah, Boateng Nimako, Robert Sagoe, Anthony Davor, Fareeda Galley, Michael Adinku, Jonathan Boakye-Yiadom, Jane Acquaye, Juliana Appiah, Dorcas Otuo Acheampong, Iddrisu Haruna, Edward Amoah Boateng, Emmanuel Kafui Ayodeji, Samuel Tuffuor, Naa Kwarley, Yaa Tufuor, Ramatu Darling Abdulai, Fred Dankwah, Ralph Armah, Doris Ofosuhene, Dorcas Osei-Poku, Arkorful Ebenezer Temitope, Delali Akosua Gakpetor, Victoria Sena Gawu, Christopher Asare, Enoch Tackie, James Ankomah, Isaac Omane Nyarko, Zelda Robertson, Serbeh Godwin, Appiah Anthony Boakye, Godfred Fosu, Frank Assah-Adjei, Parvez Haque, Ritu Jain, Alisha Bhatt, Jyoti Dhiman, Rohini Dutta, Dhruva Ghosh, Esther Daniel, K Priyadarshini, Latha Madankumar, Rohin Mittal, Ida Nagomy, Soosan Prasad, Arpit Jacob Mathew, Danita Prakash, Priya Jacob, Jeremiah P Anachy, Amy Mathew, Josy Thomas, Philip V Alexander, Pradeep Zechariah, Neerav D Aruldas, Asif Mehraj, Hafsa Imtiyaz Ahmed, Rauf A Wani, Fazl Q Parray, Nisar A Chowdri, Antonio Ramos De la Medina, Laura Martinez Perez Maldonado, Diana S Gonzalez Vazquez, Iran I Durán Sánchez, Maria J Martínez Lara, Alejandra Nayen Sainz de la Fuente, Ana O Cortes Flores, Mariana E Barreto Gallo, Alejandro Gonzalez Ojeda, Monica E Jimenez Velasco, Luis Hernández Miguelena, Reyes J Cervantes Ortiz, Gonzalo I Hernandez Gonzalez, Marco Hurtado Romero, Rosa I Hernandez Krauss, Luis A Dominguez Sansores, Alejandro Cuevas Avendaño, Celina Cuellar Aguirre, Isaac Baltazar Gomez, Hector Ortiz Mejia, Alejandro González Ojeda, Oscar E Olvera Flores, Erick A González García de Rojas, Kevin J Pintor Belmontes, Francisco J Barbosa Camacho, Aldo Bernal Hernández, Laura Reyes Aguirre, Rubén E Morán Galaviz, Clotilde Fuentes Orozco, Wenceslao G Ángeles Bueno, Fernando S Ramirez Marbello, Diego E Luna Acevedo, Michel Hernández Valadez, Ana L Bogurin Arellano, Luis R Ramírez-González, Bertha G Guzmán Ramírez, Eduardo Valtierra Robles, Ramona I Rojas García, José V Pérez Navarro, Edgar J Cortes Torres, David R Dominguez Solano, Alberto N Peón, Roque D Lincona Menindez, Rozana Reyes Gamez, Maria C Paz Muñoz, Orimisan Belie, Victoria Adeleye, Adesoji Ademuyiwa, Oluwafunmilayo Adeniyi, Opeyemi Akinajo, David Akinboyewa, Felix Alakaloko, Oluwole Atoyebi, Olanrewaju Balogun, Christopher Bode, Olumide Elebute, Francis Ezenwankwo, Adesiyakan Adedotun, George Ihediwa, Jubril Kuku, Oluwaseun Ladipo-Ajayi, Ayomide Makanjuola, Samuel Nwokocha, Olubunmi Ogein, Rufus Ojewola, Abraham Oladimeji, Thomas Olajide, Iyabo Alasi, Oluwaseun Oluseye, Justina Seyi-Olajide, Adaiah Soibi-Harry, Emmanuel Williams, Agbulu Moses Vincent, Nnamdi Jonathan Duru, Kenneth Uche Onyekachi, Christiana Ashley, Chinelo Victoria Mgbemena, Moyosoluwa Ojo, Olowu Oluyemisi, Iyabode Ikuewunmi, Adeoluwa Adebunmi, Edet Glory Bassey, Ephraim Okwudiri Ohazurike, Olayide Michael Amao, Osunwusi Benedetto Oluwaseun, Emily Doris, Olutola Stephen, Christianah Gbenga-Oke, Olawunmi Olayioye, Olowu Oluyemisi, Kayode Oluremi, Esther Abunimye, Christianah Oyegbola, Olayade Kayode, Adeola Ayoola Orowale, Omolara M Williams, Olufunmilade A Omisanjo, Omolara M Faboya, Zainab O Imam, Olabode A Oshodi, Yusuf A Oshodi, Ayokunle A Ogunyemi, Olalekan T Ajai, Francisca C Nwaenyi, Adewale O Adisa, Adewale A Aderounmu, Funmilola O Wuraola, Oludayo Sowande, Lukman Olajide Abdur-Rahman, Jibril Oyekunle Bello, Hadijat Olaide Raji, Nurudeen Abiola Adeleke, Saheed Abolade Lawal, Rafiat Tinuola Afolabi, Abdulwahab Lawal, Okechukwu Hyginus Ekwunife, Ochomma Amobi Egwuonwu, Chisom Faith Uche, Abubakar Bala AB Muhammad, Saminu S Muhammad, Idris Usman IU Takai, Mohammed AS Aliyu Salele, Onyekachi G Ukata, Mahmoud Kawu MK Magashi, Lawal Barau LB Abdullahi, Bello Abodunde BA Muideen, Khadija A Ado, Lofty-John Chukwuemeka LJC Anyawu, Samson Olori, Samuel A Sani, Olabisi O Osagie, Ndubuisi Mbajiekwe, Oseremen Aisuodionoe-Shadrach, Godwin O Akaba, Lazarus Ameh, Lazarus Ameh, Francis o Adebayo, Martins Uanikhoba, Felix O Ogbo, Nancy O Tabuanu, Taiwo A Lawal, Rukiyat A Abdus-Salam, Akinlabi E Ajao, Augustine O Takure, Omobolaji O Ayandipo, Hyginus O Ekwuazi, Olukayode Abayomi, Olatunji O Lawal, Solomon Olagunju, Kelvin I Egbuchulem, Sikiru Adekola Adebayo, Peter Elemile, Usang E Usang, Joseph E Udosen, Expo E Edet, Akan W Inyang, Edima M Olory, Gabriel U Udie, Godwin O Chiejina, Adams D Marwa, Faith J Iseh, Sunday A Ogbeche, Mary O Isa, Uchechukwu O Ezomike, Sebastian O Ekenze, Matthew I Eze, Emmanuel O Izuka, Jude K Ede, Vincent C Enemuo, Okezie M Mbadiwe, Ngozi G Mbah, Alphonsine Imanishimwe, Sosthene Habumuremyi, Faustin Ntirenganya, JC Allen Ingabire, Isaie Ncogoza, Emmanuel Munyaneza, Jean de Dieu Haragirimana, Christian Jean Urimubabo, Violette Mukanyange, Jeannette Nyirahabimana, Emmanuel Mutabazi, Christophe Mpirimbanyi, Olivier Mwenedata, Hope Lydia Maniraguha, Emelyne Izabiriza, Moses Dusabe, Job Zirikana, Francine Uwizeyimana, Josiane Mutuyimana, Elisee Rwagahirima, Alphonsine Imanishimwe, Ronald Tubasiime, Aphrodis Munyaneza, Sosthene Habumuremyi, Salathiel Kanyarukiko, Gibert Ndegamiye, Francine Mukaneza, Jean Claude Uwimana, Pierrine Nyirangeri, Deborah Mukantibaziyaremye, Aime Dieudonne Hirwa, Salomee Mbonimpaye, Piolette Muroruhirwe, Christine Mukakomite, Elysee Kabanda, Rachel Moore, Ncamsile Anthea Nhlabathi, Maria Fourtounas, Mary Augusta Adams, Gabriella Hyman, Hlengiwe Samkelisiwe Nxumalo, Nnosa Sentholang, Mmule Evelyn Sethoana, Mpho Nosipho Mathe, Zain Ally, Margot Flint, Bruce Biccard, Adesoji O Ademuyiwa, Adewale O Adisa, Aneel Bhangu, Peter Brocklehurst, Sohini Chakrabortee, Pollyanna Hardy, Ewen Harrison, JC Allen Ingabire, Parvez D Haque, Lawani Ismail, James Glasbey, Dhruva Ghosh, Frank Enoch Gyamfi, Elizabeth Li, Rachel Lillywhite, Antonio Ramos de la Medina, Rachel Moore, Laura Magill, Dion Morton, Dmitri Nepogodiev, Faustin Ntirenganya, Thomas Pinkney, Omar Omar, Joana Simoes, Donna Smith, Stephen Tabiri, Adesoji O Ademuyiwa, Lawani Ismail, Dhruva Ghosh, Antonio Ramos de la Medina, Rachel Moore, Faustin Ntirenganya, Stephen Tabiri, Adesoji Ademuyiwa, Aneel Bhangu, Felicity Brant, Peter Brocklehurst, Sohini Chakrabortee, Dhruva Ghosh, James Glasbey, Pollyanna Hardy, Ewen Harrison, Emily Heritage, Lawani Ismail, Karolin Kroese, Carmela Lapitan, Rachel Lillywhite, David Lissauer, Laura Magill, Antonio Ramos de la Medina, Punam Mistry, Mark Monahan, Rachel Moore, Dion Morton, Dmitri Nepogodiev, Faustin Ntirenganya, Omar Omar, Thomas Pinkney, Tracy Roberts, Donna Smith, Stephen Tabiri, Neil Winkles, Pollyanna Hardy, Omar Omar, Emmy Runigamugabo, Azmina Verjee, Pierre Sodonougbo, Pamphile Assouto, Michel Fiogbe, Houenoukpo Koco, Serge Metchinhoungbe, Hodonou Sogbo, Hulrich Behanzin, Djifid Morel Seto, Yannick Tandje, Sosthène Kangni, Cyrile Kpangon, Marcelin Akpla, Hugues Herve Chobli, Blaise Kovohouande, Gérard Agboton, Rene Ahossi, Raoul Baderha Ngabo, Nathan Bisimwa, Covalic Melic Bokossa Kandokponou, Mireille Dokponou, Francis Moïse Dossou, Corinne Dzemta, Antoine Gaou, Roland Goudou, Emmanuel Hedefoun, Sunday Houtoukpe, Felix Kamga, Eric Kiki-Migan, Souliath Lawani, Ismaïl Lawani, René Loko, Afissatou Moutaïrou, Pencome Ogouyemi, Fouad Soumanou, Pia Tamadaho, Mack-Arthur Zounon, Luke Aniakwo Adagrah, Bin Baaba Alhaji Alhassan, Mabel Pokuah Amoako-Boateng, Anthony Baffour Appiah, Alvin Asante-Asamani, Benedict Boakye, Samuel A Debrah, Donald Enti, Rahman Adebisi Ganiyu, Patience Koggoh, Richard Kpankpari, Isabella Naa M Opandoh, Meshach Agyemang Manu, Maison Patrick Opoku Manu, Samuel Mensah, Martin Tangnaa Morna, John Nkrumah, Michael Nortey, Emmanuel Owusu Ofori, Elizaberth Mercy Quartson, Esther Adjei-Acquah, Vera Agyekum, Eric Agyemang, Rebecca Adjeibah Akesseh, Forster Amponsah-Manu, Richard Ofosu-Akromah, Ato Oppong Acquah, Leslie Issa Adam-Zakariah, Esther Asabre, Ruby Acheampong Boateng, Barbara Koomson, Ataa Kusiwaa, Emmanuel Yaw Twerefour, James Ankomah, Frank Assah-Adjei, Anthony Appiah Boakye, Godfred Fosu, Godwin Serbeh, Kofi Yeboah Gyan, Isaac Omane Nyarko, Zelda Robertson, Ralph Armah, Christopher Asare, Delali Akosua Gakpetor, Victoria Sena Gawu, Ambe Obbeng, Doris Ofosuhene, Dorcas Osei-Poku, Diana Puozaa, Enoch Tackie, Arkorful Ebenezer Temitope, Regina Acquah, James Amoako, Akosua Dwamena Appiah, Mark Aseti, Charles Banka, Samuel Dadzie, Derick Essien, Frank Enoch Gyamfi, Romeo Hussey, Jemima Kwarteng, Naa Anyekaa Sowah, Grace Yeboah, Cynthia Yeboah, Kwame Gyambibi Addo, Enoch Appiah Akosa, Percy Boakye, Christian Larbi Coompson, Brian Gyamfi, Bismark Effah Kontor, Christian Kyeremeh, Ruth Manu, Elijah Mensah, Friko Ibrahim Solae, Gideon Kwasi Toffah, Dorcas Otuo Acheampong, Jane Acquaye, Michael Adinku, Kwabena Agbedinu, Anita Eseenam Agbeko, Emmanuel Gyimah Amankwa, Michael Amoah, George Amoah, Juliana Appiah, Joshua Arthur, Alex Ayim, Emmanuel Kafui Ayodeji, Jonathan Boakye-Yiadom, Edward Amoah Boateng, Charles Dally, Anthony Davor, Christian Kofi Gyasi-Sarpong, Naabo Nuhu Noel Hamidu, Iddrisu Haruna, Naa Kwarley, Agbenya Kobla Lovi, Boateng Nimako, Bertina Beauty Nyadu, Dominic Opoku, Anita Osabutey, Robert Sagoe, Samuel Tuffour, Yaa Tufour, Francis Akwaw Yamoah, Abiboye Cheduko Yefieye, Joseph Yorke, Nii Armah Adu-Aryee, Faisal Adjei, Erica Akoto, Elikem Ametefe, Joachim Kwaku Amoako, Godsway Solomon Attepor, George Darko Brown, Benjamin Fenu, Philemon Kwame Kumassah, David Olatayo Olayiwola, Theodore Wordui, Nelson Agboadoh, Fatao Abubakari, Cletus Ballu, Charles Gyamfi Barimah, Guy Casskey Boateng, Prosper Tonwisi Luri, Abraham Titigah, Frank Owusu, Raphael Adu-Brobbey, Christian Larbi Coompson, Abdul-Hamid Labaran, Junior Atta Owusu, Vivian Adobea, Amos Bennin, Fred Dankwah, Stanley Doe, Ruth Sarfo Kantanka, Ephraim Kobby, Kennedy Kofi Korankye Hanson Larnyor, Edwin Osei, Prince Yeboah Owusu, Clement Ayum Sie-Broni, Marshall Zume, Francis Atindaana Abantanga, Darling Ramatu Abdulai, Daniel Kwesi Acquah, Emmanuel Ayingayure, Imoro Osman, Sheba Kunfah, Gbana Limann, Shamudeen Alhassan Mohammed, Sheriff Mohammed, Yakubu Musah, Bernard Ofori, Emmanuel Abem Owusu, Abdul-Hafiz Saba, Anwar Sadat Seidu, Stephen Tabiri, Mustapha Yakubu, Edwin Mwintiereh Taang Yenli, Arun Gautham, Alice Hepzibah, Grace Mary, Deepak Singh, Dimple Bhatti, William Bhatti, Karan Bir, Swati Daniel, Tapasya Dhar, Jyoti Dhiman, Dhruva Ghosh, Sunita Goyal, Ankush Goyal, Monika Hans, Parvez Haque, Samuel Konda, Anil Luther, Amit Mahajan, Shalini Makkar, Kavita Mandrelle, Vishal Michael, Partho Mukherjee, Reuben Rajappa, Prashant Singh, Atul Suroy, Ravinder Thind, Alen Thomas, Arti Tuli, Sreejith Veetil, Esther Daniel Mark Jesudason, K Priyadarshini, Latha Madankumar, Rohin Mittal, Ida Nagomy, Rajesh Selvakumar, Bharat Shankar, Moonish Sivakumar, Rajeevan Sridhar, Cecil Thomas, Devabalan Titus, Manisha Aggarwal, Parth Dhamija, Himani Gupta, Vinoth Kanna, Ashwani Kumar, Gurtaj Singh, Philip Alexander, Josy Thomas, Pradeep Zechariah, Amos Dasari, Priya Jacob, Elizabeth Kurien, Arpit Mathew, Danita Prakash, Anju Susan, Rose Varghese, Rahul Alpheus, Ashish Choudhrie, Hemanth Kumar, Nitin Peters, Subrat Raul, Rajeev Sharma, Rakesh Vakil, Wenceslao Ángeles Bueno, Francisco Barbosa Camacho, Aldo Bernal Hernández, Ana Bogurin Arellano, Edgar Cortes Torres, Clotilde Fuentes Orozco, Erick González García de Rojas, Alejandro González Ojeda, Bertha Guzmán Ramírez, Michel Hernández Valadez, Diego Luna Acevedo, Rubén Morán Galaviz, Oscar Olvera Flores, José Pérez Navarro, Kevin Pintor Belmontes, Fernando Ramirez Marbello, Luis Ramírez-González, Laura Reyes Aguirre, Ramona Rojas García, Eduardo Valtierra Robles, Reyes Ortiz Cervantes, Gonzalo Hernandez Gonzalez, Rosa Hernandez Krauss, Luis Hernández Miguelena, Marco Hurtado Romero, Isaac Baltazar Gomez, Celina Cuellar Aguirre, Alejandro Cuevas Avendaño, Luis Dominguez Sansores, Hector Ortiz Mejia, Laura Urdapilleta Gomez del Campo, Claudia Caballero Cerdan, David Dominguez Solano, Rafael Toriz Garcia, Mariana Barreto Gallo, Ana Cortes Flores, Alejandro Gonzalez Ojeda, Monica Jimenez Velasco, Rozana Reyes Gamez, Roque Lincona Menindez, Alberto Navarrete Peón, Maria Paz Muñoz, Irán Irani Durán Sánchez, Diana Samantha González Vázquez, María José Martínez Lara, Laura Martinez Perez Maldonado, Alejandra Nayen Sainz de la Fuente, Antonio Ramos De la Medina, Lawal Abdullahi, Khadija Ado, Mohammed Aliyu, Lofty-John Anyanwu, Mahmoud Magashi, Abubakar Muhammad, Saminu Muhammad, Bello Muideen, Idris Takai, Onyekachi Ukata, Opeoluwa Adesanya, David Awonuga, Olushola Fasiku, Chidiebere Ogo, Moruf Abdulsalam, Abimbola Adeniran, Olalekan Ajai, Olukemi Akande, Kazeem Atobatele, Grace Eke, Omolara Faboya, Zainab Imam, Esther Momson, Francisca Nwaenyi, Ayokunle Ogunyemi, Mobolaji Oludara, Olufunmilade Omisanjo, Olabode Oshodi, Yusuf Oshodi, Yemisi Oyewole, Omotade Salami, Omolara Williams, Victoria Adeleye, Adesoji Ademuyiwa, Oluwafunmilayo Adeniyi, Opeyemi Akinajo, David Akinboyewa, Iyabo Alasi, Felix Alakaloko, Oluwole Atoyebi, Olanrewaju Balogun, Orimisan Belie, Christopher Bode, Andrew Ekwesianya, Olumide Elebute, Francis Ezenwankwo, Adedeji Fatuga, George Ihediwa, Adesola Jimoh, Jubril Kuku, Oluwaseun LadipoAjayi, Ayomide Makanjuola, Olayanju Mokwenyei, Samuel Nwokocha, Olubunmi Ogein, Rufus Ojewola, Abraham Oladimeji, Thomas Olajide, Oluwaseun Oluseye, Justina Seyi-Olajide, Adaiah Soibi-Harry, Aloy Ugwu, Emmanuel Williams, Ochomma Egwuonwu, Okechukwu Ekwunife, Victor Modekwe, Chukwuemeka Okoro, Chisom Uche, Kenneth Ugwuanyi, Chuka Ugwunne, Akeem Adeleke, Wilson Adenikinju, Olumide Adeniyi, Akinfolarin Adepiti, Adewale Aderounmu, Abdulhafiz Adesunkanmi, Adewale Adisa, Samuel Ajekwu, Olusegun Ajenjfuja, Jerrie Akindojutimi, Akinbolaji Akinkuolie, Olusegun Alatise, Olubukola Allen, Lukmon Amosu, Micheal Archibong, Olukayode Arowolo, Deborah Ayantona, Ademola Ayinde, Olusegun Badejoko, Tajudeen Badmus, Amarachukwu Etonyeaku, Emeka Igbodike, Omotade Ijarotimi, Adedayo Lawal, Fayowole Nana, Tunde Oduanafolabi, Olalekan Olasehinde, Olaniyi Olayemi, Stephen Omitinde, Owolabi Oni, Chigozie Onyeze, Ernest Orji, Adewale Rotimi, Abdulkadir Salako, Olufemi Solaja, Oluwaseun Sowemimo, Ademola Talabi, Mohammed Tajudeen, Funmilola Wuraola, Francis Adebayo, Oseremen Aisuodionoe-Shadrach, Godwin Akaba, Lazarus Ameh, Ndubuisi Mbajiekwe, Felix Ogbo, Samson Olori, Olabisi Osagie, Abu Sadiq, Samuel Sani, Nancy Tabuanu, Martins Uanikhoba, Godwin Chiejina, Ekpo Edet, Akan Inyang, Mary Isa, Faith Iseh, Adams Marwa, Sunday Ogbeche, Edima Olory, Gabriel Udie, Joseph Udosen, Usang Usang, Olukayode Abayomi, Rukiyat Abdus-Salam, Sikiru Adebayo, Akinlabi Ajao, Olanrewaju Amusat, Omobolaji Ayandipo, Kelvin Egbuchulem, Hyginus Ekwuazi, Peter Elemile, Taiwo Lawal, Olatunji Lawal, Solomon Olagunju, Peter Osuala, Bamidele Suleman, Augustine Takure, Lukman Abdur-Rahman, Nurudeen Adeleke, Muideen Adesola, Rafiat Afolabi, Sulaiman Agodirin, Isiaka Aremu, Jibril Bello, Saheed Lawal, Abdulwahab Lawal, Hadijat Raji, Olayinka Sayomi, Asimiyu Shittu, Jude Ede, Sebastian Ekenze, Vincent Enemuo, Matthew Eze, Uchechukwu Ezomike, Emmanuel Izuka, Okezie Mbadiwe, Ngozi Mbah, Uba Ezinne, Matthew Francis, Iweha Ikechukwu, Okoi Nnyonno, Philemon Okoro, Igwe Patrick, John Raphael, Oriji Vaduneme, Abhulimen Victor, Salathiel Kanyarukiko, Francine Mukaneza, Deborah Mukantibaziyaremye, Aphrodis Munyaneza, Gibert Ndegamiye, Ronald Tubasiime, Moses Dusabe, Emelyne Izabiriza, Hope Lydia Maniraguha, Christophe Mpirimbanyi, Josiane Mutuyimana, Olivier Mwenedata, Elisee Rwagahirima, Francine Uwizeyimana, Job Zirikana, Aime Dieudonne Hirwa, Elysee Kabanda, Salomee Mbonimpaye, Christine Mukakomite, Piolette Muroruhirwe, Georges Bucyibaruta, Gisele Juru Bunogerane, Sosthene Habumuremyi, Jean de Dieu Haragirimana, Alphonsine Imanishimwe, JC Allen Ingabire, Violette Mukanyange, Emmanuel Munyaneza, Emmanuel Mutabazi, Isaie Ncogoza, Faustin Ntirenganya, Jeannette Nyirahabimana, Christian Urimubabo, Mary Augusta Adams, Richard Crawford, Chikwendu Jeffrey Ede, Maria Fourtounas, Gabriella Hyman, Zafar Khan, Morapedi Kwati, Mpho Nosipho Mathe, Rachel Moore, Ncamsile Anthea Nhlabathi, Hlengiwe Samkelisiwe Nxumalo, Paddy Pattinson, Nnosa Sentholang, Mmule Evelyn Sethoana, Maria Elizabeth Stassen, Laura Thornley, Paul Wondoh Edenvale Hospital, Cheryl Birtles, Mathete Ivy, Cynthia Mbavhalelo, Zain Ally, Abdus-sami Adewunmi, Jonathan Cook, David Jayne, Soren Laurberg, Julia Brown, Simon Cousens, Neil Smart

**Affiliations:** NIHR Global Health Research Unit on Global Surgery, University of Birmingham, Institute of Translational Medicine, Birmingham, UK

## Abstract

**Background:**

The Bluebelle Wound Healing Questionnaire (WHQ) is a universal-reporter outcome measure developed in the UK for remote detection of surgical-site infection after abdominal surgery. This study aimed to explore cross-cultural equivalence, acceptability, and content validity of the WHQ for use across low- and middle-income countries, and to make recommendations for its adaptation.

**Methods:**

This was a mixed-methods study within a trial (SWAT) embedded in an international randomized trial, conducted according to best practice guidelines, and co-produced with community and patient partners (TALON-1). Structured interviews and focus groups were used to gather data regarding cross-cultural, cross-contextual equivalence of the individual items and scale, and conduct a translatability assessment. Translation was completed into five languages in accordance with Mapi recommendations. Next, data from a prospective cohort (SWAT) were interpreted using Rasch analysis to explore scaling and measurement properties of the WHQ. Finally, qualitative and quantitative data were triangulated using a modified, exploratory, instrumental design model.

**Results:**

In the qualitative phase, 10 structured interviews and six focus groups took place with a total of 47 investigators across six countries. Themes related to comprehension, response mapping, retrieval, and judgement were identified with rich cross-cultural insights. In the quantitative phase, an exploratory Rasch model was fitted to data from 537 patients (369 excluding extremes). Owing to the number of extreme (floor) values, the overall level of power was low. The single WHQ scale satisfied tests of unidimensionality, indicating validity of the ordinal total WHQ score. There was significant overall model misfit of five items (5, 9, 14, 15, 16) and local dependency in 11 item pairs. The person separation index was estimated as 0.48 suggesting weak discrimination between classes, whereas Cronbach’s α was high at 0.86. Triangulation of qualitative data with the Rasch analysis supported recommendations for cross-cultural adaptation of the WHQ items 1 (redness), 3 (clear fluid), 7 (deep wound opening), 10 (pain), 11 (fever), 15 (antibiotics), 16 (debridement), 18 (drainage), and 19 (reoperation). Changes to three item response categories (1, not at all; 2, a little; 3, a lot) were adopted for symptom items 1 to 10, and two categories (0, no; 1, yes) for item 11 (fever).

**Conclusion:**

This study made recommendations for cross-cultural adaptation of the WHQ for use in global surgical research and practice, using co-produced mixed-methods data from three continents. Translations are now available for implementation into remote wound assessment pathways.

## Teaser

This mixed-methods study within the FALCON trial across six low-and middle-income countries used qualitative and state-of-the-art psychometric methods with Rasch analysis. The authors make recommendations for adaptation of a Wound Healing Questionnaire (originally published in *BJS* in 2019) to detect wound infections remotely after abdominal surgery over the telephone in global surgery research and practice. This tool can help to upscale telemedicine safely in low-resource environments.

## Introduction

Surgical-site infection (SSI) is the most common complication of abdominal surgery, and has a cross-societal, global impact on patients and their families^1–5^. Delayed return to work, readmission or reoperation leads to substantial effects on quality of life during recovery, and has spill-over effects on mental, economic, and social well-being for patients^6^. This is particularly relevant in low-resource settings, where patients are more likely to suffer catastrophic expenditure around the time of surgery^7^. Consequently, research in SSI prevention has been prioritized by patients, researchers, and clinicians in low- and middle-income countries (LMICs)^8^.

Timely identification of SSI is essential in maintaining patient safety after hospital discharge. Missed SSI diagnoses or misclassification of SSI can directly and indirectly affect patient safety^9^: directly, through delayed intervention for patients with active infection, and indirectly, by introducing bias to randomized studies that feed into best practice guidelines^3,10^. Postdischarge surveillance is therefore considered to be a key quality marker in SSI research and is an important component of postoperative care pathways^10^.

The Bluebelle Wound Healing Questionnaire (WHQ) was developed and validated in the UK in the English language to support postdischarge surveillance for SSI after abdominal surgery^11,12^. This instrument has, however, not yet been adapted for cross-cultural and cross-language implementation in LMICs. High-quality, contextually relevant tools for remote wound evaluation are urgently needed to build resilient and sustainable surgical systems and support safe upscaling of capacity during pandemic recovery^13,14^. They are also needed to reduce loss to follow-up and risk of attrition bias in randomized trials by developing contextually relevant pathways for remote assessment^9^.

The aims of this mixed-methods study (TALON-1) were: to explore cross-cultural and cross-language equivalence, acceptability, and content validity of the WHQ across several LMICs; to assess the scaling and psychometric properties of the WHQ when used across different patient populations and subgroups using Rasch analysis; and to consolidate recommendations for adaptation of the WHQ for use in global surgical research by triangulating qualitative and quantitative data.

## Methods

**Fig. 1:**
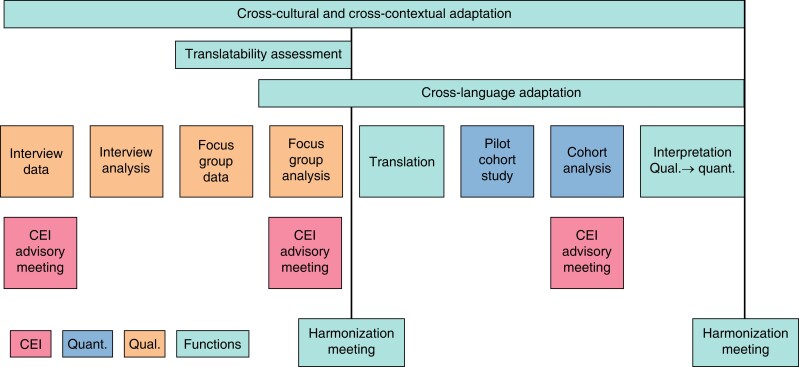
Overview of TALON-1 study methods

TALON-1 was a mixed-methods study embedded in an international randomized trial, conducted according to best practice guidelines, and co-produced with community and patient partners^15–17^. The study used qualitative and quantitative data to explore the extent to which the WHQ measured SSI as a concept, and the parameters of the latent trait (that is, an underlying outcome of interest) in the target (low-resource context) and source (the UK, a high-resource universal healthcare system) cultures. It then aimed to assess how accurately items could transfer meaning across languages^18^. Some adaptation of standard methodology was required to enable the qualitative phase to progress during the SARS-CoV-2 pandemic (*Appendix S2*). An overview of the study methodology is shown in *Fig. 1* and detailed in *Table 1*.

First, an expert review of the Wound Healing Questionnaire (WHQ) was conducted using structured interviews and focus groups with surgeons, and site researchers involved in wound evaluation were used to gather rich data regarding cross-cultural, cross-contextual equivalence of the individual items and scale, and conduct a baseline translatability assessment. Second, data from a prospective cohort study were interpreted using a Rasch unidimensional measurement modelling approach to explore scaling and measurement properties of the questionnaire, including cross-cultural differential item functioning. Next, qualitative and quantitative data were triangulated using a modified, exploratory, instrumental design model to recommend adaptations for use of the WHQ in global surgery research and practice^19^. Finally, translation into five languages was completed in accordance with Mapi recommendations. CEI, community engagement and involvement; qual., qualitative; quant., quantitative.

**Table 1:** Summary of Wound Healing Questionnaire adaptation methodology

Methodology	Yes	No	Details
Concept definition (protocol)	Y		Protocol agreed between international Study Management Group, developers, community, and patient partners. Preregistered and published in *Trials* ^20^ and in SWAT store registry (ID126)
**Qualitative phase: cross-cultural and cross-contextual adaptation (in source language, English)**			
ȃConsultant identified	Y		An in-country consultant was identified in each target country who was fluent in both the source and target language(s). This was typically the national principal investigator (a surgeon involved directly in wound assessment) for the study, or else a clinical nominee
ȃStructured interviews (expert review)	Y		Structured interviews were designed to review the instrument validity, items, and scaling. The topic guide was directed item by item, learning from cognitive theory. In each country, 2–3 interviews were conducted with site investigators directly involved in wound assessment
ȃFocus groups (reconciliation and translatability assessment)	Y		A focus group was held with each country to review coding and analysis from the expert review phase (member checking). This included several investigators fluent in both the source and target language. An item-by-item translatability assessment was made in parallel. Any further iterative modifications were made before moving into the harmonization meeting
ȃCommunity and patient partner review	Y		Patient advisory group meeting with representation from 4 of the target countries (Nigeria, South Africa, India, Ghana) was convened to review the recommendations for adaptation of the instrument item by item, and to co-design the cohort study including co-production of the telephone follow-up pathway and supporting documentation
ȃHarmonization meeting	Y		Virtual meeting on Zoom platform with national principal investigators to sign off final adaptation of the adapted English language WHQ to move into cross-language translation
**Cross-language translation (performed for each target language)**			
ȃDual forward translations	Y		Performed by translators fluent in both the source and target language, and native to the target country
ȃForward translation reconciliation	Y		Comparison of translations with any discrepancies resolved with discussion between translators and in-country consultant
ȃBack translation × 1	Y		Performed by translator fluent in both the source and target language, and native to the target country
ȃBack translation reconciliation	Y		Comparison of back-translated source language questionnaire with original. Discussion within in-country consultant to review and resolve any consistencies
ȃDeveloper’s review	Y		Developers collaborated as members of the Study Management Group and co-authors of this manuscript
ȃCognitive interviewing (replaced with data review)		N	Cognitive interviewing with patients was not possible during SARS-CoV-2. Qualitative data from the expert review and transability assessment were used instead to inform translation, led by the consultant
ȃClinician review and proofreading	Y		Clinicians involved in wound care embedded in the adaptation and translation process. Two native speaking clinicians provided the final review and proofreading
ȃPilot testing	Y		Target language delivery tested during follow-up with 5–10 patients to test comprehension, phrasing, and delivery. A monitoring call was held with the investigators to review feedback before progression to the quantitative study
ȃHarmonization meeting	Y		Virtual meeting on Zoom platform with national principal investigators to act as a final quality check and share review lessons learnt during translation
ȃPublished	Y		Final version published included in *Table 3* (source language) and supplementary material (target languages)
**Quantitative phase: cohort study of adapted and translated WHQ (source and target languages)**			
ȃCohort study	Y		Study within a trial within the FALCON RCT to test feasibility, acceptability, and measurement properties of WHQ. Minimum sample size target of 100 patients per country
ȃRasch analysis of cohort study data	Y		Rasch unidimensional measurement modelling in data used to evaluate scaling, measurement properties, and differential item functioning across key subgroups
**Reconciliation and reporting**			
ȃTriangulation	Y		Triangulation of qualitative and quantitative data to inform final recommendations for WHQ adaptation
ȃCommunity and patient partner review	Y		Presentation of findings of cohort study to patient advisory group to co-interpret patterns in data and share insight on final recommendations for WHQ adaptation. Co-production of a lay abstract summary of the research findings for dissemination to the public
ȃFinal harmonization meeting	Y		Virtual meeting on Zoom platform with national principal investigators
ȃValidation report	Y		A full prospective validation study for the adapted global WHQ in the target languages in 7 low- and middle-income countries is reported elsewhere.

Adapted from Oxford University Innovation outcomes centre checklist, and Mapi process for cross-cultural and cross-language adaptation. SWAT, study within a trial; WHQ, Wound Healing Questionnaire.

### Reporting and registration

This study was reported with reference to recommendations from the Global Health Network for qualitative research in LMICs, the COREQ framework^15,21^, and PCORI recommendations^16^ for best practice in mixed-methods adaptation of outcome measures (PCORI checklist is available in *Appendix S3*). Primary data from FALCON were published in *The Lancet* in 2021^22^. The protocol for TALON-1 was preregistered on the MRC Hubs for Trial Methodology Research database^23^ (Queen’s University Belfast) (SWAT ID126) and published in *Trials*^20^.

### Ethics and ethical approvals

This study within a trial (SWAT) was first approved within the pragmatic multicentre factorial RCT testing measures to reduce SSI in LMICs (FALCON trial) protocol by a University of Birmingham Research Ethics Committee (v1_0_substudies_v1_0. Reference: ERN_18-0230A). Additional approvals were then obtained from national, regional, and/or hospital-level ethics committees for selected centres in all participating countries, in accordance with local protocols. Written (or fingerprint) informed consent to participate was obtained from all participants. In the qualitative phase, an information sheet for was provided to all participants. Verbal consent was taken and recorded. Participant data were pseudonymized for storage securely within a password-protected NVivo® V12 data management system. In the quantitative phase, written (or fingerprint) informed consent to participate was obtained from all participants. Quantitative data were stored in a secure REDCap server^24^, hosted at the University of Birmingham, UK, and held in line with General Data Protection Regulation principles.

### Host trial

FALCON was a stratified, pragmatic, multicentre, 2 × 2 factorial trial testing two measures (skin preparation and antimicrobial sutures) to reduce superficial or deep skin infection after abdominal surgery in seven LMICs (NCT03700749)^1^. FALCON provided a platform for this study both to identify eligible site investigators for interviews and focus groups, and for co-recruitment of patients to the embedded prospective cohort study.

### Study instrument

The WHQ was developed with the aim of detecting postdischarge SSI after abdominal surgery, and validated in a large feasibility study within a pilot RCT (Bluebelle) in the UK, as summarized in *Appendix S4*^12,25,26^. The WHQ includes 19 items (18 items and 1 subitem) related to the construct of surgical wound healing, with 11 items (10 items and 1 conditional subitem) related to symptoms of SSI, and 8 items related to interaction with the treatment pathway for SSI. It was designed so that it could either be administered by a healthcare professional, or self-reported by patients^27^ (a universal-reporter outcome measure^28^). Two developers of the WHQ were collaborating members of the Study Management Group.

### Qualitative phase

#### Cross-cultural and cross-contextual adaptation

Owing to the number of target languages for questionnaire in the host trial, cross-cultural adaptation was initially performed in English language. Structured interviews were conducted with two to three research staff in each country, according to a template from the Social Research Association based on Willis^29^. Participants were purposefully sampled from sites participating in the FALCON trial (research nurses, or doctors directly involved in postoperative wound assessment), with a view to including an information-rich mix of participants by sex, country, patient population (urban/rural home location), and experience in face-to-face and telephone follow-up assessments. These interviews aimed to explore the universality of the construct of SSI, cross-cultural relevance of concepts, and construct validity of the questionnaire^18^.

The topic guide was structured around four predefined categories (*Appendix S5*): item comprehension (patients’ understanding of the idea and item), response mapping (relating a patients’ internally generated answer to response categories provided), retrieval (patients’ ability to remember and recall their response), and judgement (patients’ overall ability to respond to the item and how they came to this answer)^29^. Unstructured interview notes and a reflexive diary were also maintained as an additional data source. Coding was performed using a pragmatic qualitative approach informed by cognitive theory, by a clinician with training in relevant qualitative research methods and with 10 years’ experience of working in international multicentre trials (*Appendix S6*). The reflexive diary supported interpretation of the interviewer’s role as a questionnaire developer and the potential impact on data collection. To ensure credibility, member checking was undertaken with the final summary themes with representative participants and in-country consultants to ensure that meaning was correctly interpreted and maintained^30^.

To check trustworthiness, one or two focus groups were then held with investigators from each country to review and discuss the thematic coding. The focus groups were held after the interviews had been completed to explore consensus and contrasting opinions between different stakeholders around themes emerging in the semistructured interviews. The overall objective was to obtain a single cross-culturally adapted questionnaire to move into cross-language adaptation^31,32^. They were conducted in the English language and co-led by the lead researcher, with one or more in-country consultant co-leads, and sampled 8–12 participants, adopting purposive sampling criteria similar to those of the structured interviews (based on sex, country, patient population, and research experience). A new sample of participants (separate from those participating in interviews) was approached for the focus group phase. Where required, iterative adaptation of the WHQ was made until a point of saturation was reached according to accepted best practice principles for adaptation of instruments^16,33,34^. Recommendations from the qualitative phase were either made overall, specific to an individual item, or related to questionnaire administration. The focus group also included several investigators who were fluent in both the source and target language to serve as a baseline translatability assessment. Together, the process produced an English language questionnaire which had been adapted to broadly ensure cross-cultural equivalence across the participating countries, was acceptable to all national principal investigators, and highlighting potential translatability issues during cross-language adaptation. The procedures for remote, telephone administration of the WHQ were also explored using targeted questions based on investigators’ experience within the FALCON trial.

#### Cross-language adaptation

In some countries, English was a primary or prevalent secondary language among the host trial participants. In these countries, the feasibility of single-language administration of the questionnaire was tested at sites during the cohort study. Where translation of the WHQ was required, this was performed according to the Mapi process for standard linguistic validation to verify conceptual equivalence across languages^34–36^. This involved a seven-step process alongside clinicians directly involved in wound assessment (*Appendix S7)*.

### Quantitative phase

Data for the quantitative phase were collected during a prospective, international cohort SWAT. Consecutive adult patients (aged over 18 years) recruited to the FALCON trial were eligible. These included a broad range of abdominal operations with a predicted clean-contaminated, contaminated or dirty operating field, and a planned skin incision of greater than 5 cm. Operations could be performed for benign, malignant, trauma, or obstetric indications. Consent for an additional telephone follow-up call to administer the WHQ was taken at the same time as trial consent, using a targeted Informed Consent Form and Patient Information Sheet. Patient and community partners supported co-production of these resources to ensure culturally attuned language and delivery.

Telephone administration of the translated WHQ was undertaken 28–30 days after surgery (in the 72 h before in-person follow-up) integrated into the host trial pathway. The telephone WHQ was administered by a researcher, doctor, or research nurse (non-consultant or attending grade), who was independent of the assessment for the trial primary outcome at 30 days after surgery. Optimization and quality assurance of WHQ administration is described in *Appendix S8*. No minimum sample size was set, but a target of 100 patients per country was discussed with each of the national principal investigators for use in Rasch unidimensional measurement modelling, based on published recommendations^37^.

#### Psychometric testing using Rasch analysis

A simple summary of Rasch methodology for the general reader is provided in *Appendix S9*.

The Rasch unidimensional measurement model was fitted to examine the psychometric properties of the WHQ, identify anomalies in the data, and evaluate the extent to which the WHQ items are measuring the latent trait of wound infection^38,39^. Individual items were assessed for excessive misfit (that is, not measuring the trait in question) and response dependency (where items are related by more than just the underlying trait). Additionally, appropriate use of item response categories was checked using category probability curves and threshold mapping. Where probability curves were disordered, response categories were rescored and item fit was then re-examined. Where residual correlations between items were high, subtesting was carried out with re-evaluation of item and model fit. Differential item functioning (DIF) was examined for each item by country, language, and patient home location (urban/rural). Exploration of DIF was undertaken only where a subgroup included at least 50 complete WHQ responses.

#### Triangulation

Qualitative and quantitative data were triangulated using data (between countries) and methodological (between qualitative interviews and psychometric analysis of quantitative data) triangulation, adopting a modified, exploratory, instrumental design model. Triangulation was performed item by item to enable a final version of the instrument in both source (English) and target languages to be finalized and consolidated^16,40–43^. Finally, there was a phase of proofreading, before completion of a final report of the adapted WHQ, and adoption of this version for further prospective validation. Data were also triangulated regarding measurement procedures to optimize future implementation of remote follow-up pathways.

### Community engagement and involvement

Patients and community members from LMICs were engaged in all phases of the design and delivery of this study. The interview topic guide was co-designed with input from a representative global surgery patient forum. Practicable methods for conducting interviews, and patient compensation for time in participation, were determined with the support of local community leaders. The Guidance for Reporting Involvement of Patients and the Public (GRIPP-2) short form was used to track and report the impact of CEI^44^.

## Results

### Qualitative phase

In total, 10 structured interviews and six focus groups were arranged with a total of 47 investigators across six countries. They included 34 surgeons, five anaesthetists, and eight research staff caring for patients in both urban and rural populations, and across a range of abdominal surgery disciplines. Interview duration ranged from 34 to 112 min, and focus groups lasted from 92 to 126 min. There was a median of 11 (range 6–16) participants involved in the focus groups. Interview and focus group data from site investigators confirmed that the assumption of a universalist approach to SSI was acceptable, and that symptomology and treatment paradigms were shared across settings. No divergence from this was identified during thematic analysis. This was also explored with the CEI partners; together, they confirmed content validity across settings. No new domains or concepts related to symptoms or treatment of SSI arose, suggesting content validity across contexts. A summary of qualitative data are presented for symptom items in *Table S1* and treatment items in *Table S2*. Themes emerged relating to comprehension, response mapping, retrieval, judgement, and novel cross-cultural insights.

Translation was successfully completed in five target languages after the qualitative phase: French (Benin), Hindi (India), Kinyarwanda (Rwanda), Punjabi (India), and Tamil (India). For some potential languages of delivery, there was no written version of the dialect (for example, Goun in Benin, Fante in Ghana), and, on rare occasions, patients would travel a very long distance for treatment and spoke a language that was uncommon to the local area (for example, Malayam in Northern India). Here, the questionnaire was translated ad hoc from English (source language) by the assessor in the cohort study.

### Quantitative phase

An attempt was made to contact 655 patients in the cohort study across five countries, of whom five had died by 30 days (15 missing status). Of the 635 confirmed to be alive, 537 (84.5 per cent) were contactable for WHQ completion. Features of included patients are summarized in *Table 2*.

**Table 2:** Patient characteristics (quantitative phase)

	Ghana	India	Benin	Mexico	Nigeria	Total
**Timing of WHQ**						
ȃPer protocol	224 ( 99.1)	3 ( 3.8)	100 (100)	12 ( 10.1)	13 (100)	352 ( 65.5)
ȃOutside of protocol	1 ( 0.4)	76 ( 96.2)	0 (0)	107 ( 89.9)	0 (0)	184 ( 34.3)
ȃMissing	1 ( 0.4)	0 (0)	0 (0)	0 (0)	0 (0)	1 ( 0.2)
**Age (years)**						
ȃ<18	33 ( 14.6)	0 (0)	0 (0)	2 ( 1.7)	3 ( 23.1)	38 ( 7.1)
ȃ18–39	115 ( 50.9)	65 ( 82.3)	82 ( 82.0)	79 ( 66.4)	4 ( 30.8)	345 ( 64.2)
ȃ40–59	51 ( 22.6)	10 ( 12.7)	13 ( 13.0)	27 ( 22.7)	4 ( 30.8)	105 ( 19.6)
ȃ60–79	24 ( 10.6)	4 ( 5.1)	5 ( 5.0)	9 ( 7.6)	2 ( 15.4)	44 ( 8.2)
ȃ≥80	3 ( 1.3)	0 (0)	0 (0)	2 ( 1.7)	0 (0)	5 ( 0.9)
**Sex**						
ȃM	142 ( 62.8)	7 ( 8.9)	55 ( 55.0)	16 ( 13.4)	7 ( 53.8)	227 ( 42.3)
ȃF	84 ( 37.2)	72 ( 91.1)	45 ( 45.0)	103 ( 86.6)	6 ( 46.2)	310 ( 57.7)
**Home location**						
ȃUrban	137 ( 60.6)	53 ( 67.1)	92 ( 92.0)	89 ( 74.8)	11 ( 84.6)	382 ( 71.1)
ȃRural	89 ( 39.4)	25 ( 31.6)	8 ( 8.0)	30 ( 25.2)	2 ( 15.4)	154 ( 28.7)
ȃMissing	0 (0)	1 ( 1.3)	0 (0)	0 (0)	0 (0)	1 ( 0.2)
**Level of education**						
ȃBelow high school level	157 ( 69.5)	32 ( 41.6)	29 ( 29.0)	23 ( 19.3)	6 ( 46.2)	247 ( 46.2)
ȃHigh school or above	69 ( 30.5)	45 ( 58.4)	71 ( 71.0)	96 ( 80.7)	7 ( 53.8)	288 ( 53.8)
**Known diabetes**						
ȃYes	4 ( 1.8)	3 ( 3.8)	1 ( 1.0)	9 ( 7.6)	0 (0)	17 ( 3.2)
ȃNo	222 ( 98.2)	76 ( 96.2)	99 ( 99.0)	110 ( 92.4)	13 (100)	520 ( 96.8)
**HIV status**						
ȃKnown negative	17 ( 7.5)	78 ( 98.7)	4 ( 4.0)	36 ( 30.3)	6 ( 46.2)	141 ( 26.3)
ȃKnown positive	1 ( 0.4)	0 (0)	0 (0)	2 ( 1.7)	0 (0)	3 ( 0.6)
ȃNot known	208 ( 92.0)	1 ( 1.3)	96 ( 96.0)	81 ( 68.1)	7 ( 53.8)	393 ( 73.2)
**Smoking status**						
ȃNever smoked	218 ( 96.5)	78 ( 98.7)	97 ( 97.0)	107 ( 89.9)	12 ( 92.3)	512 ( 95.3)
ȃEx-smoker	5 ( 2.2)	1 ( 1.3)	0 (0)	10 ( 8.4)	0 (0)	16 ( 3.0)
ȃCurrent smoker	3 ( 1.3)	0 (0)	3 ( 3.0)	2 ( 1.7)	1 ( 7.7)	9 ( 1.7)
**Urgency of surgery**						
ȃElective (planned)	20 ( 8.8)	24 ( 30.4)	0 (0)	94 ( 79.0)	2 ( 15.4)	140 ( 26.1)
ȃEmergency (unplanned)	206 ( 91.2)	55 ( 69.6)	100 (100)	25 ( 21.0)	11 ( 84.6)	397 ( 73.9)
**Indication**						
ȃMalignant disease	11 ( 4.9)	7 ( 8.9)	2 ( 2.0)	3 ( 2.5)	2 ( 15.4)	25 ( 4.7)
ȃBenign disease	201 ( 88.9)	9 ( 11.4)	97 ( 97.0)	64 ( 53.8)	10 ( 76.9)	381 ( 70.9)
ȃTrauma	9 ( 4.0)	0 (0)	1 ( 1.0)	0 (0)	0 (0)	10 ( 1.9)
ȃObstetric	5 ( 2.2)	63 ( 79.7)	0 (0)	52 ( 43.7)	1 ( 7.7)	121 ( 22.5)
**Operation site**						
ȃForegut	73 ( 32.3)	2 ( 2.5)	8 ( 8.0)	31 ( 26.1)	2 ( 15.4)	116 ( 21.6)
ȃHindgut	25 ( 11.1)	8 ( 10.1)	2 ( 2.0)	6 ( 5.0)	2 ( 15.4)	43 ( 8.0)
ȃAppendix	75 ( 33.2)	0 (0)	85 ( 85.0)	9 ( 7.6)	5 ( 38.5)	174 ( 32.4)
ȃUrogenital	6 ( 2.7)	65 ( 82.3)	0 (0)	67 ( 56.3)	1 ( 7.7)	139 ( 25.9)
ȃOther	47 ( 20.8)	4 ( 5.1)	5 ( 5.0)	6 ( 5.0)	3 ( 23.1)	65 ( 12.1)
**ASA grade**						
ȃI	144 ( 63.7)	23 ( 29.1)	77 ( 77.0)	28 ( 23.5)	2 ( 15.4)	274 ( 51.0)
ȃII	60 ( 26.5)	51 ( 64.6)	21 ( 21.0)	85 ( 71.4)	4 ( 30.8)	221 ( 41.2)
ȃIII	21 ( 9.3)	3 ( 3.8)	2 ( 2.0)	6 ( 5.0)	6 ( 46.2)	38 ( 7.1)
ȃIV–V	0 (0)	2 ( 2.5)	0 (0)	0 (0)	1 ( 7.7)	3 ( 0.6)
ȃMissing	1 ( 0.4)	0 (0)	0 (0)	0 (0)	0 (0)	1 ( 0.2)
**WHO Checklist**						
ȃYes	214 ( 94.7)	79 (100)	99 ( 99.0)	116 ( 97.5)	10 ( 76.9)	518 ( 96.5)
ȃNo	12 ( 5.3)	0 (0)	1 ( 1.0)	3 ( 2.5)	3 ( 23.1)	19 ( 3.5)
**Operation grade**						
ȃIntermediate/minor	79 ( 35.7)	0 (0)	85 ( 85.0)	9 ( 7.6)	5 ( 38.5)	178 ( 33.6)
ȃMajor	142 ( 64.3)	78 (100)	15 ( 15.0)	109 ( 92.4)	8 ( 61.5)	352 ( 66.4)
**Contamination level**						
ȃClean/clean-contaminated	44 ( 19.5)	73 ( 92.4)	14 ( 14.0)	110 ( 92.4)	2 ( 15.4)	243 ( 45.3)
ȃContaminated	106 ( 46.9)	6 ( 7.6)	38 ( 38.0)	7 ( 5.9)	5 ( 38.5)	162 ( 30.2)
ȃDirty	74 ( 32.7)	0 (0)	48 ( 48.0)	1 ( 0.8)	6 ( 46.2)	129 ( 24.0)
ȃMissing	2 ( 0.9)	0 (0)	0 (0)	1 ( 0.8)	0 (0)	3 ( 0.6)
**Surgical approach**						
ȃOpen midline	175 ( 77.4)	11 ( 13.9)	33 ( 33.0)	28 ( 23.5)	7 ( 53.8)	254 ( 47.3)
ȃOpen non-midline	50 ( 22.1)	65 ( 82.3)	67 ( 67.0)	89 ( 74.8)	6 ( 46.2)	277 ( 51.6)
ȃLaparoscopic attempted	0 (0)	3 ( 3.8)	0 (0)	2 ( 1.7)	0 (0)	5 ( 0.9)
ȃMissing	1 ( 0.4)	0 (0)	0 (0)	0 (0)	0 (0)	1 ( 0.2)
**Stoma formation**						
ȃYes	9 ( 4.0)	5 ( 6.3)	0 (0)	2 ( 1.7)	0 (0)	16 ( 3.0)
ȃNo	215 ( 95.1)	74 ( 93.7)	100 (100)	117 ( 98.3)	13 (100)	519 ( 96.6)
ȃMissing	2 ( 0.9)	0 (0)	0 (0)	0 (0)	0 (0)	2 ( 0.4)

Values are *n* (%). WHQ, Wound Healing Questionnaire; HIV, human immunodeficiency virus.

### Unidimensionality of scale

The exploratory Rasch model was fitted using these data from 537 patients (369 excluding extremes) across five class intervals (*Table S3*). Both analysis of principal components between positively and negatively loading items (1.9 per cent, *n* = 10 independent *t* tests less than 5 per cent) and symptom and pathway items (0.6 per cent, *n* = 8) suggested unidimensionality of the WHQ instrument in detection of SSI.

### Model fit and targeting

**Fig. 2:**
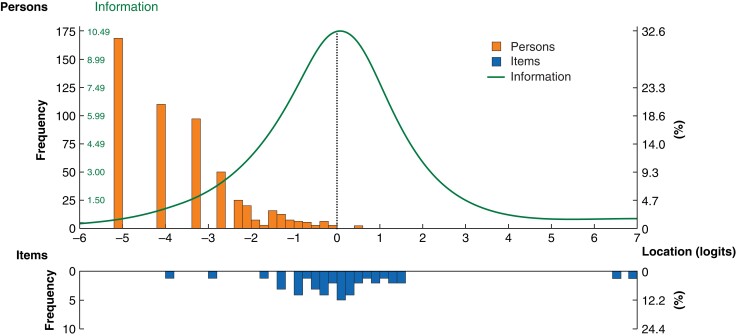
Person–threshold distribution map of the Wound Healing Questionnaire

**Fig. 3:**
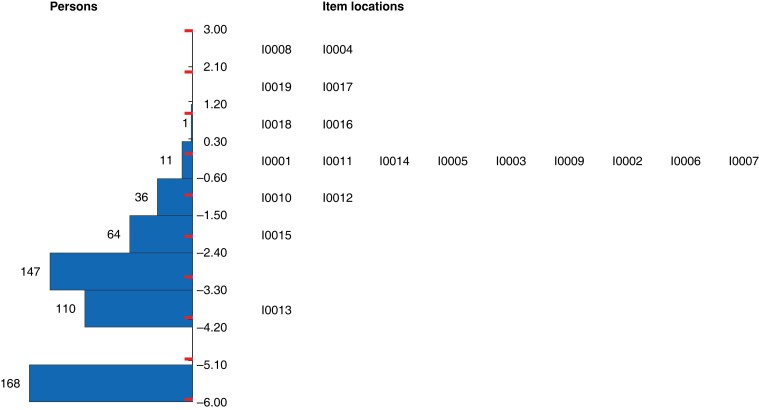
Item location map for the adapted Wound Healing Questionnaire

Overall, the model did not fit well, with a high probability of item–trait interaction (χ^2^ 209.2, 76 d.f., *P* < 0.001) and a poor person separation index (0.48, low power of analysis). Conversely, Cronbach’s α (with missing data excluded) demonstrated acceptable internal consistency, with a value of 0.86. There was a strong positive skew of person location values, with a mean(s.d.) person location of −2.91(1.05), demonstrating some mistargeting of the WHQ, as may be expected in a diagnostic or screening tool (*Fig. 2*). The item location map reflected clinical severity, with 168 of 537 participants (31.3 per cent) at the floor of the scale (no signs or symptoms of SSI), and item locations reflecting degrees of infection at the ceiling (*Fig. 3*).

Grouping set to interval length of 0.20, making 66 groups. There were 537 patients in total.

I0001–I0019, items 1–19. Item 13 (dressing) was the lowest item, indicating that many of the participants would have scored on this item. Items such as 8 (local swelling), 4 (blood-stained fluid), 19 (reoperation), and 17 (wound debridement) were at the ceiling and participants were more likely to affirm these items if they had a more severe infection. Y-axis represents the item location along the Guttman-style Rasch scale. The bars are the number of persons (patients) as labelled. The red bars are integer values along the scale (ie. 3, 2, 1, 0, −1, −2, −3) etc.

### Individual item fit and dependency

Five items (5, 9, 14, 15, 16) displayed significant misfit to the model (mean(s.d.) item fit residual −1.61(1.75)) (*Table S4*), but the person fit was acceptable (mean(s.d.) person fit residual −0.52(0.69)). Examination of individual-person fit did not reveal any significant misfit (s.d. of fit residual greater than +2.5 or less than −2.5). There was a high degree of correlation and dependence between items with local dependency in 11 item pairs (*Table S5*).

### Differential item functioning

There was significant evidence of uniform differential item functioning (DIF) by country in items 1, 3, 5, 8, 10, and 13, and non-uniform DIF by country in items 4, 10, 13, 16, 17, and 19 (*Table S6)*. There was no significant DIF observed by patient home location (*Table S7)*.

### Triangulation

**Fig. 4:**
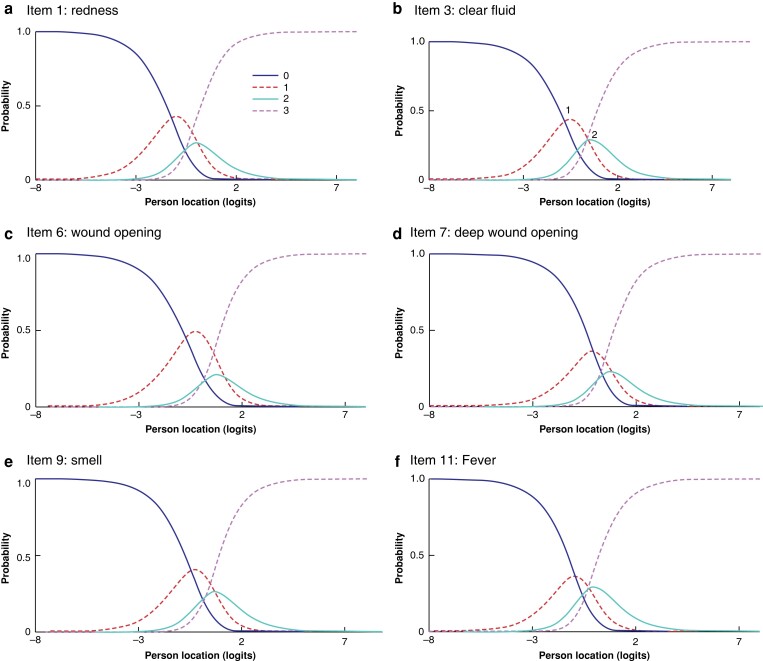
Category probability curves for items with an overlapping response threshold

**Fig. 5:**
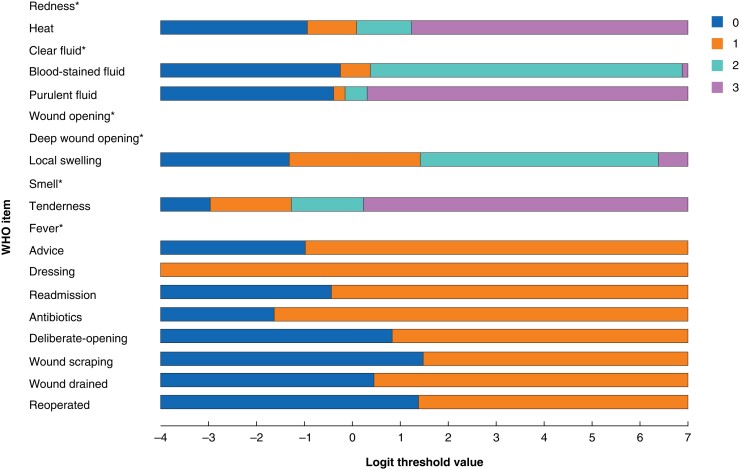
Threshold map for Wound Healing Questionnaire

Triangulation of qualitative and quantitative data was performed item by item for the 11 symptom items (10 items and 1 subitem) and eight pathway items (*Appendix S10*). Where deductive cognitive themes or inductive cross-cultural themes arose, they were explored against individual item fit, dependency, and DIF in the Rasch model (*Figs S1–S4*). Recommendations were made for cross-cultural adaptation for WHQ items 1 (redness), 3 (clear fluid), 7 (deep wound opening), 10 (pain), 11 (fever), 15 (antibiotics), 16 (debridement), 18 (drainage), and 19 (reoperation). When triangulating disordered threshold probabilities (*Figs 4* and *5*) with corroborating or conflicting qualitative data, a recommendation was made to move to three item response categories (1, not at all; 2, a little; 3, a lot) for symptom items 1 to 10, and to two categories (0, no; 1, yes) for item 11 (fever). A summary of recommendations is displayed in *Table 3*, and the final adapted questionnaire in *Appendix S11*. Translated versions of the adapted WHQ are provided in *Appendix S12*.

**a** Item 1 (redness), **b** item 3 (clear fluid), **c** item 6 (wound opening), **d** item 7 (deep wound opening), **e** item 9 (smell), and **f** item 11 (fever). **a** Location –0.540, spread 0.186, FitRes –0.459, χ^2^[Pr] 0.190, F[Pr] 0.277; **b** location –0.012, spread 0.297, FitRes –0.402, χ^2^[Pr] 0.707, F[Pr] 0.818; **c** location 0.166, spread 0.195, FitRes –1.769, χ^2^[Pr] 0.117, F[Pr] 0.198; **d** location 0.294, spread 0.050, FitRes 0.407, χ^2^[Pr] 0.222, F[Pr] 0.312; **e** location 0.115, spread 0.171, FitRes –3.519, χ^2^[Pr] 0.005, F[Pr] 0.000; **f** location –0.503, spread 0.145, FitRes 0.172, χ^2^[Pr] 0.000, F[Pr] 0.000. FitRes, Fit residual value, χ2[Pr], Chi-squared test statistic; F[Pr], F-statistic.

The higher the ‘threshold’ of transition in each item from a low scoring value to a higher scoring value, the more indicative (‘difficult’ in Rasch terminology) that item was in detecting the ‘latent trait’ (surgical-site-infection, SSI) in the Rasch model. For example, item 19 (reoperated) and item 17 (wound scraping) had the highest threshold so were most likely to indicate SSI, whereas item 10 (tenderness) and item 13 (dressing) had the lowest threshold so were least likely to indicate SSI (in isolation). *Items with disordered thresholds (overlapping category probability curves seen in *Fig. 4*).

**Table 3:** Summary of recommendations for adaptation of Wound Healing Questionnaire (English language)

Item	Original item	Original response categories	Adapted item	Adapted response categories
1	Was there redness spreading away from the wound?	1, not at all; 2, a little; 3, quite a bit; 4, a lot	Was there redness (or shining of the skin) spreading away from the wound?	1, not at all; 2, little; 3, a lot
2	Was the area around the wound warmer than the surrounding skin?	1, not at all; 2, a little; 3, quite a bit; 4, a lot	–	1, not at all; 2, little; 3, a lot
3	Has any part of the wound leaked clear fluid?	1, not at all; 2, a little; 3, quite a bit; 4, a lot	Has any part of the wound leaked thin clear fluid?	1, not at all; 2, little; 3, a lot
4	Has any part of the wound leaked blood-stained fluid?	1, not at all; 2, a little; 3, quite a bit; 4, a lot	–	1, not at all; 2, little; 3, a lot
5	Has any part of the wound leaked thick and yellow or green fluid?	1, not at all; 2, a little; 3, quite a bit; 4, a lot	–	1, not at all; 2, little; 3, a lot
6	Have the edges of any part of the wound separated or gaped open of their accord?	1, not at all; 2, a little; 3, quite a bit; 4, a lot	–	1, not at all; 2, little; 3, a lot
7	If the wound edges opened, did the deeper tissue also separate?	1, not at all; 2, a little; 3, quite a bit; 4, a lot	If the wound edges opened, did the flesh beneath the skin or the inside sutures also separate?	1, not at all; 2, little; 3, a lot
8	Has the area around the wound become swollen?	1, not at all; 2, a little; 3, quite a bit; 4, a lot	–	1, not at all; 2, little; 3, a lot
9	Has the wound been smelly?	1, not at all; 2, a little; 3, quite a bit; 4, a lot	–	1, not at all; 2, little; 3, a lot
10	Has the wound been painful to touch?	1, not at all; 2, a little; 3, quite a bit; 4, a lot	Has the wound been painful to touch?	1, not at all; 2, little; 3, a lot
11	Have you had, or felt like you have had, a raised temperature or fever (>38°C)?	1, not at all; 2, a little; 3, quite a bit; 4, a lot	Have you had, or felt like you have had, a raised temperature or fever?	1, no; 2, yes
12	Have you sought advice because of a problem with your wound, other than at a planned follow-up appointment?	1, no; 2, yes	–	–
13	Has anything been put on the skin to cover the wound? (dressing)	1, no; 2, yes	–	–
14	Have you been back into hospital for a problem with your wound?	1, no; 2, yes	–	–
15	Have you been given antibiotics for a problem with your wound?	1, no; 2, yes	Have you been given medicines (antibiotics) for a problem with your wound?	–
16	Have the edges of your wound been deliberately separated by a doctor or nurse?	1, no; 2, yes	Have the edges of your wound been separated by a doctor or nurse?	–
17	Has your wound been scraped or cut to remove any unwanted tissue?	1, no; 2, yes	–	–
18	Has your wound been drained (drainage of pus or an abscess)?	11, no; 2, yes	Has thick, yellow, or green fluid (pus) been drained from your wound by a doctor or nurse (abscess)?	–
19	Have you had an operation under general anaesthetic for treatment of a problem with your wound?	1, no; 2, yes	Have you had to go back to the operating room for treatment of a problem with your wound?	–

### Measurement procedures

A summary of measurement procedures is shown in *Table 4*. Despite concerns with mobile phone connectivity in qualitative data, telephone WHQ completion was feasible (537 of 635, 84.5 per cent) with high data completeness (99.0 per cent instruments complete overall, range by item 99.1–100 per cent).

‘People were very impressed that I was calling them and still following up on the surgeries and were willing to talk very happily.’ (Research nurse, Focus group IN002F, India)

**Table 4:** Measurement processes (quantitative phase)

	Ghana	India	Benin	Mexico	Nigeria	Total
**Language of delivery (translated WHQ)**						
ȃEnglish	32 ( 14.2)	2 ( 2.5)	0 (0)	0 (0)	9 ( 69.2)	43 ( 8.0)
ȃFrench	0 (0)	0 (0)	88 ( 88.0)	0 (0)	0 (0)	88 ( 16.4)
ȃHindi	0 (0)	52 ( 65.8)	0 (0)	0 (0)	0 (0)	52 ( 9.7)
ȃPunjabi	0 (0)	20 ( 25.3)	0 (0)	0 (0)	0 (0)	20 ( 3.7)
ȃSpanish	0 (0)	0 (0)	0 (0)	119 (100)	0 (0)	119 ( 22.2)
ȃTamil	0 (0)	3 ( 3.8)	0 (0)	0 (0)	0 (0)	3 ( 0.6)
**Language of delivery (*ad hoc* translation)**						
ȃDagbani	38 ( 16.8)	0 (0)	0 (0)	0 (0)	0 (0)	38 ( 7.1)
ȃFante	8 ( 3.5)	0 (0)	0 (0)	0 (0)	0 (0)	8 ( 1.5)
ȃFon	0 (0)	0 (0)	6 ( 6.0)	0 (0)	0 (0)	6 ( 1.1)
ȃGoun	0 (0)	0 (0)	6 ( 6.0)	0 (0)	0 (0)	6 ( 1.1)
ȃMalayalam	0 (0)	2 ( 2.5)	0 (0)	0 (0)	0 (0)	2 ( 0.4)
ȃTwi	148 ( 65.5)	0 (0)	0 (0)	0 (0)	0 (0)	148 ( 27.6)
ȃYoruba	0 (0)	0 (0)	0 (0)	0 (0)	4 ( 30.8)	4 ( 0.7)
**Telephone owner**						
ȃPatient themselves	142 ( 62.8)	23 ( 29.1)	84 ( 84.0)	85 ( 71.4)	10 ( 76.9)	344 ( 64.1)
ȃHealthcare worker	0 (0)	1 ( 1.3)	0 (0)	0 (0)	0 (0)	1 ( 0.2)
ȃFriend or relative	83 ( 36.7)	54 ( 68.4)	16 ( 16.0)	34 ( 28.6)	2 ( 15.4)	189 ( 35.2)
ȃOther	1 ( 0.4)	1 ( 1.3)	0 (0)	0 (0)	1 ( 7.7)	3 ( 0.6)
**Telephone type**						
ȃLandline	0 (0)	1 ( 1.3)	0 (0)	2 ( 1.7)	0 (0)	3 ( 0.6)
ȃMobile (with camera)	118 ( 52.2)	70 ( 88.6)	77 ( 77.0)	104 ( 87.4)	11 ( 84.6)	380 ( 70.8)
ȃMobile (without camera)	108 ( 47.8)	8 ( 10.1)	23 ( 23.0)	13 ( 10.9)	2 ( 15.4)	154 ( 28.7)
**Questionnaire administrator**						
ȃConsultant (doctor)	0 (0)	0 (0)	0 (0)	7 ( 5.9)	2 ( 15.4)	9 ( 1.7)
ȃOther doctor	132 ( 58.4)	0 (0)	100 (100)	75 ( 63.0)	0 (0)	307 ( 57.2)
ȃResearch nurse	65 ( 28.8)	76 ( 96.2)	0 (0)	0 (0)	1 ( 7.7)	142 ( 26.4)
ȃOther	28 ( 12.4)	3 ( 3.8)	0 (0)	37 ( 31.1)	10 ( 76.9)	78 ( 14.5)
ȃMissing	1 ( 0.4)	0 (0)	0 (0)	0 (0)	0 (0)	1 ( 0.2)

Values are *n* (%). WHQ, Wound Healing Questionnaire.

In total, 533 of 537 patients (99.2 per cent) reported the telephone WHQ pathway to be very satisfactory or satisfactory:

‘Early feedback that the questionnaire is highly acceptable to patients. Patients say they are receiving a ‘VIP’ treatment.’ (Junior doctor, Focus group GH001F, Ghana)

Often the telephone owner was a friend or relative (who was then able to connect the researcher directly to the patient) rather than the patient themselves (189 of 537, 35.2 per cent), and commonly this was a mobile phone (534 of 537, 99.5 per cent). In total, 154 of 537 (28.7 per cent) had a mobile phone with video capability. Feedback from CEI partners alongside interview data supported optimization of the telephone follow-up pathway for future implementation; this is presented in a toolkit available in *Appendix S13*.

## Discussion

Pathways for remote assessment of common complications after surgery in low-resource settings are essential in improving the safety and resilience of surgical care systems. This mixed-methods study made recommendations for cross-cultural and cross-language adaptation of the WHQ for use in LMICs, and improved its relevance across cultures and for patients with lower levels of health literacy. Conceptual equivalence, and content and construct validity was confirmed across languages using qualitative and translation methods. Unidimensionality, measurement properties, and use of the total WHQ score were seen to be valid within the Rasch framework, although the overall power of fit was low. The telephone pathway was demonstrated to be feasible and highly acceptable. Working with CEI partners, recommendations were made for optimization of telephone follow-up in research and postoperative surveillance programmes. This study provides a large, international, high-quality proof of concept for rapid adaptation and implementation of patient-reported measures in emerging global health arenas such as surgery.

The use of mixed methods here added strength and depth. The qualitative data were used primarily to inform cross-cultural adaptation ahead of translation. Although this was based on cognitive theory, data were collected indirectly about patient experience from frontline clinicians involved in wound assessment. The Rasch analysis supplemented this, and allowed patient-level data to enrich and inform final recommendations for adaptation. In a majority of instances, the qualitative and quantitative data were supportive of one another, demonstrating coherence during triangulation. Where conflict arose, qualitative findings were softened and/or caveated (that is, changes were recommended where there was coherence on triangulation, and further exploration recommended where there was conflict between the qualitative and quantitative data).

Rasch analysis is an established method for instrument development and cross-cultural refinement^39,45,46^. Here, its principal value was in confirming the validity of use of the total WHQ score as an ordinal scale and in enhancing understanding of the response structure and local dependency. Properties of the WHQ, however, make it a rather unusual application of the Rasch model. First, it is principally a diagnostic tool for SSI rather than an interval-level tool measuring a spectrum of severity of a latent trial. This was best seen in mistargeting of the WHQ to the study population, with many patients at the ‘floor’ adding low information value to the model, as would be expected in a screening tool (where many patients are asymptomatic). This reduced the overall power of fit as many participants contributed little information about item locations. Second, as expected in a diagnostic test, many items had high levels of local dependency, which may have contributed to the overall model misfit. Third, several items misfit the Rasch model and the person separation index was poor, with a conversely high Cronbach’s α value. Again, this is highly likely to be due to the extreme ‘floor’ of respondents in the setting of a diagnostic tool. It was not the overall aim to fit this diagnostic tool closely to the Rasch model, and it would not be required to be valid for use if it demonstrated a satisfactory psychometric structure, unidimensionality, and sufficient sensitivity and specificity upon clinical application. This highlights the importance of further work to validate the tool externally in a diagnostic test accuracy study.

Exploring complex relationships between items and optimizing the measurement properties using subtesting and adjusting for DIF was not the aim here, but warrants further investigation. It is feasible that the instrument could be simplified, or its diagnostic accuracy could be improved using Rasch by better accounting for differences in the symptomology and health-seeking behaviours of patients with SSI across countries. DIF by country observed for several items here supports methods to ensure balance in randomized trials, such as stratification or minimization of randomization by country.

This study has several limitations. Owing to safety and ethical concerns during the SARS-CoV-2 pandemic, cognitive interviewing could not be undertaken directly with patients. Instead, aggregate perspectives of frontline clinicians involved in the care of surgical patients were explored. This meant that the data represented clinicians’ impressions of patients’ responses, and challenges in retrieval and judgement, rather than direct exploration with patients in typical cognitive interviewing^29^. Sampling of researchers directly involved in the same portfolio of trials was a pragmatic decision, but may have reduced the transferability of themes across other hospital types (for example, remote rural hospitals), resource settings (such as hospitals with less research infrastructure) or differing populations (for example, less literate populations, with poorer access to healthcare). Thematic saturation overall was aimed for when ending recruitment to the qualitative phase, but this is unlikely to have been reached at an individual-country level^47^. It is, therefore, possible that important insights were missed during adaptation, although recommendations were strengthened by triangulation with quantitative data to reduce over-reliance on qualitative data alone^40^. Second, related to analysis, as the WHQ did not meet all the Rasch assumptions for model fit, a logit-adjusted scale was not developed. Further development could improve the measurement properties of the questionnaire to allow direct patient-to-patient comparisons in future research. Complex patterns of DIF in measurement that could lead to differences in point score equivalence across different patients with differing characteristics when applied clinically were not taken into account. Finally, related to interpretation, the most important metric of clinical utility in a screening tool such as this would be diagnostic test accuracy. A formal external validation study comparing the WHQ to a standard reference test for SSI is now required^20,48^. A choice of cut-off score for the adapted WHQ is likely to favour sensitivity to triage all patients with a likelihood of SSI to seek medical care.

The use of patient-reported outcome measures (PROMs) in low-income settings is complex; many instruments have not yet undergone cross-cultural and cross-language adaptation, and there is uncertainty about the feasibility of remote, digital methods. Although examples exist from established global health fields, such cardiovascular disease, few studies in global surgery have adopted PROMs to date^49–51^. Health technology assessments thus neglect important insights into quality of recovery and health utility that could affect policy decisions^52^. This study provides a proof of concept for rapid, pragmatic adaptation of instruments in the surgical setting that can be used across other measures and emerging contexts. Developing culturally attuned, remote follow-up pathways is particularly important during pandemic recovery in building resilience in resource-poor health systems^53,54^. The co-produced pathway for telephone follow-up in LMICs described here is ready for wider adoption. Recommendations from this mixed-methods study can now to be used for further exploration of the diagnostic accuracy of the adapted WHQ in low-resource contexts.

## Collaborators

NIHR Global Health Research Unit on Global Surgery: James Glasbey, Adesoji Ademuyiwa, Alisha Bhatt, Bruce Biccard, Jane Blazeby, Peter Brocklehurst, Sohini Chakrabortee, JC Allen Ingabire, Francis Moïse Dossou, Irani Durán, Rohini Dutta, Dhruva Ghosh, Frank Gyamfi, Parvez Haque, Pollyanna Hardy, Mike Horton, Gabriella Hyman, Ritu Jain, Oluwaseun Ladipo-Ajayi, Ismail Lawani, Souliath Lawani, Mwayi Kachapila, Rachel Lillywhite, Rhiannon Macefield, Laura Magill, Janet Martin, Jonathan Mathers, Kenneth McLean, Punam Mistry, Rohin Mittal, Mark Monahan, Rachel Moore, Dion Morton, Moyo Ojo, Faustin Ntirenganya, Emmanuel Ofori, Rupert Pearse, Alberto Peón, Thomas Pinkney, Antonio Ramos de la Medina, Tubasiime Ronald, David Roman, Emmy Runingamugabo, Alice Sitch, Anita Slade, Donna Smith, Stephen Tabiri, Aneel Bhangu, James Glasbey, Anita Slade, Mike Horton, Rhiannon Macefield, Aneel Bhangu, Pollyanna Hardy, Adesoji O Ademuyiwa, Lawani Ismail, Dhruva Ghosh, Antonio Ramos de la Medina, Rachel Moore, Faustin Ntirenganya, Stephen Tabiri, Emmy Runingamugabo, Simin Patrawala, Angela Prah, Christian Oko, Karolin Kroese, Ismaïl Lawani, Francis Moïse Dossou, Corinne Dzemta, Covalic Melic Bokossa Kandokponou, Souliath Lawani, Hulrich Behanzin, Cyrile Kpangon, Bernard Appiah Ofori, Stephen Tabiri, Abdul-Hafiz Saba, Gbana Limann, Daniel Kwesi Acquah, Shamudeen Mohammed Alhassan, Sheriff Mohammed, Owusu Abem Emmanuel, Yakubu Musah, Yenli Edwin, Sheba Kunfah, Yakubu Mustapha, Abantanga Atindaana Francis, Emmanuel Ayingayure, Gbana Limann, Forster Amponsah-Manu, Eric Agyemang, Vera Agyekum, Esther Adjei-Acquah, Emmanuel Yaw Twerefour, Barbra Koomson, Ruby Acheampong Boateng, Ato Oppong Acquah, Richard Ofosu-Akromah, Leslie Issa Adam-Zakariah, Nii Armah Adu-Aryee, Theodore Wordui, Coomson Christian Larbi, Akosa Appiah Enoch, Mensah Elijah, Kyeremeh Christian, Addo Gyambibi Kwame, Boakye Percy, Kontor Effah Bismark, Gyamfi Brian, Manu Ruth, Romeo Hussey, Samuel Dadzie, Akosua Dwamena Appiah, Grace Yeboah, Cynthia Yeboah, James Amoako, Regina Acquah, Naa Anyekaa Sowah, Atta Kusiwaa, Esther Asabre, Cletus Ballu, Charles Gyamfi Barimah, Frank Owusu, Clement Sie-Broni, Vivian Adobea, Prince Yeboah Owusu, Marshall Zume, Abdul-Hamid Labaran, Raphael Adu-Brobbey, Martin Tangnaa Morna, Samuel A. Debrah, Patrick Opoku Manu Maison, Michael Nortey, Donald Enti, Mabel Pokuah Amoako-Boateng, Anthony Baffour Appiah, Emmanuel Owusu Ofori, Richard Kpankpari, Benedict Boakye, Elizabert Mercy Quartson, Patience Koggoh, Anita Eseenam Agbeko, Frank Enoch Gyamfi, Joshua Arthur, Joseph Yorke, Christian Kofi Gyasi-Sarpong, Charles Dally, Agbenya Kobla Lovi, Michael Amoah, Boateng Nimako, Robert Sagoe, Anthony Davor, Fareeda Galley, Michael Adinku, Jonathan Boakye-Yiadom, Jane Acquaye, Juliana Appiah, Dorcas Otuo Acheampong, Iddrisu Haruna, Edward Amoah Boateng, Emmanuel Kafui Ayodeji, Samuel Tuffuor, Naa Kwarley, Yaa Tufuor, Ramatu Darling Abdulai, Fred Dankwah, Ralph Armah, Doris Ofosuhene, Dorcas Osei-Poku, Arkorful Ebenezer Temitope, Delali Akosua Gakpetor, Victoria Sena Gawu, Christopher Asare, Enoch Tackie, James Ankomah, Isaac Omane Nyarko, Zelda Robertson, Serbeh Godwin, Appiah Anthony Boakye, Godfred Fosu, Frank Assah-Adjei, Parvez Haque, Ritu Jain, Alisha Bhatt, Jyoti Dhiman, Rohini Dutta, Dhruva Ghosh, Esther Daniel, Priyadarshini K, Latha Madankumar, Rohin Mittal, Ida Nagomy, Soosan Prasad, Arpit Jacob Mathew, Danita Prakash, Priya Jacob, Jeremiah *P* Anachy, Amy Mathew, Josy Thomas, Philip V Alexander, Pradeep Zechariah, Neerav D Aruldas, Asif Mehraj, Hafsa Imtiyaz Ahmed, Rauf A Wani, Fazl Q Parray, Nisar A Chowdri, Antonio Ramos De la Medina*, Laura Martinez Perez Maldonado, Diana S Gonzalez Vazquez, Iran I Durán Sánchez, Maria J Martínez Lara, Alejandra Nayen Sainz de la Fuente, Ana O Cortes Flores, Mariana E Barreto Gallo, Alejandro Gonzalez Ojeda, Monica E Jimenez Velasco, Luis Hernández Miguelena, Reyes J Cervantes Ortiz, Gonzalo I Hernandez Gonzalez, Marco Hurtado Romero, Rosa I Hernandez Krauss, Luis A Dominguez Sansores, Alejandro Cuevas Avendaño, Celina Cuellar Aguirre, Isaac Baltazar Gomez, Hector Ortiz Mejia, Alejandro González Ojeda, Oscar E Olvera Flores, Erick A González García de Rojas, Kevin J Pintor Belmontes, Francisco J Barbosa Camacho, Aldo Bernal Hernández, Laura Reyes Aguirre, Rubén E Morán Galaviz, Clotilde Fuentes Orozco, Wenceslao G Ángeles Bueno, Fernando S Ramirez Marbello, Diego E Luna Acevedo, Michel Hernández Valadez, Ana L Bogurin Arellano, Luis R Ramírez-González, Bertha G Guzmán Ramírez, Eduardo Valtierra Robles, Ramona I Rojas García, José V Pérez Navarro, Edgar J Cortes Torres, David R Dominguez Solano, Alberto N Peón, Roque D Lincona Menindez, Rozana Reyes Gamez, Maria C Paz Muñoz, Orimisan Belie, Victoria Adeleye, Adesoji Ademuyiwa, Oluwafunmilayo Adeniyi, Opeyemi Akinajo, David Akinboyewa, Felix Alakaloko, Oluwole Atoyebi, Olanrewaju Balogun, Christopher Bode, Olumide Elebute, Francis Ezenwankwo, Adesiyakan Adedotun, George Ihediwa, Jubril Kuku, Oluwaseun Ladipo-Ajayi, Ayomide Makanjuola, Samuel Nwokocha, Olubunmi Ogein, Rufus Ojewola, Abraham Oladimeji, Thomas Olajide, Iyabo Alasi, Oluwaseun Oluseye, Justina Seyi-Olajide, Adaiah Soibi-Harry, Emmanuel Williams, Agbulu Moses Vincent, Nnamdi Jonathan Duru, Kenneth Uche Onyekachi, Christiana Ashley, Chinelo Victoria Mgbemena, Moyosoluwa Ojo, Olowu Oluyemisi, Iyabode Ikuewunmi, Adeoluwa Adebunmi, Edet Glory Bassey, Ephraim Okwudiri Ohazurike, Olayide Michael Amao, Osunwusi Benedetto Oluwaseun, Emily Doris, Olutola Stephen, Christianah Gbenga-Oke, Olawunmi Olayioye, Olowu Oluyemisi, Kayode Oluremi, Esther Abunimye, Christianah Oyegbola, Olayade Kayode, Adeola Ayoola Orowale, Omolara M Williams, Olufunmilade A Omisanjo, Omolara M Faboya, Zainab O Imam, Olabode A Oshodi, Yusuf A Oshodi, Ayokunle A Ogunyemi, Olalekan T Ajai, Francisca C Nwaenyi, Adewale O Adisa, Adewale A Aderounmu, Funmilola O Wuraola, Oludayo Sowande, Lukman Olajide Abdur-Rahman, Jibril Oyekunle Bello, Hadijat Olaide Raji, Nurudeen Abiola Adeleke, Saheed Abolade Lawal, Rafiat Tinuola Afolabi, Abdulwahab Lawal, Okechukwu Hyginus Ekwunife, Ochomma Amobi Egwuonwu, Chisom Faith Uche, Abubakar Bala AB Muhammad, Saminu S Muhammad, Idris Usman IU Takai, Mohammed AS Aliyu Salele, Onyekachi G Ukata, Mahmoud Kawu MK Magashi, Lawal Barau LB Abdullahi, Bello Abodunde BA Muideen, Khadija A Ado, Lofty-John Chukwuemeka LJC Anyawu, Samson Olori*, Samuel A Sani, Olabisi O Osagie, Ndubuisi Mbajiekwe, Oseremen Aisuodionoe-Shadrach, Godwin O Akaba, Lazarus Ameh, Lazarus Ameh, Francis o Adebayo, Martins Uanikhoba, Felix O Ogbo, Nancy O Tabuanu, Taiwo A Lawal, Rukiyat A Abdus-Salam, Akinlabi E Ajao, Augustine O Takure, Omobolaji O Ayandipo, Hyginus O Ekwuazi, Olukayode Abayomi, Olatunji O Lawal, Solomon Olagunju, Kelvin I Egbuchulem, Sikiru Adekola Adebayo, Peter Elemile, Usang E Usang, Joseph E Udosen, Expo E Edet, Akan W Inyang, Edima M Olory, Gabriel U Udie, Godwin O Chiejina, Adams D Marwa, Faith J Iseh, Sunday A Ogbeche, Mary O Isa, Uchechukwu O Ezomike, Sebastian O Ekenze, Matthew I Eze, Emmanuel O Izuka, Jude K Ede, Vincent C Enemuo, Okezie M Mbadiwe, Ngozi G Mbah, Alphonsine Imanishimwe, Sosthene Habumuremyi, Faustin Ntirenganya, JC Allen Ingabire, Isaie Ncogoza, Emmanuel Munyaneza, Jean de Dieu Haragirimana, Christian Jean Urimubabo, Violette Mukanyange, Jeannette Nyirahabimana, Emmanuel Mutabazi, Christophe Mpirimbanyi, Olivier Mwenedata, Hope Lydia Maniraguha, Emelyne Izabiriza, Moses Dusabe, Job Zirikana, Francine Uwizeyimana, Josiane Mutuyimana, Elisee Rwagahirima, Alphonsine Imanishimwe, Ronald Tubasiime, Aphrodis Munyaneza, Sosthene Habumuremyi, Salathiel Kanyarukiko, Gibert Ndegamiye, Francine Mukaneza, Jean Claude Uwimana, Pierrine Nyirangeri, Deborah Mukantibaziyaremye, Aime Dieudonne Hirwa*, Salomee Mbonimpaye, Piolette Muroruhirwe, Christine Mukakomite, Elysee Kabanda, Rachel Moore, Ncamsile Anthea Nhlabathi, Maria Fourtounas, Mary Augusta Adams, Gabriella Hyman, Hlengiwe Samkelisiwe Nxumalo, Nnosa Sentholang, Mmule Evelyn Sethoana, Mpho Nosipho Mathe,

Zain Ally*, Margot Flint, Bruce Biccard, Adesoji O Ademuyiwa, Adewale O. Adisa, Aneel Bhangu, Peter Brocklehurst, Sohini Chakrabortee, Pollyanna Hardy, Ewen Harrison, JC Allen Ingabire, Parvez D Haque, Lawani Ismail, James Glasbey, Dhruva Ghosh, Frank Enoch Gyamfi, Elizabeth Li, Rachel Lillywhite, Antonio Ramos de la Medina, Rachel Moore, Laura Magill, Dion Morton, Dmitri Nepogodiev, Faustin Ntirenganya, Thomas Pinkney, Omar Omar, Joana Simoes, Donna Smith, Stephen Tabiri, Adesoji O Ademuyiwa, Lawani Ismail, Dhruva Ghosh, Antonio Ramos de la Medina, Rachel Moore, Faustin Ntirenganya, Stephen Tabiri, Adesoji Ademuyiwa, Aneel Bhangu, Felicity Brant, Peter Brocklehurst, Sohini Chakrabortee, Dhruva Ghosh, James Glasbey, Pollyanna Hardy, Ewen Harrison, Emily Heritage, Lawani Ismail, Karolin Kroese, Carmela Lapitan, Rachel Lillywhite, David Lissauer, Laura Magill, Antonio Ramos de la Medina, Punam Mistry, Mark Monahan, Rachel Moore, Dion Morton, Dmitri Nepogodiev, Faustin Ntirenganya, Omar Omar, Thomas Pinkney, Tracy Roberts, Donna Smith, Stephen Tabiri, Neil Winkles, Pollyanna Hardy, Omar Omar, Emmy Runigamugabo, Azmina Verjee, Pierre Sodonougbo, Pamphile Assouto, Michel Fiogbe, Houenoukpo Koco, Serge Metchinhoungbe, Hodonou Sogbo, Hulrich Behanzin, Djifid Morel Seto, Yannick Tandje, Sosthène Kangni, Cyrile Kpangon*, Marcelin Akpla, Hugues Herve Chobli, Blaise Kovohouande, Gérard Agboton, Rene Ahossi, Raoul Baderha Ngabo, Nathan Bisimwa, Covalic Melic Bokossa Kandokponou, Mireille Dokponou, Francis Moïse Dossou, Corinne Dzemta, Antoine Gaou, Roland Goudou, Emmanuel Hedefoun, Sunday Houtoukpe, Felix Kamga, Eric Kiki-Migan, Souliath Lawani, Ismaïl Lawani, René Loko, Afissatou Moutaïrou, Pencome Ogouyemi, Fouad Soumanou, Pia Tamadaho, Mack-Arthur Zounon, Luke Aniakwo Adagrah, Bin Baaba Alhaji Alhassan, Mabel Pokuah Amoako-Boateng, Anthony Baffour Appiah, Alvin Asante-Asamani, Benedict Boakye, Samuel A Debrah. Donald Enti, Rahman Adebisi Ganiyu, Patience Koggoh, Richard Kpankpari, Isabella Naa M. Opandoh, Meshach Agyemang Manu, Maison Patrick Opoku Manu, Samuel Mensah, Martin Tangnaa Morna, John Nkrumah, Michael Nortey, Emmanuel Owusu Ofori, Elizaberth Mercy Quartson, Esther Adjei-Acquah, Vera Agyekum, Eric Agyemang, Rebecca Adjeibah Akesseh, Forster Amponsah-Manu, Richard Ofosu-Akromah, Ato Oppong Acquah, Leslie Issa Adam-Zakariah, Esther Asabre, Ruby Acheampong Boateng, Barbara Koomson, Ataa Kusiwaa, Emmanuel Yaw Twerefour, James Ankomah, Frank Assah-Adjei, Anthony Appiah Boakye, Godfred Fosu, Godwin Serbeh, Kofi Yeboah Gyan, Isaac Omane Nyarko, Zelda Robertson, Ralph Armah, Christopher Asare, Delali Akosua Gakpetor, Victoria Sena Gawu, Ambe Obbeng, Doris Ofosuhene, Dorcas Osei-Poku, Diana Puozaa, Enoch Tackie, Arkorful Ebenezer Temitope, Regina Acquah, James Amoako, Akosua Dwamena Appiah, Mark Aseti, Charles Banka, Samuel Dadzie, Derick Essien, Frank Enoch Gyamfi, Romeo Hussey, Jemima Kwarteng, Naa Anyekaa Sowah, Grace Yeboah, Cynthia Yeboah, Kwame Gyambibi Addo, Enoch Appiah Akosa, Percy Boakye, Christian Larbi Coompson, Brian Gyamfi, Bismark Effah Kontor, Christian Kyeremeh, Ruth Manu, Elijah Mensah, Friko Ibrahim Solae, Gideon Kwasi Toffah, Dorcas Otuo Acheampong, Jane Acquaye, Michael Adinku, Kwabena Agbedinu, Anita Eseenam Agbeko, Emmanuel Gyimah Amankwa, Michael Amoah, George Amoah, Juliana Appiah, Joshua Arthur, Alex Ayim, Emmanuel Kafui Ayodeji, Jonathan Boakye-Yiadom, Edward Amoah Boateng, Charles Dally, Anthony Davor, Christian Kofi Gyasi-Sarpong, Naabo Nuhu Noel Hamidu, Iddrisu Haruna, Naa Kwarley, Agbenya Kobla Lovi, Boateng Nimako, Bertina Beauty Nyadu, Dominic Opoku, Anita Osabutey, Robert Sagoe, Samuel Tuffour, Yaa Tufour, Francis Akwaw Yamoah, Abiboye Cheduko Yefieye, Joseph Yorke, Nii Armah Adu-Aryee, Faisal Adjei, Erica Akoto, Elikem Ametefe, Joachim Kwaku Amoako, Godsway Solomon Attepor, George Darko Brown, Benjamin Fenu, Philemon Kwame Kumassah, David Olatayo Olayiwola, Theodore Wordui, Nelson Agboadoh, Fatao Abubakari, Cletus Ballu, Charles Gyamfi Barimah, Guy Casskey Boateng, Prosper Tonwisi Luri, Abraham Titigah, Frank Owusu, Raphael Adu-Brobbey, Christian Larbi Coompson, Abdul-Hamid Labaran, Junior Atta Owusu, Vivian Adobea, Amos Bennin, Fred Dankwah, Stanley Doe, Ruth Sarfo Kantanka, Ephraim Kobby, Kennedy Kofi Korankye Hanson Larnyor, Edwin Osei, Prince Yeboah Owusu, Clement Ayum Sie-Broni, Marshall Zume, Francis Atindaana Abantanga, Darling Ramatu Abdulai, Daniel Kwesi Acquah, Emmanuel Ayingayure, Imoro Osman, Sheba Kunfah, Gbana Limann, Shamudeen Alhassan Mohammed, Sheriff Mohammed, Yakubu Musah, Bernard Ofori, Emmanuel Abem Owusu, Abdul-Hafiz Saba, Anwar Sadat Seidu, Stephen Tabiri, Mustapha Yakubu, Edwin Mwintiereh Taang Yenli, Arun Gautham, Alice Hepzibah, Grace Mary, Deepak Singh, Dimple Bhatti, William Bhatti, Karan Bir, Swati Daniel, Tapasya Dhar, Jyoti Dhiman, Dhruva Ghosh, Sunita Goyal, Ankush, Goyal, Monika Hans, Parvez Haque, Samuel Konda, Anil Luther, Amit Mahajan, Shalini Makkar, Kavita Mandrelle, Vishal Michael, Partho Mukherjee, Reuben Rajappa, Prashant Singh, Atul Suroy, Ravinder Thind, Alen Thomas, Arti Tuli, Sreejith Veetil, Esther Daniel Mark Jesudason, Priyadarshini K, Latha Madankumar, Rohin Mittal, Ida Nagomy, Rajesh Selvakumar, Bharat Shankar, Moonish Sivakumar, Rajeevan Sridhar, Cecil Thomas, Devabalan Titus, Manisha Aggarwal, Parth Dhamija, Himani Gupta, Vinoth Kanna, Ashwani Kumar, Gurtaj Singh, Philip Alexander, Josy Thomas, Pradeep Zechariah, Amos Dasari, Priya Jacob, Elizabeth Kurien, Arpit Mathew, Danita Prakash, Anju Susan, Rose Varghese, Rahul Alpheus, Ashish Choudhrie, Hemanth Kumar, Nitin Peters, Subrat Raul, Rajeev Sharma, Rakesh Vakil, Wenceslao Ángeles Bueno, Francisco Barbosa Camacho, Aldo Bernal Hernández, Ana Bogurin Arellano, Edgar Cortes Torres, Clotilde Fuentes Orozco, Erick González García de Rojas, Alejandro González Ojeda, Bertha Guzmán Ramírez, Michel Hernández Valadez, Diego Luna Acevedo, Rubén Morán Galaviz, Oscar Olvera Flores, José Pérez Navarro, Kevin Pintor Belmontes, Fernando Ramirez Marbello, Luis Ramírez-González, Laura Reyes Aguirre, Ramona Rojas García, Eduardo Valtierra Robles, Reyes Cervantes Ortiz, Gonzalo Hernandez Gonzalez, Rosa Hernandez Krauss, Luis Hernández Miguelena, Marco Hurtado Romero, Isaac Baltazar Gomez, Celina Cuellar Aguirre, Alejandro Cuevas Avendaño, Luis Dominguez Sansores, Hector Ortiz Mejia, Laura Urdapilleta Gomez del Campo, Claudia Caballero Cerdan, David Dominguez Solano, Rafael Toriz Garcia, Mariana Barreto Gallo, Ana Cortes Flores, Alejandro Gonzalez Ojeda, Monica Jimenez Velasco, Rozana Reyes Gamez, Roque Lincona Menindez, Alberto Navarrete Peón, Maria Paz Muñoz, Irán Irani Durán Sánchez, Diana Samantha González Vázquez, María José Martínez Lara, Laura Martinez Perez Maldonado, Alejandra Nayen Sainz de la Fuente, Antonio Ramos De la Medina, Lawal Abdullahi, Khadija Ado, Mohammed Aliyu, Lofty-John Anyanwu, Mahmoud Magashi, Abubakar Muhammad, Saminu Muhammad, Bello Muideen, Idris Takai, Onyekachi Ukata, Opeoluwa Adesanya, David Awonuga, Olushola Fasiku, Chidiebere Ogo, Moruf Abdulsalam, Abimbola Adeniran, Olalekan Ajai, Olukemi Akande, Kazeem Atobatele, Grace Eke, Omolara Faboya, Zainab Imam, Esther Momson, Francisca Nwaenyi, Ayokunle Ogunyemi, Mobolaji Oludara, Olufunmilade Omisanjo, Olabode Oshodi, Yusuf Oshodi, Yemisi Oyewole, Omotade Salami, Omolara Williams, Victoria Adeleye, Adesoji Ademuyiwa, Oluwafunmilayo Adeniyi, Opeyemi Akinajo, David Akinboyewa, Iyabo Alasi, Felix Alakaloko, Oluwole Atoyebi, Olanrewaju Balogun, Orimisan Belie, Christopher Bode, Andrew Ekwesianya, Olumide Elebute, Francis Ezenwankwo, Adedeji Fatuga, George Ihediwa, Adesola Jimoh, Jubril Kuku, Oluwaseun LadipoAjayi, Ayomide Makanjuola, Olayanju Mokwenyei, Samuel Nwokocha, Olubunmi Ogein, Rufus Ojewola, Abraham Oladimeji, Thomas Olajide, Oluwaseun Oluseye, Justina Seyi-Olajide, Adaiah Soibi-Harry, Aloy Ugwu, Emmanuel Williams, Ochomma Egwuonwu, Okechukwu Ekwunife, Victor Modekwe, Chukwuemeka Okoro, Chisom Uche, Kenneth Ugwuanyi, Chuka Ugwunne, Akeem Adeleke, Wilson Adenikinju, Olumide Adeniyi, Akinfolarin Adepiti, Adewale Aderounmu, Abdulhafiz Adesunkanmi, Adewale Adisa*, Samuel Ajekwu, Olusegun Ajenjfuja, Jerrie Akindojutimi, Akinbolaji Akinkuolie, Olusegun Alatise, Olubukola Allen, Lukmon Amosu, Micheal Archibong, Olukayode Arowolo, Deborah Ayantona, Ademola Ayinde, Olusegun Badejoko, Tajudeen Badmus, Amarachukwu Etonyeaku, Emeka Igbodike, Omotade Ijarotimi, Adedayo Lawal, Fayowole Nana, Tunde Oduanafolabi, Olalekan Olasehinde, Olaniyi Olayemi, Stephen Omitinde, Owolabi Oni, Chigozie Onyeze, Ernest Orji, Adewale Rotimi, Abdulkadir Salako, Olufemi Solaja, Oluwaseun Sowemimo, Ademola Talabi, Mohammed Tajudeen, Funmilola Wuraola, Francis Adebayo, Oseremen Aisuodionoe-Shadrach, Godwin Akaba, Lazarus Ameh, Ndubuisi Mbajiekwe, Felix Ogbo, Samson Olori, Olabisi Osagie, Abu Sadiq, Samuel Sani, Nancy Tabuanu, Martins Uanikhoba, Godwin Chiejina, Ekpo Edet, Akan Inyang, Mary Isa, Faith Iseh, Adams Marwa, Sunday Ogbeche, Edima Olory, Gabriel Udie, Joseph Udosen, Usang Usang, Olukayode Abayomi, Rukiyat Abdus-Salam, Sikiru Adebayo, Akinlabi Ajao, Olanrewaju Amusat, Omobolaji Ayandipo, Kelvin Egbuchulem, Hyginus Ekwuazi, Peter Elemile, Taiwo Lawal, Olatunji Lawal, Solomon Olagunju, Peter Osuala, Bamidele Suleman, Augustine Takure, Lukman Abdur-Rahman, Nurudeen Adeleke, Muideen Adesola, Rafiat Afolabi, Sulaiman Agodirin, Isiaka Aremu, Jibril Bello, Saheed Lawal, Abdulwahab Lawal, Hadijat Raji, Olayinka Sayomi, Asimiyu Shittu, Jude Ede, Sebastian Ekenze, Vincent Enemuo, Matthew Eze, Uchechukwu Ezomike, Emmanuel Izuka, Okezie Mbadiwe, Ngozi Mbah, Uba Ezinne, Matthew Francis, Iweha Ikechukwu, Okoi Nnyonno, Philemon Okoro, Igwe Patrick, John Raphael, Oriji Vaduneme, Abhulimen Victor, Salathiel Kanyarukiko, Francine Mukaneza, Deborah Mukantibaziyaremye, Aphrodis Munyaneza, Gibert Ndegamiye, Ronald Tubasiime, Moses Dusabe, Emelyne Izabiriza, Hope Lydia Maniraguha, Christophe Mpirimbanyi, Josiane Mutuyimana, Olivier Mwenedata, Elisee Rwagahirima, Francine Uwizeyimana, Job Zirikana, Aime Dieudonne Hirwa, Elysee Kabanda, Salomee Mbonimpaye, Christine Mukakomite, Piolette Muroruhirwe, Georges Bucyibaruta, Gisele Juru Bunogerane, Sosthene Habumuremyi, Jean de Dieu Haragirimana, Alphonsine Imanishimwe, JC Allen Ingabire, Violette Mukanyange, Emmanuel Munyaneza, Emmanuel Mutabazi, Isaie Ncogoza, Faustin Ntirenganya, Jeannette Nyirahabimana, Christian Urimubabo, Mary Augusta Adams, Richard Crawford, Chikwendu Jeffrey Ede, Maria Fourtounas, Gabriella Hyman, Zafar Khan, Morapedi Kwati, Mpho Nosipho Mathe, Rachel Moore, Ncamsile Anthea Nhlabathi, Hlengiwe Samkelisiwe Nxumalo, Paddy Pattinson, Nnosa Sentholang, Mmule Evelyn Sethoana, Maria Elizabeth Stassen, Laura Thornley, Paul Wondoh Edenvale Hospital, Johannesburg: Cheryl Birtles, Mathete Ivy, Cynthia Mbavhalelo, Zain Ally, Abdus-sami Adewunmi, Jonathan Cook, David Jayne, Soren Laurberg, Julia Brown, Simon Cousens, Neil Smart

## Supplementary Material

znad058_Supplementary_Data

## Data Availability

Anonymized interview and cohort study data are available on request to the TALON Study Management Group, upon successful completion of a Data Sharing Agreement
