# Erratum to: Adaptation of the Wound Healing Questionnaire universal-reporter outcome measure for use in global surgery trials (TALON-1 study): mixed-methods study and Rasch analysis

**DOI:** 10.1093/bjs/znad226

**Published:** 2023-07-18

This is an erratum to: NIHR Global Health Research Unit on Global Surgery, Adaptation of the Wound Healing Questionnaire universal-reporter outcome measure for use in global surgery trials (TALON-1 study): mixed-methods study and Rasch analysis, *British Journal of Surgery*, Volume 110, Issue 6, June 2023, Pages 685–700, https://doi.org/10.1093/bjs/znad058

In the originally published version of this manuscript, James Glasbey's name was included in the author byline. The author list has now been updated to include only the NIHR Global Health Research Unit on Global Surgery.

The Publisher apologizes for not correcting this error during an earlier production stage.

