# Supplementary material for: Adaptation of the Wound Healing Questionnaire universal-reporter outcome measure for use in global surgery trials (TALON-1 study): mixed-methods study and Rasch analysis
Source: Br J Surg. 2023 Apr 3;110(6):685–700. doi: 10.1093/bjs/znad058 (PMC10364512; doi:10.1093/bjs/znad058)
Supplement: znad058_Supplementary_Data [file znad058_supplementary_data.docx]

**Adaptation of the Wound Healing Questionnaire universal-reporter outcome measure for use in global surgery trials (TALON-1 study): mixed-methods study and Rasch analysis**

NIHR Global Health Research Unit on Global Surgery

Correspondence to:

Mr James Glasbey MBBCh BSC PGCert MRCS, NIHR Doctoral Research Fellow in Global Surgery, NIHR Global Health Research Unit on Global Surgery, University of Birmingham, Institute of Translational Medicine, Mindelsohn Way, Birmingham, B15 2TH, Email: [j.glasbey@bham.ac.uk](mailto:j.glasbey@bham.ac.uk)

# Supplementary Materials - Index

[Appendix S1. Collaborating authors (PubMed citable) 3](#_Toc122528786)

[Appendix S2. Adaptation of delivery during SARS-CoV-2 pandemic 21](#_Toc122528787)

[Appendix S3. PCORI reporting checklist 22](#_Toc122528788)

[Appendix S4. Wound Healing Questionnaire (WHQ) development and validation 23](#_Toc122528789)

[Appendix S5. Example topic guide (Site researchers) 24](#_Toc122528790)

[Appendix S6. Coding methodology 26](#_Toc122528791)

[Appendix S7. Translation methodology, performed according to Mapi recommendations 27](#_Toc122528792)

[Appendix S8. Optimisation and quality assurance of WHQ administration 28](#_Toc122528793)

[Appendix S9. Lay description of Rasch analysis 29](#_Toc122528794)

[Appendix S10. Item-by-item summary of triangulation 30](#_Toc122528795)

[Item response categories 30](#_Toc122528796)

[Item 1. Redness 31](#_Toc122528797)

[Item 2. Warmth 32](#_Toc122528798)

[Item 3. Clear fluid 32](#_Toc122528799)

[Item 4. Blood-stained fluid 33](#_Toc122528800)

[Item 5. Purulent fluid 33](#_Toc122528801)

[Item 6. Wound opening 34](#_Toc122528802)

[Item 7. Deep wound opening 34](#_Toc122528803)

[Item 8. Local swelling 36](#_Toc122528804)

[Item 9. Smell 36](#_Toc122528805)

[Item 10. Tenderness 38](#_Toc122528806)

[Item 11. Fever 38](#_Toc122528807)

[Item 12. Advice 39](#_Toc122528808)

[Item 13. Dressing 40](#_Toc122528809)

[Item 14. Readmission 41](#_Toc122528810)

[Item 15. Antibiotics 41](#_Toc122528811)

[Item 16. Clinician wound opening 43](#_Toc122528812)

[Item 17. Wound scraping 44](#_Toc122528813)

[Item 18. Wound drained 44](#_Toc122528814)

[Item 19. Reoperation 46](#_Toc122528815)

[Appendix S11. Adapted version of Wound Healing Questionnaire (English language) 48](#_Toc122528816)

[Appendix S12. Translations of adapted Wound Healing Questionnaire 51](#_Toc122528817)

[Appendix S13. Co-produced toolkit to support optimised implementation of the WHQ pathway 52](#_Toc122528818)

[Table S1. Summary of qualitative data used in adaptation of ‘symptoms’ items (1 to 11) and item response categories 53](#_Toc122528819)

[Table S2. Summary of qualitative data used in adaptation of ‘treatment’ items (12 to 19) 56](#_Toc122528820)

[Table S3. Class interval structure in Rasch analysis 58](#_Toc122528821)

[Table S4. Individual item fit in Rasch analysis 59](#_Toc122528822)

[Table S5. Exploration of item correlations and local dependency between items in Rasch analysis 60](#_Toc122528823)

[Table S6. Exploration of differential item functioning by country 61](#_Toc122528824)

[Table S7. Exploration of differential item functioning by patient home location (urban versus rural) 62](#_Toc122528825)

[Figure S1. Differential item functioning by country for symptoms items 1 (Redness), 8 (Local swelling), 10 (Tenderness), 11 (Fever) 63](#_Toc122528826)

[Figure S2. Threshold probability map after subtest analysis of item 3 (clear fluid), item 4 (blood-stained fluid) and item 5 (purulent fluid) 64](#_Toc122528827)

[Figure S3. Category probability curve upon subtest analysis of item 6 (wound opening) and item 7 (deep wound opening) 65](#_Toc122528828)

[Figure S4. Differential item functioning by country for pathway items 14 (Readmission), 15 (Antibiotics), 16 (Clinician opening), 19 (Reoperated) 66](#_Toc122528829)

[Supplement references 67](#_Toc122528830)

# Appendix S1. Collaborating authors (PubMed citable)

***Writing group*** *(*denotes joint corresponding authors)*

James Glasbey* (UK), Adesoji Ademuyiwa (Nigeria), Alisha Bhatt (India), Bruce Biccard (South Africa), Jane Blazeby (UK), Peter Brocklehurst (UK), Sohini Chakrabortee (UK), JC Allen Ingabire (Rwanda), Francis Moïse Dossou (Benin), Irani Durán (Mexico), Rohini Dutta (India), Dhruva Ghosh (India), Frank Gyamfi (Ghana), Parvez Haque (India), Pollyanna Hardy (UK), Mike Horton (UK), Gabriella Hyman (South Africa), Ritu Jain (India), Oluwaseun Ladipo-Ajayi (Nigeria), Ismail Lawani (Benin), Souliath Lawani (Benin), Mwayi Kachapila (UK), Rachel Lillywhite (UK), Rhiannon Macefield (UK), Laura Magill (UK), Janet Martin (Canada), Jonathan Mathers (UK), Kenneth McLean (UK), Punam Mistry (UK), Rohin Mittal (India), Mark Monahan (UK), Rachel Moore (South Africa), Dion Morton (UK), Moyo Ojo (Nigeria), Faustin Ntirenganya (Rwanda), Emmanuel Ofori (Ghana), Rupert Pearse (UK), Alberto Peón (Mexico), Thomas Pinkney (UK), Antonio Ramos de la Medina (Mexico), Tubasiime Ronald (Rwanda), David Roman (Mexico), Emmy Runingamugabo (Rwanda), Alice Sitch (UK), Anita Slade (UK), Donna Smith (UK), Stephen Tabiri* (Ghana), Aneel Bhangu (UK).

***Data handling and statistical analysis***

James Glasbey, Anita Slade, Mike Horton, Rhiannon Macefield, Aneel Bhangu, Pollyanna Hardy

***National hub lead investigators***

Adesoji O Ademuyiwa, Lawani Ismail, Dhruva Ghosh, Antonio Ramos de la Medina, Rachel Moore, Faustin Ntirenganya, Stephen Tabiri

***Community engagement and involvement partners***

Emmy Runingamugabo (Rwanda), Simin Patrawala (India), Angela Prah (Ghana), Christian Oko (Nigeria), Karolin Kroese (UK)

**TALON-1 collaborators**

***Benin***

Ismaïl Lawani, Francis Moïse Dossou*, Corinne Dzemta, Covalic Melic Bokossa Kandokponou, Souliath LAWANI (Centre Hospitalier Universitaire et Départemental Ouémé-Plateau (CHUDOP), Porto-Novo)

Hulrich Behanzin* (Hopital de Zone de Menontin, Contonou)

Cyrile Kpangon* (Centre Hospitalier Universitaire de Zone de Suru Lere (SLERE), Contonou)

***Ghana***

Bernard Appiah Ofori, Stephen Tabiri*, Abdul-Hafiz Saba, Gbana Limann, Daniel Kwesi Acquah, Shamudeen Mohammed Alhassan, Sheriff Mohammed, Owusu Abem Emmanuel, Yakubu Musah, Yenli Edwin, Sheba Kunfah, Yakubu Mustapha, Abantanga Atindaana Francis, Emmanuel Ayingayure, Gbana Limann (Tamale Teaching Hospital, Tamale)

Forster Amponsah-Manu, Eric Agyemang, Vera Agyekum, Esther Adjei-Acquah, Emmanuel Yaw Twerefour, Barbra Koomson, Ruby Acheampong Boateng, Ato Oppong Acquah, Richard Ofosu-Akromah, Leslie Issa Adam-Zakariah (Eastern Regional Hospital, Koforidua)

Nii Armah Adu-Aryee, Theodore Wordui (Korle-Bu Teaching Hospital, Accra)

Coomson Christian Larbi, Akosa Appiah Enoch, Mensah Elijah, Kyeremeh Christian, Addo Gyambibi Kwame, Boakye Percy, Kontor Effah Bismark, Gyamfi Brian, Manu Ruth (Techiman Holy Family Hospital (THFH), Techniman)

Romeo Hussey, Samuel Dadzie, Akosua Dwamena Appiah, Grace Yeboah, Cynthia Yeboah, James Amoako, Regina Acquah, Naa Anyekaa Sowah (Berekum Holy Family Hospital (BHFH), Berekum)

Atta Kusiwaa, Esther Asabre (Effia-Nkwanta Regional Hospital (ENRH), Takoradi)

Cletus Ballu, Charles Gyamfi Barimah (Salaga District Hospital, Salaga)

Frank Owusu (St. Patrick's Hospital, Offinso)

Clement Sie-Broni, Vivian Adobea, Prince Yeboah Owusu, Marshall Zume, Abdul-Hamid Labaran, Raphael Adu-Brobbey (Sunyani Regional Hospital (SRH), Sunyan)

Martin Tangnaa Morna, Samuel A. Debrah, Patrick Opoku Manu Maison, Michael Nortey, Donald Enti, Mabel Pokuah Amoako-Boateng, Anthony Baffour Appiah, Emmanuel Owusu Ofori, Richard Kpankpari, Benedict Boakye, Elizabert Mercy Quartson, Patience Koggoh (Cape Coast Teaching Hospital, Cape Coast)

Anita Eseenam Agbeko, Frank Enoch Gyamfi, Joshua Arthur, Joseph Yorke, Christian Kofi Gyasi-Sarpong, Charles Dally, Agbenya Kobla Lovi, Michael Amoah, Boateng Nimako, Robert Sagoe, Anthony Davor, Fareeda Galley, Michael Adinku, Jonathan Boakye-Yiadom, Jane Acquaye, Juliana Appiah, Dorcas Otuo Acheampong, Iddrisu Haruna, Edward Amoah Boateng, Emmanuel Kafui Ayodeji, Samuel Tuffuor, Naa Kwarley, Yaa Tufuor (Komfo Anokye Teaching Hospital (KATH), Kumasi)

Ramatu Darling Abdulai, Fred Dankwah, Ralph Armah, Doris Ofosuhene, Dorcas Osei-Poku, Arkorful Ebenezer Temitope, Delali Akosua Gakpetor, Victoria Sena Gawu, Christopher Asare, Enoch Tackie ((Greater) Accra Regional Hospital (ARH), Accra)

James Ankomah, Isaac Omane Nyarko, Zelda Robertson, Serbeh Godwin, Appiah Anthony Boakye, Godfred Fosu, Frank Assah-Adjei (Goaso Municipal Hospital, Goaso)

***India***

Christian Medical College, Ludhiana: Parvez Haque*, Ritu Jain*, Alisha Bhatt, Jyoti Dhiman, Rohini Dutta, Dhruva Ghosh

Christian Medical College, Vellore: Esther Daniel, Priyadarshini K, Latha Madankumar, Rohin Mittal*, Ida Nagomy, Soosan Prasad

Madhipura Christian Hospital, Madhipura: Arpit Jacob Mathew*, Danita Prakash, Priya Jacob, Jeremiah P Anachy, Amy Mathew

Lady Willingdon Hospital, Manali: Josy Thomas, Philip V Alexander*, Pradeep Zechariah, Neerav D Aruldas

Sher-i-Kashmir Institute Of Medical Sciences, Srinagar: Asif Mehraj*,Hafsa Imtiyaz Ahmed, Rauf A Wani, Fazl Q Parray, Nisar A Chowdri

***Mexico***

Antonio Ramos De la Medina*, Laura Martinez Perez Maldonado, Diana S Gonzalez Vazquez, Iran I Durán Sánchez, Maria J Martínez Lara, Alejandra Nayen Sainz de la Fuente (Hospital Español Veracruz, Veracruz)

Ana O Cortes Flores*, Mariana E Barreto Gallo, Alejandro Gonzalez Ojeda, Monica E Jimenez Velasco (Hospital San Javier, Guadalajara)

Luis Hernández Miguelena*, Reyes J Cervantes Ortiz, Gonzalo I Hernandez Gonzalez, Marco Hurtado Romero, Rosa I Hernandez Krauss (Hospital Regional, Veracruz)

Luis A Dominguez Sansores*, Alejandro Cuevas Avendaño, Celina Cuellar Aguirre, Isaac Baltazar Gomez, Hector Ortiz Mejia (Hospital General de Boca Del Rio, Boca del Rio)

Alejandro González Ojeda*, Oscar E Olvera Flores, Erick A González García de Rojas, Kevin J Pintor Belmontes, Francisco J Barbosa Camacho, Aldo Bernal Hernández, Laura Reyes Aguirre, Rubén E Morán Galaviz, Clotilde Fuentes Orozco, Wenceslao G Ángeles Bueno, Fernando S Ramirez Marbello, Diego E Luna Acevedo, Michel Hernández Valadez, Ana L Bogurin Arellano, Luis R Ramírez-González, Bertha G Guzmán Ramírez, Eduardo Valtierra Robles, Ramona I Rojas García, José V Pérez Navarro, Edgar J Cortes Torres (Hospital De Especialidades, CMNO, Guadalajara)

David R Dominguez Solano* (Hospital Regional de Xalapa, Xalapa)

Alberto N Peón*, Roque D Lincona Menindez, Rozana Reyes Gamez, Maria C Paz Muñoz, (Hospital Espanol Pachuca, Puebla)

***Nigeria***

Lagos University Teaching Hospital, Lagos (Hub):Orimisan Belie, Victoria Adeleye, Adesoji Ademuyiwa*, Oluwafunmilayo Adeniyi, Opeyemi Akinajo, David Akinboyewa, Felix Alakaloko, Oluwole Atoyebi, Olanrewaju Balogun, Christopher Bode, Olumide Elebute, Francis Ezenwankwo, Adesiyakan Adedotun, George Ihediwa, Jubril Kuku, Oluwaseun Ladipo-Ajayi, Ayomide Makanjuola, Samuel Nwokocha, Olubunmi Ogein, Rufus Ojewola, Abraham Oladimeji, Thomas Olajide, Iyabo Alasi, Oluwaseun Oluseye, Justina Seyi-Olajide, Adaiah Soibi-Harry, Emmanuel Williams, Agbulu Moses Vincent, Nnamdi Jonathan Duru, Kenneth Uche Onyekachi, Christiana Ashley, Chinelo Victoria Mgbemena, Moyosoluwa Ojo, Olowu Oluyemisi, Iyabode Ikuewunmi, Adeoluwa Adebunmi, Edet Glory Bassey, Ephraim Okwudiri Ohazurike, Olayide Michael Amao, Osunwusi Benedetto Oluwaseun, Emily Doris, Olutola Stephen, Christianah Gbenga-Oke, Olawunmi Olayioye, Olowu Oluyemisi, Kayode Oluremi, Esther Abunimye, Christianah Oyegbola, Olayade Kayode, Adeola Ayoola Orowale,

Omolara M Williams*, Olufunmilade A Omisanjo, Omolara M Faboya, Zainab O Imam, Olabode A Oshodi, Yusuf A Oshodi, Ayokunle A Ogunyemi, Olalekan T Ajai, Francisca C Nwaenyi (Lagos State University Teaching Hospital (LASUTH))

Adewale O Adisa*, Adewale A Aderounmu, Funmilola O Wuraola, Oludayo Sowande (Obafemi Awolowo University Teaching Hospital, Ile-Ife (IFE))

Lukman Olajide Abdur-Rahman*, Jibril Oyekunle Bello, HADIJAT OLAIDE RAJI, Nurudeen Abiola Adeleke, Saheed Abolade Lawal, Rafiat Tinuola Afolabi, Abdulwahab Lawal (University of Ilorin Teaching Hospital (UITH), Ilorin)

Okechukwu Hyginus Ekwunife*, Ochomma Amobi Egwuonwu, Chisom Faith Uche (Nnamdi Azikiwe University Teaching Hospital (NAUTH), Nnewi)

Abubakar Bala AB Muhammad, Saminu S Muhammad, Idris Usman IU Takai, Mohammed AS Aliyu Salele, Onyekachi G Ukata, Mahmoud Kawu MK Magashi, Lawal Barau LB Abdullahi, Bello Abodunde BA Muideen, Khadija A Ado, Lofty-John Chukwuemeka LJC Anyawu* (Aminu Kano Teaching Hospital, Kano)

Samson Olori*, Samuel A Sani, Olabisi O Osagie, Ndubuisi Mbajiekwe, Oseremen Aisuodionoe-Shadrach, Godwin O Akaba, Lazarus Ameh, Lazarus Ameh, Francis o Adebayo, Martins Uanikhoba, Felix o Ogbo, Nancy O Tabuanu (University of Abuja Teaching Hospital, Abuja)

Taiwo A Lawal*, Rukiyat A Abdus-Salam, Akinlabi E Ajao, Augustine O Takure, Omobolaji O Ayandipo, Hyginus O Ekwuazi, Olukayode Abayomi, Olatunji O Lawal, Solomon Olagunju, Kelvin I Egbuchulem, Sikiru Adekola Adebayo, Peter Elemile (University College hospital, Ibadan)

Usang E Usang*, Joseph E Udosen, Expo E Edet, Akan W Inyang, Edima M Olory, Gabriel U Udie, Godwin O Chiejina, Adams D Marwa, Faith J Iseh, Sunday A Ogbeche, Mary O Isa (University of Calabar Teaching Hospital, Calabar)

Uchechukwu O Ezomike, Sebastian O Ekenze*, Matthew I Eze, Emmanuel O Izuka, Jude K Ede, Vincent C Enemuo, Okezie M Mbadiwe, Ngozi G Mbah (University of Nigeria Teaching Hospital (UNTH) Enugu)

***Rwanda***

Alphonsine Imanishimwe, Sosthene Habumuremyi, Faustin Ntirenganya*,JC Allen Ingabire, Isaie Ncogoza, Emmanuel Munyaneza, Jean de Dieu Haragirimana, Christian Jean Urimubabo, Violette Mukanyange, Jeannette Nyirahabimana, Emmanuel Mutabazi (Rwanda University Teaching Hospital of Kigali (CHUK), Kigali)

Christophe Mpirimbanyi*, Olivier Mwenedata, Hope Lydia Maniraguha, Emelyne Izabiriza, Moses Dusabe, Job Zirikana, Francine Uwizeyimana, Josiane Mutuyimana, Elisee Rwagahirima (Kibungo Referral Hospital (KIBUN), Ngoma District)

Alphonsine Imanishimwe, Ronald Tubasiime*, Aphrodis Munyaneza, Sosthene Habumuremyi, Salathiel Kanyarukiko, Gibert Ndegamiye, Francine Mukaneza, Jean Claude Uwimana, Pierrine Nyirangeri, Deborah Mukantibaziyaremye (Kibogora Hospital (KIBO), Kirambo)

Aime Dieudonne Hirwa*, Salomee Mbonimpaye, Piolette Muroruhirwe, Christine Mukakomite, Elysee Kabanda (Ruhengeri Referral Hospital, Musanze District)

***South Africa***

Rachel Moore, Ncamsile Anthea Nhlabathi, Maria Fourtounas, Mary Augusta Adams, Gabriella Hyman*, Hlengiwe Samkelisiwe Nxumalo, Nnosa Sentholang, Mmule Evelyn Sethoana, Mpho Nosipho Mathe (Chris Hani Baragwanath Hospital, Johannesburg)

Zain Ally* (Helen Joseph Hospital, Johannesburg)

Margot Flint, Bruce Biccard (Groote Schuur Hospital, Cape Town)

**FALCON Trial Investigators**

***FALCON writing group***

Adesoji O Ademuyiwa, Adewale O. Adisa, Aneel Bhangu, Peter Brocklehurst, Sohini Chakrabortee, Pollyanna Hardy, Ewen Harrison, JC Allen Ingabire, Parvez D Haque, Lawani Ismail, James Glasbey, Dhruva Ghosh, Frank Enoch Gyamfi, Elizabeth Li, Rachel Lillywhite, Antonio Ramos de la Medina, Rachel Moore, Laura Magill, Dion Morton, Dmitri Nepogodiev, Faustin Ntirenganya, Thomas Pinkney, Omar Omar, Joana Simoes, Donna Smith, Stephen Tabiri

***Hub leads***

Adesoji O Ademuyiwa, Lawani Ismail, Dhruva Ghosh, Antonio Ramos de la Medina, Rachel Moore, Faustin Ntirenganya, Stephen Tabiri

***Central Trial Management Group***

Adesoji Ademuyiwa, Aneel Bhangu, Felicity Brant, Peter Brocklehurst, Sohini Chakrabortee, Dhruva Ghosh, James Glasbey, Pollyanna Hardy, Ewen Harrison, Emily Heritage, Lawani Ismail, Karolin Kroese, Carmela Lapitan, Rachel Lillywhite, David Lissauer, Laura Magill, Antonio Ramos de la Medina, Punam Mistry, Mark Monahan, Rachel Moore, Dion Morton, Dmitri Nepogodiev, Faustin Ntirenganya, Omar Omar, Thomas Pinkney, Tracy Roberts, Donna Smith, Stephen Tabiri, Neil Winkles.

***Statistical analysis***

Pollyanna Hardy, Omar Omar

***Patient representatives***

Emmy Runigamugabo, Azmina Verjee

***FALCON collaborators*** *(alphabetical by country and surname; *denotes the hospital Principle Investigator):*

***Benin***

Clinique Universitaire d’Acceuil des Urgences - Centre National Hospitalier Universitaire Hubert Koutoucou MAGA, Cotonou: Pierre Sodonougbo, Pamphile Assouto*, Michel Fiogbe, Houenoukpo Koco, Serge Metchinhoungbe, Hodonou Sogbo

Hopital de Menontin, Cotonou: Hulrich Behanzin*, Djifid Morel Seto, Yannick Tandje Hopital de Zone de Suru Lere, Cotonou: Sosthène Kangni, Cyrile Kpangon*, Marcelin Akpla, Hugues Herve Chobli, Blaise Kovohouande

Centre Hospitalier Universitaire et Départemental de l’Ouémé-Plateau, Cotonou (Hub): Gérard Agboton, Rene Ahossi, Raoul Baderha Ngabo, Nathan Bisimwa, Covalic Melic Bokossa Kandokponou, Mireille Dokponou, Francis Moïse Dossou, Corinne Dzemta, Antoine Gaou, Roland Goudou, Emmanuel Hedefoun, Sunday Houtoukpe, Felix Kamga, Eric Kiki-Migan, Souliath Lawani, Ismaïl Lawani*, René Loko, Afissatou Moutaïrou, Pencome Ogouyemi, Fouad Soumanou, Pia Tamadaho, Mack-Arthur Zounon

***Ghana***

Cape Coast Teaching Hospital, Cape Coast: Luke Aniakwo Adagrah, Bin Baaba Alhaji Alhassan, Mabel Pokuah Amoako-Boateng, Anthony Baffour Appiah, Alvin Asante-Asamani, Benedict Boakye, Samuel A Debrah. Donald Enti, Rahman Adebisi Ganiyu, Patience Koggoh, Richard Kpankpari, Isabella Naa M. Opandoh, Meshach Agyemang Manu, Maison Patrick Opoku Manu, Samuel Mensah, Martin Tangnaa Morna*, John Nkrumah, Michael Nortey, Emmanuel Owusu Ofori, Elizaberth Mercy Quartson

Eastern Regional Hospital, Koforidua: Esther Adjei-Acquah, Vera Agyekum, Eric Agyemang, Rebecca Adjeibah Akesseh, Forster Amponsah-Manu*, Richard Ofosu-Akromah

Effia-Nkwanta Hospital, Sekondi-Takoradi: Ato Oppong Acquah, Leslie Issa Adam-Zakariah*, Esther Asabre, Ruby Acheampong Boateng, Barbara Koomson, Ataa Kusiwaa, Emmanuel Yaw Twerefour

Goaso Municipal Hospital, Goaso: James Ankomah*, Frank Assah-Adjei, Anthony Appiah Boakye, Godfred Fosu, Godwin Serbeh, Kofi Yeboah Gyan, Isaac Omane Nyarko, Zelda Robertson

Greater Accra Regional Hospital, Accra: Ralph Armah*, Christopher Asare, Delali Akosua Gakpetor, Victoria Sena Gawu, Ambe Obbeng, Doris Ofosuhene, Dorcas Osei-Poku, Diana Puozaa, Enoch Tackie, Arkorful Ebenezer Temitope

Holy Family Hospital, Berekum: Regina Acquah, James Amoako, Akosua Dwamena Appiah, Mark Aseti, Charles Banka, Samuel Dadzie, Derick Essien, Frank Enoch Gyamfi*, Romeo Hussey, Jemima Kwarteng, Naa Anyekaa Sowah, Grace Yeboah, Cynthia Yeboah

Holy Family Hospital, Techiman: Kwame Gyambibi Addo, Enoch Appiah Akosa, Percy Boakye, Christian Larbi Coompson*, Brian Gyamfi, Bismark Effah Kontor, Christian Kyeremeh, Ruth Manu, Elijah Mensah, Friko Ibrahim Solae, Gideon Kwasi Toffah

Komfo Anokye Teaching Hospital, Kumasi: Dorcas Otuo Acheampong, Jane Acquaye, Michael Adinku, Kwabena Agbedinu, Anita Eseenam Agbeko*, Emmanuel Gyimah Amankwa, Michael Amoah, George Amoah, Juliana Appiah, Joshua Arthur, Alex Ayim, Emmanuel Kafui Ayodeji, Jonathan Boakye-Yiadom, Edward Amoah Boateng, Charles Dally, Anthony Davor, Christian Kofi Gyasi-Sarpong, Naabo Nuhu Noel Hamidu, Iddrisu Haruna, Naa Kwarley, Agbenya Kobla Lovi, Boateng Nimako, Bertina Beauty Nyadu, Dominic Opoku, Anita Osabutey, Robert Sagoe, Samuel Tuffour, Yaa Tufour, Francis Akwaw Yamoah, Abiboye Cheduko Yefieye, Joseph Yorke

Korle Bu Teaching Hospital, Korle Bu: Nii Armah Adu-Aryee*, Faisal Adjei, Erica Akoto, Elikem Ametefe, Joachim Kwaku Amoako, Godsway Solomon Attepor, George Darko Brown, Benjamin Fenu, Philemon Kwame Kumassah, David Olatayo Olayiwola, Theodore Wordui, Nelson Agboadoh

Salaga District Hospital, Tamale: Fatao Abubakari, Cletus Ballu, Charles Gyamfi Barimah, Guy Casskey Boateng, Prosper Tonwisi Luri* Sandema District Hospital, Sandema: Abraham Titigah*

St. Patrick's Hospital, Offinso: Frank Owusu*

St Theresa’s Hospital, Nkoranza: Raphael Adu-Brobbey, Christian Larbi Coompson*, Abdul-Hamid Labaran, Junior Atta Owusu

Sunyani Regional Hospital, Sunyani: Vivian Adobea, Amos Bennin, Fred Dankwah*, Stanley Doe, Ruth Sarfo Kantanka, Ephraim Kobby, Kennedy Kofi Korankye Hanson Larnyor, Edwin Osei, Prince Yeboah Owusu, Clement Ayum Sie-Broni, Marshall Zume

Tamale Teaching Hospital, Tamale (Hub): Francis Atindaana Abantanga, Darling Ramatu Abdulai, Daniel Kwesi Acquah, Emmanuel Ayingayure, Imoro Osman, Sheba Kunfah, Gbana Limann, Shamudeen Alhassan Mohammed, Sheriff Mohammed, Yakubu Musah, Bernard Ofori, Emmanuel Abem Owusu, Abdul-Hafiz Saba, Anwar Sadat Seidu, Stephen Tabiri*, Mustapha Yakubu, Edwin Mwintiereh Taang Yenli

***India***

Chinchpada Christian Hospital, Chinchpada: Arun Gautham, Alice Hepzibah, Grace Mary, Deepak Singh

Christian Medical College, Ludhiana (Hub): Dimple Bhatti, William Bhatti, Karan Bir, Swati Daniel, Tapasya Dhar, Jyoti Dhiman, Dhruva Ghosh*, Sunita Goyal, Ankush, Goyal, Monika Hans, Parvez Haque*, Samuel Konda, Anil Luther, Amit Mahajan, Shalini Makkar, Kavita Mandrelle*, Vishal Michael, Partho Mukherjee, Reuben Rajappa, Prashant Singh, Atul Suroy, Ravinder Thind, Alen Thomas, Arti Tuli, Sreejith Veetil

Christian Medical College, Vellore: Esther Daniel Mark Jesudason, Priyadarshini K, Latha Madankumar, Rohin Mittal*, Ida Nagomy, Rajesh Selvakumar, Bharat Shankar, Moonish Sivakumar, Rajeevan Sridhar, Cecil Thomas, Devabalan Titus

Government Medical College, Patiala: Manisha Aggarwal, Parth Dhamija, Himani Gupta, Vinoth Kanna, Ashwani Kumar*, Gurtaj Singh Lady Willingdon Hospital, Manali: Philip Alexander*, Josy Thomas, Pradeep Zechariah

Madhipura Christian Hospital, Madhipura: Amos Dasari, Priya Jacob, Elizabeth Kurien, Arpit Mathew*, Danita Prakash, Anju Susan, Rose Varghese

Padhar Hospital, Betul: Rahul Alpheus, Ashish Choudhrie*

Post Graduate Institute Of Medical Education And Research, Chandigarh: Hemanth Kumar, Nitin Peters*

St. Stephens' Hospital, Delhi: Subrat Raul*, Rajeev Sharma, Rakesh Vakil

***Mexico***

Centro Medico Nacional de Occidente, Guadalajara: Wenceslao Ángeles Bueno, Francisco Barbosa Camacho, Aldo Bernal Hernández, Ana Bogurin Arellano, Edgar Cortes Torres, Clotilde Fuentes Orozco, Erick González García de Rojas, Alejandro González Ojeda*, Bertha Guzmán Ramírez, Michel Hernández Valadez, Diego Luna Acevedo, Rubén Morán Galaviz, Oscar Olvera Flores, José Pérez Navarro, Kevin Pintor Belmontes, Fernando Ramirez Marbello, Luis Ramírez-González, Laura Reyes Aguirre, Ramona Rojas García, Eduardo Valtierra Robles

Hospital de Alta Especialidad de Veracruz, Veracruz: Reyes Cervantes Ortiz, Gonzalo Hernandez Gonzalez, Rosa Hernandez Krauss, Luis Hernández Miguelena*, Marco Hurtado Romero

Hospital General de Boca del Rio, Boca del Rio: Isaac Baltazar Gomez, Celina Cuellar Aguirre, Alejandro Cuevas Avendaño, Luis Dominguez Sansores*, Hector Ortiz Mejia, Laura Urdapilleta Gomez del Campo

Hospital Regional de Xalapa, Xalapa: Claudia Caballero Cerdan, David Dominguez Solano*, Rafael Toriz Garcia

Hospital San Javier, Guadalajara: Mariana Barreto Gallo, Ana Cortes Flores*, Alejandro Gonzalez Ojeda, Monica Jimenez Velasco Sociedad Española de Beneficencia A.C, Pachuca: Rozana Reyes Gamez, Roque Lincona Menindez, Alberto Navarrete Peón*, Maria Paz Muñoz

Sociedad Española de Beneficencia, Veracruz (Hub): Irán Irani Durán Sánchez, Diana Samantha González Vázquez, María José Martínez Lara, Laura Martinez Perez Maldonado, Alejandra Nayen Sainz de la Fuente, Antonio Ramos De la Medina*

***Nigeria***

Aminu Kano Teaching Hospital, Kano: Lawal Abdullahi, Khadija Ado, Mohammed Aliyu, Lofty-John Anyanwu*, Mahmoud Magashi, Abubakar Muhammad, Saminu Muhammad, Bello Muideen, Idris Takai, Onyekachi Ukata

Federal Medical Centre, Abeokuta: Opeoluwa Adesanya*, David Awonuga, Olushola Fasiku, Chidiebere Ogo

Lagos State University Teaching Hospital, Lagos: Moruf Abdulsalam, Abimbola Adeniran, Olalekan Ajai, Olukemi Akande, Kazeem Atobatele, Grace Eke, Omolara Faboya, Zainab Imam, Esther Momson, Francisca Nwaenyi, Ayokunle Ogunyemi, Mobolaji Oludara, Olufunmilade Omisanjo, Olabode Oshodi, Yusuf Oshodi, Yemisi Oyewole, Omotade Salami, Omolara Williams*

Lagos University Teaching Hospital, Lagos (Hub): Victoria Adeleye, Adesoji Ademuyiwa*, Oluwafunmilayo Adeniyi, Opeyemi Akinajo, David Akinboyewa, Iyabo Alasi, Felix Alakaloko, Oluwole Atoyebi, Olanrewaju Balogun, Orimisan Belie, Christopher Bode, Andrew Ekwesianya, Olumide Elebute, Francis Ezenwankwo, Adedeji Fatuga, George Ihediwa, Adesola Jimoh, Jubril Kuku, Oluwaseun LadipoAjayi, Ayomide Makanjuola, Olayanju Mokwenyei, Samuel Nwokocha, Olubunmi Ogein, Rufus Ojewola, Abraham Oladimeji, Thomas Olajide, Oluwaseun Oluseye, Justina Seyi-Olajide, Adaiah Soibi-Harry, Aloy Ugwu, Emmanuel Williams

Nnamdi Azikwe University Teaching Hospital, Nnobi: Ochomma Egwuonwu, Okechukwu Ekwunife*, Victor Modekwe, Chukwuemeka Okoro, Chisom Uche, Kenneth Ugwuanyi, Chuka Ugwunne

Obafemi Awolowo University Teaching Hospitals, Ile-Ife: Akeem Adeleke, Wilson Adenikinju, Olumide Adeniyi, Akinfolarin Adepiti, Adewale Aderounmu, Abdulhafiz Adesunkanmi, Adewale Adisa*, Samuel Ajekwu, Olusegun Ajenjfuja, Jerrie Akindojutimi, Akinbolaji Akinkuolie, Olusegun Alatise, Olubukola Allen, Lukmon Amosu, Micheal Archibong, Olukayode Arowolo, Deborah Ayantona, Ademola Ayinde, Olusegun Badejoko, Tajudeen Badmus, Amarachukwu Etonyeaku, Emeka Igbodike, Omotade Ijarotimi, Adedayo Lawal, Fayowole Nana, Tunde Oduanafolabi, Olalekan Olasehinde, Olaniyi Olayemi, Stephen Omitinde, Owolabi Oni, Chigozie Onyeze, Ernest Orji, Adewale Rotimi, Abdulkadir Salako, Olufemi Solaja, Oluwaseun Sowemimo, Ademola Talabi, Mohammed Tajudeen, Funmilola Wuraola

University of Abuja Teaching Hospital, Abuja: Francis Adebayo, Oseremen Aisuodionoe-Shadrach, Godwin Akaba, Lazarus Ameh, Ndubuisi Mbajiekwe, Felix Ogbo, Samson Olori*, Olabisi Osagie, Abu Sadiq, Samuel Sani, Nancy Tabuanu, Martins Uanikhoba

University of Calabar, Teaching Hospital, Calabar: Godwin Chiejina, Ekpo Edet, Akan Inyang, Mary Isa, Faith Iseh, Adams Marwa, Sunday Ogbeche, Edima Olory, Gabriel Udie, Joseph Udosen, Usang Usang*

University College Hospital, Ibadan: Olukayode Abayomi, Rukiyat Abdus-Salam, Sikiru Adebayo, Akinlabi Ajao, Olanrewaju Amusat, Omobolaji Ayandipo, Kelvin Egbuchulem, Hyginus Ekwuazi, Peter Elemile, Taiwo Lawal*, Olatunji Lawal, Solomon Olagunju, Peter Osuala, Bamidele Suleman, Augustine Takure

University of Ilorin Teaching Hospital, Ilorin: Lukman Abdur-Rahman*, Nurudeen Adeleke, Muideen Adesola, Rafiat Afolabi, Sulaiman Agodirin, Isiaka Aremu, Jibril Bello, Saheed Lawal, Abdulwahab Lawal, Hadijat Raji, Olayinka Sayomi, Asimiyu Shittu

University of Nigeria Teaching Hospital, Enugu: Jude Ede, Sebastian Ekenze, Vincent Enemuo, Matthew Eze, Uchechukwu Ezomike, Emmanuel Izuka, Okezie Mbadiwe, Ngozi Mbah

University of Port Harcourt Teaching Hospital, Port Harcourt: Uba Ezinne, Matthew Francis, Iweha Ikechukwu, Okoi Nnyonno, Philemon Okoro*, Igwe Patrick, John Raphael, Oriji Vaduneme, Abhulimen Victor

***Rwanda***

Kibogora Hospital, Nyamasheke: Salathiel Kanyarukiko, Francine Mukaneza, Deborah Mukantibaziyaremye, Aphrodis Munyaneza, Gibert Ndegamiye, Ronald Tubasiime*

Kibungo Referral Hospital, Ngoma: Moses Dusabe, Emelyne Izabiriza, Hope Lydia Maniraguha, Christophe Mpirimbanyi*, Josiane Mutuyimana, Olivier Mwenedata, Elisee Rwagahirima, Francine Uwizeyimana, Job Zirikana

Ruhengeri Referral Hospital, Musanze: Aime Dieudonne Hirwa*, Elysee Kabanda, Salomee Mbonimpaye, Christine Mukakomite, Piolette Muroruhirwe

University Teaching Hospital Kigali, Kigali: Georges Bucyibaruta, Gisele Juru Bunogerane, Sosthene Habumuremyi, Jean de Dieu Haragirimana, Alphonsine Imanishimwe, JC Allen Ingabire, Violette Mukanyange, Emmanuel Munyaneza, Emmanuel Mutabazi, Isaie Ncogoza, Faustin Ntirenganya*, Jeannette Nyirahabimana, Christian Urimubabo

***South Africa***

Chris Hani Baragwanath Hospital, Johannesburg: Mary Augusta Adams, Richard Crawford, Chikwendu Jeffrey Ede, Maria Fourtounas, Gabriella Hyman, Zafar Khan, Morapedi Kwati, Mpho Nosipho Mathe, Rachel Moore*, Ncamsile Anthea Nhlabathi, Hlengiwe Samkelisiwe Nxumalo, Paddy Pattinson, Nnosa Sentholang, Mmule Evelyn Sethoana, Maria Elizabeth Stassen, Laura Thornley, Paul Wondoh Edenvale Hospital, Johannesburg: Cheryl Birtles, Mathete Ivy, Cynthia Mbavhalelo*

Helen Joseph Hospital, Johannesburg: Zain Ally* Sebokeng Hospital, Johannesburg: Abdus-sami Adewunmi*

***Independent Data Monitoring and Ethics Committee***

Jonathan Cook, David Jayne, Soren Laurberg

***Independent Trial Steering Committee (alphabetical)***

Julia Brown, Simon Cousens, Neil Smart

# Appendix S2. Adaptation of delivery during SARS-CoV-2 pandemic

A study protocol was developed and approved by the cross-disciplinary Study Management Group (SMG). Whilst cognitive interviews with patients remains the optimal methodology for cross-cultural and cross-language adaptation of an outcome measure (1, 2), modification was required to progress the study during the SARS-CoV-2 pandemic. Travel was prohibited, and face-to-face outpatient appointments were typically avoided due to concerns related to SARS-CoV-2 transmission (3, 4). Cross-cultural and cross-contextual adaptation was therefore performed through expert review and structured interviews with site researchers.

# Appendix S3. PCORI reporting checklist

| **Standards for qualitative methods** | | **Page references** |
| --- | --- | --- |
| QM-1: State the qualitative approach to research inquiry, design, and conduct | A. Identify and describe evidence gaps that support the need for a qualitative component(s) of the study B. Identify the qualitative approach (eg, ethnography, grounded theory) that will be used, including the purpose, why it is an appropriate approach to answer the research question(s), and how it will be operationalized C. Describe the types of data to be collected, strategies for data collection (eg, focus groups, observations, interviews, documents, audio or video recordings), and when the data will be collected D. Describe how confidentiality will be maintained through data collection, management, analysis, and reporting E. State the computer software program used to assist with analysis | 5  9-10  9-10  8  8 |
| QM-2: Select and justify appropriate qualitative methods sampling strategy | A. Describe and provide the rationale for the sampling strategy (see RQ-3†, RQ-4†, and PC-2‡), including how the strategy flows logically from the qualitative approach and how it fits the research question(s) B. Explain the anticipated sample size, detail any variation in sampling that may occur over the course of study, and state the criteria for deciding when no further sampling is necessary (eg, thematic saturation) C. Describe how the methods will ensure that the data capture the depth of experiences of the participants or phenomenon of interest (see PC-2‡ and PC-3‡) | 9  8-9  10 |
| QM-3: Link the qualitative data analysis, interpretations, and conclusions to the study question | A. State who will be involved in the data analysis and interpretation and describe how their qualifications, training, and expertise equip them to understand and address the complexities and challenges unique to qualitative methods B. Describe data analysis procedures and their link to the study’s research questions C. Describe the process by which inferences and themes will be identified and developed as well as how this process is congruent with the chosen qualitative approach and its methodology D. Describe how conclusions will be derived and how they relate to interpretations and content of the original data | 10  9-10  10  10-11 |
| QM-4: Establish trustworthiness* and credibility of qualitative research | A. State how documentation regarding all phases of the analysis will be captured. Multiple data collection methods (eg, interviews, focus groups, observations) and/or experts with diverse backgrounds can be used to increase trustworthiness, in addition to an inter-coder reliability process B. To enhance credibility, discuss three distinct elements: rigorous techniques and methods, the role of the qualitative researcher, and the value of participants’ perspectives and experiences. Credibility must be explained (see RQ-1†, RQ-2†, and IR-7§) and demonstrated in the analysis in at least one of the following three ways: reflexivity, negative case analysis, and/or member checking | 10-11  11 |
| **Standards for mixed methods research** | |  |
| MM-1: Specify how mixed methods are integrated across design, data sources, and/or data collection phases | A. State which mixed methods approach will be used and describe how it will inform the study procedures B. Describe whether the quantitative and qualitative methods will be sequential, concurrent, or a mixture of both, over time C. Describe how the mixed methods design will integrate qualitative and quantitative approaches at one or more stages of the research process and achieve the intent of the design (eg, by aligning the aims to data collection instruments, procedures and analyses of data, and interpretation of the findings) | 13  13  13 |
| MM-2: Select and justify appropriate mixed methods sampling strategy | A. Provide a clear description of the relationship between the sampling techniques and the generation of different types of data (eg, numeric or closed ended *v* narrative or open ended; see RQ-3†, RQ-4†, and QM-2¶) B. Describe the sampling strategies and outline the temporality with which they will take place as they relate to selected qualitative and quantitative methodologies (see IR-1§, IR-2§, PC-2‡, PC-3‡, and QM-1¶), including a justification of the emergence of other samples that may arise during the study, as applicable | 10-12  10-13 and Figure 1 |
| MM-3: Integrate data analysis, data interpretation, and conclusions | A. Describe the analytic approaches to integration and demonstrate how the analysis plan is congruent with the study design and aims, and that it has been developed based on the methodological approach (eg, either a priori or emergently; see IR-1§, IR-2§, PC-2‡, PC-3‡, QM-1¶, and QM-3¶) B. Identify the order of study components and the points of integration. State who will conduct the integration; describe how their qualifications, training, and expertise equip them to understand and address the complexities and challenges unique to mixed methods analysis; and state how integrated analyses will proceed in terms of the qualitative and quantitative components C. Describe the approach used to interpret integrated data and how conclusions are supported by the context of original qualitative and quantitative findings. Address divergent findings from both qualitative and quantitative components, as well as method-specific biases across the methods (see QM-4¶) | 13  13  15-32 |

# Appendix S4. Wound Healing Questionnaire (WHQ) development and validation

Development of the WHQ included three phases: (1) analysis of existing tool and semi-structured interviews; (2) item development; (3) pre-testing for acceptability and understanding (5). In an English language validation study of 792 patients, the WHQ demonstrated an acceptable scale structure, acceptable inter-rater reliability (Kappa for items ranged between 0⋅40 and 0⋅74) and excellent discrimination (area under receiver operating curve characteristic (AUROC) 0⋅91, 95% confidence interval (CI) 0⋅83 to 0⋅98) of patients with and without SSI (5-7). No adaptation, translation, or validation of the WHQ has been performed for patients in the Global South where health literacy, language and cultural contexts, and digital infrastructure differ substantially.

# Appendix S5. Example topic guide (Site researchers)

Item 1. *Read the question in full.*

**Statement:** Please reflect on your experience of completing in-person and telephone follow-up with patients in both clinical practice and randomised trials.

**Generic probes**

- How would a patient go about answering that question?
- In your experience, how would they arrive at that answer?
- Would it be easy or difficult for them to answer? Why?


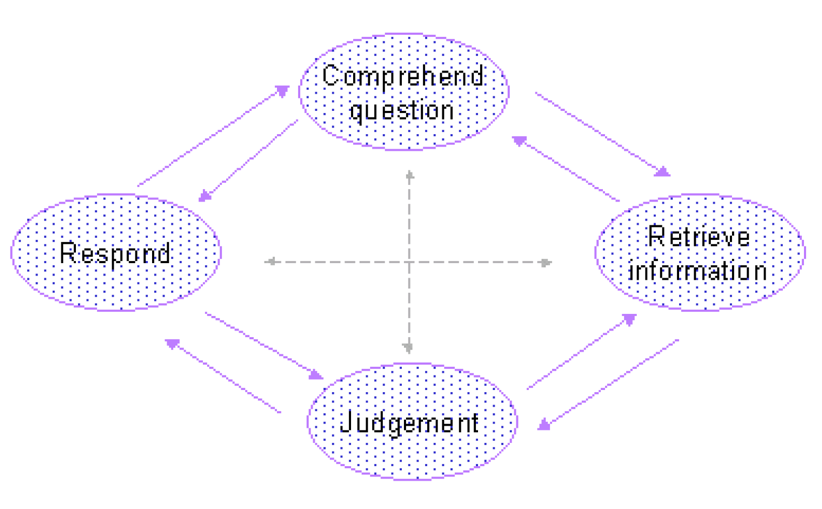


**Figure.** Four-stage question and answer model

**Comprehension probes**

- What do you think the term A means to patients?
- Do you think they will understand the question?
- What have patients had trouble understanding in the past?
- Have you ever needed to provide clarification on this question? Why?
- How have you clarified this question when asking it to patients?

**Retrieval probes**

- Do you think patients typically remember their response to this question?
- How do you think patients you remember that information?
- Do patients ever have problems remembering this information?
- Have you ever made a clarification or prompt to help patients remember?
- What time period do patients talk about when answering this question?

**Judgement probes**

- How sure are patients of their answers?
- Do they ever change their answer? Why?
- What do they talk about when deciding how to answer this question?
- How accurate would you say their answer is? Why?

**Response probes**

- How easy or difficult do patients find it to select an answer from the options provided? Why?
- Are there any categories missing from the options provided or do they cover everything? What is missing?
- Why do you think patients choose a particular answer, rather than one of the others?

# Appendix S6. Coding methodology

First, unrefined data (transcripts and interview notes) from each interview was reviewed during familiarisation. Second, structured WHQ item-by-item summaries were generated for each interview during a charting phase. Thirdly, themes across the four structured categories were coded and explored. To ensure the depth of experience of participants was reflected, we allowed flexibility to include novel, inductive codes related to wound healing, SSI and/or measurement procedures in global contexts, and presented these using thick description (8). Finally, themes were aggregated for each WHQ item to compare and contrast perspectives (9).

# Appendix S7. Translation methodology, performed according to Mapi recommendations

Firstly, an in-country consultant (typically the national PI, or other clinical nominee) was recruited and briefed to oversee the process of translation in the target country and language(s). Secondly, forward translation was performed by two independent translators fluent in both the target and source language (native to the target country). Thirdly, the two versions were compared in detail with any differences in wording highlighted. These were reconciled by discussion between the translators and the in-country consultant. This produced a new translation of the instrument which was reviewed and signed off by the in-country consultant. Fourthly, the translated instrument underwent backwards translation by a third independent translator fluent in both the target and source language (native to the target country). Fifth, the back-translated document was compared to the original instrument. Again, any differences were explored with discussion between the backwards translator, the in-country consultant and a forwards translator (where this was possible). Any further changes to improve clarity in the target language or cross-contextual relevance when then reconciled in the final translated instrument. Sixth, the translated instrument was piloted both between site investigators and between 2 and 4 patients per country. Further clinician review was not deemed to be necessary, with deep clinical involvement throughout the translation process. Finally, a harmonisation meeting was held in an online focus group using the Zoom platform (Zoom Corporation, Tokyo, Japan) to review the in-country consultants’ experience with the instrument and translation process. The instrument was reviewed item-by-time to ensure conceptual equivalence and share new learning in cross-cultural similarities and differences.

# Appendix S8. Optimisation and quality assurance of WHQ administration

In response to Community Engagement and Involvement (CEI (10)) partner feedback, patients were asked to provide two or three contact numbers, which could include a family member or community worker. The researcher was blinded to the outcome of the in-person wound assessment within the FALCON trial, and underwent training from the Study Management Group (SMG). A monitoring call was performed after the local researcher had completed the WHQ for 5-10 patients for quality assurance (pilot). Where recordings were available during this pilot, these were reviewed by a member of the SMG fluent in the target language. A WhatsApp group was also created for all site investigators participating in each country to share early experience and best practice. The pathway for telephone WHQ follow-up was co-designed with patient and community partners to ensure culturally sensitive delivery.

# Appendix S9. Lay description of Rasch analysis

Unidimensionality, measurement properties and cross-cultural item functioning of the WHQ was tested using exploratory Rasch analysis (11). In brief (12), Rasch modelling is a statistical method for exploring a characteristic that is not directly observable (the ‘latent trait’, in this case remote detection of surgical wound infection). A questionnaire measures this by looking at representative behaviours (measured separately in each question or ‘item’) that are combined into one of more scales. Questionnaires provide a raw score for a response to each item that are then summarised to create an overall score. If the questionnaire is to be interpreted correctly, then it should behave like a ruler, where each point score increase indicates the same increase (equal ‘intervals’) in the overall strength of the ‘trait’ (here, the severity of SSI). This sum score can be a misleading if: (1) different items convey more information (‘difficulty’) about the trait than others; (2) if more than one trait is being measured (‘single versus multiple domains’); (3) if patients have a very low (‘floor’) or very high (‘ceiling’) score; (4) if items are very highly correlated so are asking for the same information about the trait; (5) if patients with different characteristics respond in different ways (‘differential item functioning’); (6) in the case of missing item response data. The Rasch model allows exploration (and adjustment) for these patterns and takes into account random variation in item responses, allowing deeper understanding of the ‘person-metric’ properties of a questionnaire and uncertainty around them. Adoption has been widely encouraged to increase quality during questionnaire development and adaptation (11, 13, 14).

# Appendix S10. Item-by-item summary of triangulation

## Item response categories

Investigators from all six countries raised concerns about translatability, comprehension, and judgement between two item response levels for ‘symptoms’ items 1 to 10: (1) ‘A little bit’ and (2) ‘Quite a lot’:

*“The difference between ‘a little bit’ and ‘quite a lot’ is for the very “English” (Surgeon, Focus group NG002F, Nigeria).*

*“I think it is sometimes challenging trying to explain to patients to find the balance where it was... a little versus quite a bit” (Research nurse, Focus group GH001F, Ghana).*

“Quite a bit, a little means the same (in Hindi) I think” (*Surgeon*). “Yes, differentiating between quite a bit and a lot will be a bit difficult for the patient”. (*Research nurse, Focus group IN002F, India*)

Feedback from focus groups also highlighted that many patients might struggle to understand the scalar nature of four item response levels when delivered over the telephone:

*“Questionnaires in Nigeria are more like a conversation than a very structured interaction – it is what is needed to keep the patient engaged. They won’t understand the question as a scale, and the assessor would have to make their best guess” (Research nurse, Interview NG001I, Nigeria)*

In contrast, one included country (Mexico) felt translation of the item would be possible into Spanish language. Investigators agreed that comprehension would remain a problem for less health literate patients:

*“You can translate both ‘a little bit’ and ‘quite a lot’ into Spanish, but its whether they (patients) will understand the difference in real life” (Research nurse, Interview MX001I, Mexico).*

Similarly, investigators from Nigeria, Mexico and Rwanda highlighted that local patients were typically unable to discern whether they had a true fever (Item 11), and many felt ‘hot’ or ‘not right’ after surgery without knowing why:

*“Typically, patients either feel either yes or no, very hot or normal” (Research Nurse, Interview NG002I, Nigeria).*

*“The one with the fever, it’s the answers are a little weird, because you cannot say not at all, a little, quite a bit or a lot, because it’s you had or you have it or you don’t. (Surgical trainee, Focus group MX001F, Mexico).*

In the qualitative harmonisation meeting, investigators proposed a three-level scale (“None”, “A little”, “A lot”) for ‘symptoms’ items in the adapted questionnaire, and two-level scale (“No”, “Yes”) for Item 11 (Fever). Data were collected in the cohort study using the original WHQ item response categories. Upon Rasch analysis, disordered thresholds, where detected for a majority of ‘symptoms’ items: Item 1 (Redness), Item 3 (Clear fluid), Item 6 (Wound opening), Item 7 (Deep tissue opening), Item 9 (Smell) and Item 11 (Fever). A threshold map is shown in *Figure 2* and relevant probability curves in *Figure 4.* Triangulated with qualitative data, this supported reconfiguration of the item response categories in the adapted Wound Healing Questionnaire (summarised in *Table 3*) and was agreed in the final harmonisation meeting.

## Item 1. Redness

Researchers from several countries reported poor cross-contextual relevance of ‘redness of the skin’ and perceived challenges in translating this in a way that would be comprehensible to patients in a local language, particularly for patients with dark skin tones (e.g., in Sub-Saharan Africa):

*“Redness around the wound, from my experience it becomes a bit of a challenge for many of our patients to recognise that redness, bearing in mind the colour of the skin.” (Surgeon, Focus group NG002F, Nigeria).*

*“The red colour is difficult to find in black skin” (Surgeon, Interview BN001I, Benin)*

This was also reflected in cohort study data from Benin, South Africa, Ghana, and Nigeria where redness of the skin demonstrated poor discrimination with significant non-uniform DIF (*Supplementary figure 1*). Investigators suggested that an equivalent concept would be ‘shining’ or ‘tightness’ of the skin and amended wording for this item reached agreement during the final harmonisation meeting.

*“I suggest we substitute shining skin with redness, with dark skin, inflamed skin we tend to ask or we see it shining - that is an adequate replacement” (Surgeon, Focus group NG002F, Nigeria).*

*Recommendation 1:* Amend wording to capture ‘shining of the skin’ to ensure conceptual relevance to dark skin tones. Modify wording during translation into Ghanian dialects to ensure conceptual equivalence.

## Item 2. Warmth

Some concerns were raised about patients’ comprehension, retrieval and judgment for this item, with some investigators worried that

*“it may be (that) people only notice heat if doctor (sic) has told them this (Research nurse, Ghana, Interview GH001I)”.*

However, this item demonstrated acceptable fit to the Rasch model (fit residual -0.574, p=0.427), and there was no significant DIF by country or language.

*Recommendation 2:* No adaptation required.

## Item 3. Clear fluid

Clear fluid was generally considered to be well understood by patients with acceptable retrieval and judgement. A cross-language translatability issue for Ghanian dialects was raised in an interview:

*Patients describe this as ‘water’ from the wound in Ghana (Research nurse, Ghana, Interview GH001I)”.*

*Recommendation 3:* Clarification of consistency of ‘thin’ clear fluid.

## Item 4. Blood-stained fluid

Item 4 was considered to be comprehensible, with no perceived issues in retrieval or judgement. Item fit was satisfactory (fit residual: -1.863, p=0.214) and with no significant DIF. Interesting contextual data was provided from an investigator in Ghana to support retrieval:

*“Family members may also notice bloody leakage from the skin and on the dressings so will be useful help (to the patient) in assessment (Surgeon, Ghana, Interview GH002I)”*

*Recommendation 4:* No cross-cultural adaptation required.

## Item 5. Purulent fluid

No themes were identified in the qualitative data related to this item. However, there was some item misfit on Rasch analysis (fit residual -2.837, p=0.0009). Despite perceived structural dependency by the WHQ developers (5) was there was no significant local dependency seen in residual correlations of Items 3, 4 and 5 (*Supplementary table 3*). Where all three ‘fluid’ items were combined in a subtest (aligned to the structural dependency suggested by the WHQ developers) there was improvement in item fit (fit residual -2.079, p=0.050) and improved ordering of thresholds (*Supplementary figure 2*), but no improvement in overall model fit (χ^2^=199.00, DF:68, p<0.0000001).

*Recommendation 5:* No cross-cultural adaptation required. In future analyses, consider accounting for structural dependency of Item 3, 4 and 5 using subtesting.

## Item 6. Wound opening

Item 6 largely raised issues with retrieval and judgement in the qualitative data. Investigators were particularly concerned about judgement of lower abdominal incisions which can be hidden from direct view:

*“Patients might sometimes need someone to help with a caesarean section or low (abdominal) wound. It can’t tell if a family member isn’t present” (Research nurse, Nigeria, Interview NG001I)*.

Another concern was in retrieval where a wound had opened spontaneously but closed ahead of the 30-day postoperative assessment:

*“(The wound) may have opened, then later closed, so you might need to ask others” (Research nurse, Nigeria, Interview NG001I).*

A translatability issue was raised in Kinyarwanda where the word for ‘opening’ can be interpreted as ‘healing’ (i.e., dressings removed and left to the open air) or as suture removal *(Surgeon, Rwanda, Interview RW001I).* Despite these concerns, item fit was acceptable (fit residual -1.769, p=0.198) and with no significant DIF.

*Recommendation 6:* No cross-cultural adaptation required. Assessors should ask patients to communicate with family members or carers when assessing Item 6 if the wound cannot be directly visualised. Modify wording during translation into Kinyarwanda to ensure conceptual equivalence.

## Item 7. Deep wound opening

There were several issues reported with comprehension and judgment for this question. Investigators felt that both terminology:

*“Deeper tissue is too scientific”* *(Surgeon, Nigeria, Interview NG002I)*

and the anatomy of wound repair would present challenges:

*“Patients will not understand the word tissue or be able to tell the difference between superficial and deeper tissues” (Ghana, Focus group GH001F)*

*“What if they don’t understand or they think the edges are deeper tissue?” (Surgical trainee, Focus group MX001F, Mexico)*

unless they had some prior medical knowledge:

*“(I) don’t think they will be able to say it is a tissue unless it is a medical person, unless they are medical or have been told” (Research nurse, India, Interview IN002I).*

This was highlighted particularly for patients:

*“….with lower literacy, (who) will be unable to appreciate differences” (Surgeon, Mexico, Interview MX002I).*

However, data completeness for this item was high (completed in 46 of 46 patients with ‘a little’ or more wound opening in Item 6) and item fit was acceptable (fit residual 0.407, p=0.312). A solution proposed by investigators in India and Rwanda was to highlight ‘the inside sutures’ in the item description, as something clearly visible in the deep wound space:

*“In Rwanda, we commonly ask patients ‘did you also see the blue sutures’ (deep sutures) to help make this clear” (Surgeon, Rwanda, Interview RW002I).*

Investigators also highlighted the need for safety netting in settings with low health literacy and limited access to care:

*“Patients will want a ‘solution’… ‘next-steps’…so their needs to be clear pathway for safety netting” (Research nurse, Nigeria, Interview NG001I)*.

This item was structurally dependent with Item 6 (wound opening) and found to be locally dependent when exploring residual correlations (co-efficient: +0.33, *Supplementary table 3*). It had a high degree of correlation with several others: item 4 (Blood-stained fluid), item 14 (readmission), item 19 (reoperation). Where item 6 and 7 were analysed together as a subtest, there were still overlapping probability categories (*Supplementary figure 3*), but the individual item fit improved. Issues with translatability into Spanish where also raised, with the clarification of ‘inside sutures’ perceived to be important:

*“It’s hard to translate for ‘deeper tissues’ and I don’t think they (patients) will understand it” (Researcher, Mexico, Interview MX001I).*

*Recommendation 7:* Reword item to support comprehension and judgement by including ‘the flesh beneath the skin or the inside sutures’. In future analyses, consider accounting for structural and local dependency of Item 6 and 7 using subtesting and collapsed item response categories, or exploring Rasch model fit with this item excluded. The assessor should ensure safety netting via a local clinical pathway if any concerns about deep wound opening, and the patient has not yet sought care.

## Item 8. Local swelling

In general, comprehension and retrieval were thought to be acceptable for Item 8:

*“Patients will easily be able to report areas of swelling and whether or not this is around the wound” (Research Nurse, India, Interview IN002I).*

However, there were some issues raised with judgment in comparison to normal healing processes:

*“Patients may confuse (it with) swelling or hardness of the wound related to regular healing” (Surgeon, Ghana, Interview GH002I).*

Potential variation in judgement across contexts was corroborated in DIF analysis, displaying uniform DIF by country (F-statistic 7.71, P=0.000005, *Supplementary figure 1*). However, item fit was acceptable (fit residual -1.25, p=0.109).

*Recommendation 8:* No cross-cultural adaptation required. Consider splitting item for DIF by country in future analyses.

## Item 9. Smell

The inclusion of this item was felt to be very important across cultures:

*“(This is a)….useful symptom to collect as (it’s) commonly reported in patients with SSI in Mexico but not part of current SSI assessment in FALCON” (Researcher, Mexico Interview MX002I)*.

Although some investigators, in particular research nurses, were worried about eliciting this information from patients:

“*Patients may be very ashamed to share this information, and only provide the answer if trust is given (to the interviewer). Especially the female gender, some males too, and probably young adults.” (Research nurse, Nigeria, Interview NG001I)*

others felt that something this notable would be highly likely to be reported:

*“Patients hate to feel abnormal and they will definitely report something so disturbing” (Surgeon, Ghana, Interview GH002I).*

*“No, they will not be offended by this and be able to answer, because they know it’s a part of the routine examination or post-operative follow-up.” (Surgeon, Focus group IN002F, India).*

Investigators also acknowledged the important role of healthcare workers and family members in accurate assessment of this item:

*“(Patients are)….most likely to notice (the wound being smelly) during wound dressing changes by a relative or health workers” (Research nurse, Nigeria, Interview NG001I).*

One potential issue with judgment was proposed in distinguishing wound smells from other sources:

*““I think it depends on the smelly, because the lotions used to dress the wound sometimes they may come with… for example povidone iodine… though it’s not an offensive smell.” (Research nurse, Nigeria, Interview NG001I).*

In keeping with this there was significant item misfit (fit residual -3.52, p=0.000044), although no significant DIF and no local dependency.

*Recommendation 9:* No cross-cultural adaptation required. Advise assessor to build rapport with patients early during measurement procedure, ask family members whether they have noticed the wound being ‘smelly’ during dressing changes, and reassure patient to share symptoms if possible.

## Item 10. Tenderness

Investigators were concerned with patients’ ability to judge normal postoperative pain from pain associated with SSI:

“*Patients struggle to tell what is ‘normal pain’ and pain related to SSI” (Research nurse, Ghana, Interview GH001I).*

Others highlighted that patients are discouraged from touching their wound after surgery, but

*“…their wound may also be painful during daily activities if patients had chosen not to touch the wound or area around it” (Research nurse, India, Interview IN001I).*

Other activities where patients may have their wound touched by others were also highlighted:

*“In Ludhiana most of the ladies just answer for these questions while changing the dressings they feel pain. Yeah, otherwise they don’t touch so much.” (Research nurse, Focus group IN002F, India).*

The item fitted poorly with the Rasch model (fit residual 2.54, p=0.000015), and demonstrated significant uniform (F-statistic 14.19, p=0.000005) and non-uniform DIF (F-statistic 2.63, p=0.0009) by country. It demonstrated underdiscrimination in most countries (*Supplementary figure 1*). The overall fit for the model only slightly improved when Item 10 was removed (χ^2^ 176.55, DF 55, p<0.0000001).

*Recommendation 10:* No cross-cultural adaptation required. Considered dropping item if replicated in future validation studies.

## Item 11. Fever

Conflicting themes arose related to comprehension versus judgement for Item 11. Comprehension was generally considered to be good:

*“Patients have lots of experience reporting temperature as this is a malaria area…patients are usually able to report this well” (Surgeon, Ghana, Interview GH002I).*

However, many investigators reported concerns about judgement. This largely followed two themes: (1) patients very commonly reporting this symptom, particularly in warm environments; (2) lack of access to medical thermometers in the community:

*“Patients all feel temperature rises, as the weather is very hot, and they are very anxious after surgery. Unless they have a thermometer it’s very difficult to for them to know”* *(Surgeon, Rwanda, Interview RW002I).*

Another investigator in Rwanda reported:

*“(Fever)… may not be useful to wound healing in Rwanda. It doesn’t adapt well to our patients” (Focus group, Rwanda, RW001F).*

The inclusion of a degree Celsius cut-off in the item was universally considered to have low contextual relevance in low resource settings:

*“Patients just do not have access to thermometers, so temperatures are never accurate” (Focus group, India, IN001F).*

*“In Mexico, it’s patients say that they have a temperature or fever but they don’t really measure the temperature. They just feel a little hot and they say oh I have temperature, I have fever.” (Surgeon, Focus group MX001F, Mexico)*

Corroborating this, item 11 demonstrated significant misfit (fit residual 0.172, p=0.00004), but no significant DIF or dependency. The model fit did not improve when Item 11 was deleted (χ^2^ 191.30, DF 72, p<0.0000001).

*Recommendation 11.* Remove reference to >38^oC^ as patients do not have the ability to record this in low resource settings.

## Item 12. Advice

No themes were identified to improve cross-contextual relevance of Item 12. Comprehension and judgement were considered to be satisfactory:

*“This is easy for patients. They will be given a routine clinic date before discharge, so will be told to come back early if needed” (Research nurse, Ghana, Interview GH001I).*

Some interesting contextual themes arose related to where patients sought help, ranging from traditional healers, community health workers, primary care, district hospitals, and the surgical centre:

*“…some of them they are in the very remotest areas, they can see these in quotes, herbalist, or herbal occupation they may worsen a disease” (Anaesthetist, Focus group GH002F, Ghana)*

*“The majority of patients now go to the nearby clinic or they invite the health worker to their house, or traditional healers. But the majority, say 80 to 90% visit the nearby clinic, or ask the health worker to come down to their house to assess their wound” (Surgeon, Nigeria, Interview NG002I).*

Item 12 fit the Rasch model with no significant DIF.

*Recommendation 12:* No cross-cultural adaptation required.

## Item 13. Dressing

Variation in standard of wound care between included countries led to concern about judgement and retrieval. In India:

*“Patients often go home with wound care packages, or visit the hospital or healthcare centre for wound care” (Research nurse, India, Interview IN001I),*

whereas in Nigeria:

*“The practice here is after the surgery most of the patients when they are discharged on the ward the wound is healed and most of them don’t even go home with dressing - like without the wound covered” (Surgeon, Nigeria, Interview NG002I).*

This was supported by quantitative data where there was significant uniform (F-statistic 15.26, p<0.0000001) and non-uniform (F-statistic 2.27, p=0.00001) DIF by country (*Supplementary figure 4*). However, the item demonstrated good model fit well with no local dependency. There was also concern about comprehension for patients in Ghana, who use the term ‘washing the wound’ to describe wound care and dressing.

*Recommendation 13:* No cross-cultural adaptation required. Consider splitting for DIF in future analyses. Modify wording during translation into Ghanian dialects to ensure conceptual equivalence.

## Item 14. Readmission

Investigators generally felt comprehension and retrieval for Item 14 would be satisfactory as readmission was a substantial ‘event’ during their postoperative journey. However, judgement related to the cause of readmission was considered to be challenging for some:

*“Telling the difference between seeking advice related to a wound problem and going back to hospital for another problem might be difficult” (Research nurse, Ghana, Interview GH001I)*.

In keeping with this, item 14 fitted poorly with the Rasch model (fit residual -4.5, p<0.0000001) with evidence of overdiscrimation (*Supplementary figure 4*). It also demonstrated a high degree of correlation with Item 7 (deep wound opening) and 15 (antibiotics). Some contextual themes related to access to care demonstrated variability in patients’ behaviours across settings:

*“Patients… always return to the hospital with any postoperative problems, as traditional healers wont tamper with surgical wounds” (Focus group, Ghana, GH001F)*.

*Recommendation 14:* No cross-cultural adaptation required. Consider subtesting to account for local dependency with Item 7 and 14 in future analyses.

## Item 15. Antibiotics

Several issues were raised with the cross-cultural and cross-contextual application of Item 15. Researchers described that:

*“I think most of the patients the medicines they take home they can’t tell which one is antibiotic” (Focus group, Ghana, GH001F)*

in particular that there might be:

*“…confusion between pain (medicines) and antibiotics” (Surgeon, Rwanda, Interview RW002I).*

There was particular concern in judgement for patients with low health literacy or socioeconomic status:

*“…accuracy may be variable depending on their level of education and the area they live” (Research nurse, India, Interview IN001I).*

*“Patients are unable to understand the word antibiotic care, especially daily wages workers.” (Research nurse, Focus group IN002F, India).*

Supporting this, item 15 misfit the Rasch model (fit residual 3.89, p=0.000004) with evidence of underdiscrimination (*Supplementary figure 4)*. In contrast, researchers in Mexico reported that:

*“I think there will be no problem with the patients understanding what antibiotics are, because here in Mexico apparently all patients love antibiotics, that’s all what they want, and they are happy if the doctor gave, it’s like… (laughter)” (Researcher, Mexico, Interview* MX002I)

thus:

*“…patients will be very aware of antibiotics”* *(Researcher, Mexico, Interview MX001I).*

Retrieval was also considered to be challenging:

*“Patients might struggle to remember which medications they were on early after surgery” (Focus group, Ghana, GH001F).*

Potential solutions to support judgement were proposed:

*“It may require further explanation or the names of the antibiotics if literate” (Focus group NG002F, Nigeria).*

*“We can ask them what medication was prescribed to them and we can just that yes this is antibiotic and we can put yes here.” (Surgeon, Focus group IN002F, India).*

A surgeon from Rwanda also recommended

*“The form is also useful. In Rwanda, antibiotics usually take the form of capsules” (Focus group, Rwanda, RW001F).*

*Recommendation 15:* Change wording to ‘medicines (antibiotics)’, mirroring other universal reporter outcome measure style items (13 and 18). Assessors should ask patients to read out the name of their medications or describe the colour and form, if possible, to check that they are correctly identified as antibiotics.

## Item 16. Clinician wound opening

In general, comprehension and retrieval were both considered acceptable for Item 16. One participant from Ghana reported some ambiguity in patients’ differentiating suture removal and deliberate wound opening in her practice *(Research nurse, Ghana, Interview GH001I)*. Others highlighted the need to differentiate item 16 from item 19 (Reoperation), which captures a similar concept of clinician intervention for a wound complication:

*“The problem is, it can happen both on wards and in theatre. We need to make sure patients have the difference with the general anaesthetic question” (Focus group, Nigeria, NG002F).*

The word ‘deliberate’ was considered to have specific negative connotations in Nigeria, where both an interview and focus group discussion perceived it to imply medical harm *(Focus group, Nigeria, NG001F):*

*“…this sounds like a malicious act” (Surgeon, Nigeria, NG003).*

On Rasch analysis, there was significant item misfit (fit residual -3.213, p=0.000002) with evidence of overdiscrimination and local dependency with items 17 (Wound scraping), 18 (drained) and 19 (reoperation). A subtest of Item 16, 17, 18 and 19 slightly improved model fit overall (χ^2^ 185.08, DF 64, p<0.0000001). Non-uniform DIF was detected by country (F-statistic 3.04, p=0.000128; *Supplementary figure 4*.

*Recommendation 16:* Remove the term ‘deliberate’ from the item description to avoid negative connotations. Where necessary assessors should clarify that this item refers to clinician wound opening without general anaesthesia (i.e., outside of the operating room). Consider subtesting to account for local dependency with Item 17, 18 and 19 or splitting this item for DIF in future analyses.

## Item 17. Wound scraping

No issues with comprehension or retrieval were raised. Judgment was considered challenging by an investigator from Nigeria who highlighted:

*“Patients will only be able to answer if the doctor explains it to them at the time of debridement” (Surgeon, Nigeria, Interview NG003I).*

There were also issues raised again with the term ‘tissue’, particularly for patients using Ghanian dialects:

*“The word tissue is not used in Ghana. I think unwanted flesh would be okay, they will understand it much better rather than tissue” (Research nurse, Ghana, Interview GH001I).*

Item 17 demonstrated acceptable fit with the Rasch model with no significant DIF.

*Recommendation 17.* Word ‘tissue’ changed to ‘flesh’ to improve translatability and comprehension for patients with lower health literacy.

## Item 18. Wound drained

Comprehension was highlighted as a major issue for Item 18, particularly in understanding of the concept of an abscess:

*“Abscess is not a word that is commonly used or understood by Ghanian patients” (Ghana, Focus group GH001F).*

However, pus was generally considered to be a concept that was well understood:

*“Patients in general will know the word pus but probably not the term 'abscess'” (Research Nurse, India, Interview IN002I).*

and acceptable to patients:

*“Maybe for the word abscess they don’t understand what it is, but knowing that it’s pus it might be easier” (Researcher, Mexico, Interview MX002I)*

with good judgment:

*“…pus is well understood, and patients know abnormal fluid” (Surgeon, Rwanda, Interview RW001I)*

and that translated well across settings:

*“Pus translates well into Kinyarwanda” (Rwanda, Focus group RW001F).*

A suggestion to improve the item was made to increase standardisation with Item 5 (Thick, green fluid):

*“I suggest that ‘yellow or green’ pus would improve the description (Nigeria, Focus group NG002F).”*

Concerns were also raised about crossover with Item 5, which captures a similar concept (drainage of pus) but in a passive (i.e., spontaneous, without intervention) rather than an active (i.e., performed by a clinician) way:

*“It might be hard to notice between wound being actively drained and passively draining 'water'” (Surgeon, Ghana, Interview GH002I).*

Specific translatability issues were highlighted to improve across language adaptation in Hindi:

*“When translating into Hindi, this translates as 'bad blood’” (India, Focus group IN001F).*

And Ghanian dialects:

*“Patients may stay ‘water coming from wound’, but would be able to say whether this is bloody, or yellow and green colour water” (Research nurse, Ghana, Interview GH001I)*

Despite these perceived challenges, item fit was acceptable and with no significant DIF.

*Recommendation 18:* Change wording to yellow or green fluid (pus), mirroring Item 5. Addition of " drained from your wound by a doctor or nurse” to emphasis active event of item 18. Assessor should not state the term ‘abscess’ to improve comprehension but can apply this concept during measurement.

## Item 19. Reoperation

Comprehension was identified as a major cross-cultural issue with the term ‘general anaesthetic’ for item 19:

*“Patients are unlikely to understand the term general anaesthesia” (Mexico, Focus group MX001F).*

*“A patient told me anaesthesia is a medical term, he does not understand it” (Anaesthetist, Focus group GH001F, Ghana).*

An improvement to the item wording was proposed:

*“Have you been put to sleep for an operation on the wound or for treatment on the wound - that would encompass it. Knowing that process where you are asleep, that’s what I can say, not anaesthetic“ (Surgeon, Nigeria, Interview NG002I).*

However, an important cross-contextual clinical point was also made by several investigators about the higher proportion of patients undergoing spinal rather than general anaesthesia in LMICs, due to training, safety and capacity issues. This would not be captured by the current item.

Although retrieval and judgment were generally perceived to be good:

*“Patients likely to be able to answer, as it’s a serious event to return to surgery” (Research nurse, Ghana, Interviewer GH001I).*

Ghanian and Nigerian investigators raised the challenge for patients of judging between reoperation for a wound complication and another problem, as both would include wound revision and/or relaparotomy:

*“Patients will definitely remember this as it’s such a big event. But it will be difficult to tell 'for a wound problem' versus another reason” (Interviewer GH002I)*

A Rwandan surgeon proposed assessors considering clarification of a 'second operation' to support patient’s comprehension (Surgeon, Rwanda, Interview RW002I).

Corroborating some potential issues with comprehension or judgement there was significant item misfit (fit residual -2.497, p=0.000076) and non-uniform DIF (F-statistic 4.25, p<0.0000001, *Supplementary figure 4*). Item 19 had the highest degree of local dependency of all WHQ items, with high correlation with item 7 (Deep wound opening), 14 (Readmission), 16 (Clinician opening), 17 (Wound scraping). A subtest with all these items together improved overall model fit (χ^2^ 167.25, DF 64, p<0.0000001).

*Recommendation 19:* Consensus that general anaesthesia would not be understood across contexts. As spinal anaesthesia is common in some LMICs, this concept would not currently be captured. Wording adapted to collect information about any procedure carried out in the operating room. Assessors may wish to use the term ‘second operation’ to aid comprehension. Consider subtesting to account for local dependency with item 7, 14, 16 and 17 in future analyses.

# Appendix S11. Adapted version of Wound Healing Questionnaire (English language)

**Wound Healing Questionnaire**

**For questions with tick boxes, please tick one box per question.**

The Wound Healing Questionnaire should be completed between 27-30 days after the patient’s operation over the telephone, for patients that will undergo 30-day assessment as part of the trial. The Wound Healing Questionnaire should not be completed by the same person that will complete the standard 30-day Follow-up Form.

|  | | | Trial Number | | | | | | | | |  | | |  | | | | | | |  | | | |  | | |  | | | | |  | |
| --- | --- | --- | --- | --- | --- | --- | --- | --- | --- | --- | --- | --- | --- | --- | --- | --- | --- | --- | --- | --- | --- | --- | --- | --- | --- | --- | --- | --- | --- | --- | --- | --- | --- | --- | --- |
|  |  |  | Centre name | | | | | | | | | ____________________________ | | | | | | | | | | | | | | | | | | | | | | | |
|  |  |  | Patient name | | | | | | | | | ____________________________ | | | | | | | | | | | | | | | | | | | | | | | |
| **Patient status** | | | | | | | | | | | | | | | | | | | | | | | | | | | | | | | | | | | |
| Has the patient died? | | | | - Yes (***please stop at Patient Status***) | | | | | | | | | | | | | | | | | | - No (continue to Follow-up details) | | | | | | | | | | | | | |
| If patient died, date of death | | | | d | | | d | m | | m | y | | | y | | | y | | y | | | | | ***If patient died, an SAE form must be completed*** | | | | | | | | | | | |
| If patient died, main cause of death | | | | _________________________________________________ | | | | | | | | | | | | | | | | | | | | | | | | | | | | | | | |
| **Follow-up pathway** | | | | | | | | | | | | | | | | | | | | | | | | | | | | | | | | | | | |
| Attempts made to connect with patient | | | | - 1 attempt | | | | | | | | | | | | | | | | | | - 2-3 attempts | | | | | | | | | | | | | |
|  |  |  |  | - 4-5 attempts | | | | | | | | | | | | | | | | | | - >5 attempts | | | | | | | | | | | | | |
| Were you able to contact the patient by telephone? | | | | - Yes (please continue) | | | | | | | | | | | | | | | | | | - No (***please stop here***) | | | | | | | | | | | | | |
| If telephone contact was made, date of contact | | | | d | | | d | m | | m | y | | | y | | | y | | y | | | | |  | | | | | | | | | | | |
| *Ask the patient:*  What type of phone are you using for this call? | | | | - Landline phone | | | | | | | | | | | | | | | | - Mobile phone (without a camera) - Commercial call centre | | | | | | | | | | | | | | | |
|  |  |  |  | - Mobile phone (with a camera) | | | | | | | | | | | | | | | |  |  |  |  |  |  |  |  |  |  |  |  |  |  |  |  |
| *Ask the patient:*  Who owns the phone that you're speaking to me on? | | | | - Patient themselves | | | | | | | | | | | | | | | | - Friend or relative | | | | | | | | | | | | | | | |
|  |  |  |  | - Healthcare worker | | | | | | | | | | | | | | | | - Other (please specify):   _______________________ | | | | | | | | | | | | | | | |
| *Ask the patient:* Do you live in an urban (mostly city or town) or rural (mostly countryside) area? | | | | - Urban | | | | | | | | | | | | | | | | - Rural | | | | | | | | | | | | | | | |
| *Ask the patient:*  What is the highest level of education that you have achieved? | | | | - High/secondary school or above | | | | | | | | | | | | | | | | - Did not complete first/ primary school or no formal education | | | | | | | | | | | | | | | |
|  |  |  |  | - First/primary school level | | | | | | | | | | | | | | | |  |  |  |  |  |  |  |  |  |  |  |  |  |  |  |  |
| What language did the patient use to respond to the questionnaire? | | | | - English | | | | | | | | | | | | | | | | - Other (please specify):   _______________________ | | | | | | | | | | | | | | | |
| *If other*: Was the formal translated Questionnaire used? | | | | - Yes, formal questionnaire | | | | | | | | | | | | | | | | - No, translated by questionnaire administrator | | | | | | | | | | | | | | | |
|  |  |  |  | - No, using formal translator | | | | | | | | | | | | | | | |  |  |  |  |  |  |  |  |  |  |  |  |  |  |  |  |
| **Wound Healing Questionnaire** | | | | | | | | | | | | | | | | | | | | | | | | | | | | | | | | | | | |
| **Please read the following statement to the patient:**  We are interested in knowing how the cut(s) of your skin (called your wound(s)) have healed since you left hospital after your surgery. It is fine to ask someone else to help answer some of the questions, for example if you cannot easily see your wound(s). If you have more than one wound, please answer the questions thinking about just one wound. This should be either the wound with which you have had concerns about how it was healing, or the longest wound if there have been no specific concerns. We would like you to think about the wounds on your skin. Some of the questions I am about to ask you relate to some problems that may occur with wound healing. Please note, many people do not experience these problems after having surgery.  **Since you left hospital after having surgery…** | | | | | | | | | | | | | | | | | | | | | | | | | | | | | | | | | | | |
| Was there redness (or shining of the skin) spreading away from the wound? | | | | | | | | | - Not at all | | | | | | | | | | | | - A little | | | | | | | | | | - A lot | | | | |
| Was the area around the wound warmer than the surrounding skin? | | | | | | | | | - Not at all | | | | | | | | | | | | - A little | | | | | | | | | | - A lot | | | | |
| Has any part of the wound leaked thin, clear fluid? | | | | | | | | | - Not at all | | | | | | | | | | | | - A little | | | | | | | | | | - A lot | | | | |
| Has any part of the wound leaked blood-stained fluid? | | | | | | | | | - Not at all | | | | | | | | | | | | - A little | | | | | | | | | | - A lot | | | | |
| Has any part of the wound leaked thick and yellow or green fluid? | | | | | | | | | - Not at all | | | | | | | | | | | | - A little | | | | | | | | | | - A lot | | | | |
| Have the edges of any part of the wound separated or gaped open of their accord? | | | | | | | | | - Not at all | | | | | | | | | | | | - A little | | | | | | | | | | - A lot | | | | |
| *If the wound edges opened:* Did the flesh beneath the skin or the inside sutures also separate? | | | | | | | | | - Not at all | | | | | | | | | | | | - A little | | | | | | | | | | - A lot | | | | |
| Has the area around the wound become swollen? | | | | | | | | | - Not at all | | | | | | | | | | | | - A little | | | | | | | | | | - A lot | | | | |
| Has the wound been smelly? | | | | | | | | | - Not at all | | | | | | | | | | | | - A little | | | | | | | | | | - A lot | | | | |
| Has the wound been painful to touch? | | | | | | | | | - Not at all | | | | | | | | | | | | - A little | | | | | | | | | | - A lot | | | | |
| **Since you left hospital after having surgery…** | | | | | | | | | | | | | | | | | | | | | | | | | | | | | | | | | | | |
| Have you had, or felt like you have had, a raised temperature or fever? | | | | | | | | | | | | | | | | | | | | | | - Yes | | | | | | | | - No | | | | | |
| Have you sought advice because of a problem with your wound, other than at a planned follow-up appointment? | | | | | | | | | | | | | | | | | | | | | | - Yes | | | | | | | | - No | | | | | |
| Has anything been put on the skin to cover the wound? (dressing) | | | | | | | | | | | | | | | | | | | | | | - Yes | | | | | | | | - No | | | | | |
| Have you been back into hospital for a problem with your wound? | | | | | | | | | | | | | | | | | | | | | | - Yes | | | | | | | | - No | | | | | |
| Have you been given medicines (antibiotics) for a problem with your wound? | | | | | | | | | | | | | | | | | | | | | | - Yes | | | | | | | | - No | | | | | |
| Have the edges of your wound been separated by a doctor or nurse? | | | | | | | | | | | | | | | | | | | | | | - Yes | | | | | | | | - No | | | | | |
| Has your wound been scraped or cut to remove any unwanted flesh? | | | | | | | | | | | | | | | | | | | | | | - Yes | | | | | | | | - No | | | | | |
| Has thick, yellow or green fluid (pus) been drained from your wound by a doctor or nurse (abscess)? | | | | | | | | | | | | | | | | | | | | | | - Yes | | | | | | | | - No | | | | | |
| Have you had to go back to the operating room for treatment of a problem with your wound? | | | | | | | | | | | | | | | | | | | | | | - Yes | | | | | | | | - No | | | | | |
| *Ask the patient:* How happy were you with having your follow-up over the telephone? | | | | | | - Very satisfied | | | | | | | | | | - Satisfied | | | | | | | | | | | - Neither satisfied nor dissatisfied | | | | | | | | |
|  |  |  |  |  |  | - Very unsatisfied | | | | | | | | | | - Unsatisfied | | | | | | | | | | |  |  |  |  |  |  |  |  |  |
| **Before you end the call,** inform the patient that **this telephone questionnaire will not replace the 30-day in-person wound assessment** required as part of the trial, and **they must still have their 30-day follow-up appointment**. | | | | | | | | | | | | | | | | | | | | | | | | | | | | | | | | | | | |
| Time taken to complete telephone questionnaire | | | | | | - <10 minutes | | | | | | | | | | | | - 11-20 minutes | | | | | | | | | | | | | | | | | |
|  |  |  |  |  |  | - 21-30 minutes | | | | | | | | | | | | - >30 minutes | | | | | | | | | | | | | | | | | |
| Please add any further comments or details of the telephone follow-up here: | | | | | | | | | | | | | | | | | | | | | | | | | | | | | | | | | | | |
| **Form completed by** | | | | | | | | | | | | | | | | | | | | | | | | | | | | | | | | | | | |
| Job role | - Surgeon | - Other doctor | | | - Nurse | | | | | | | | - Other (please specify): _____________ | | | | | | | | | | | | | | | | | | | | | | |
| Print full name |  | | | | | | | | | | | | | | | | | | | | | | | | | | | | | | | | | | |
| Signature |  | | | Date form completed | | | | | | | | | | | | | d | | | | | | d | | m | m | | y | | | | y | y | | y |

Please note this questionnaire is licensed from Oxford University Innovation (OUI) outcomes group. Please contact OUI to obtain a free license to use this questionnaire for research purposes.

# Appendix S12. Translations of adapted Wound Healing Questionnaire

Translated versions available at:

<https://drive.google.com/drive/folders/1RX_HqOS8rKtC43TznhC4cj-PAO0WpFs5?usp=sharing>

| **India** | Tamil |
| --- | --- |
|  | Bengali |
|  | Hindi |
|  | Punjabi |
| **Ghana** | Dagbani |
|  | Twi |
| **Mexico** | Spanish |
| **Rwanda** | Kinyarwanda |

# Appendix S13. Co-produced toolkit to support optimised implementation of the WHQ pathway


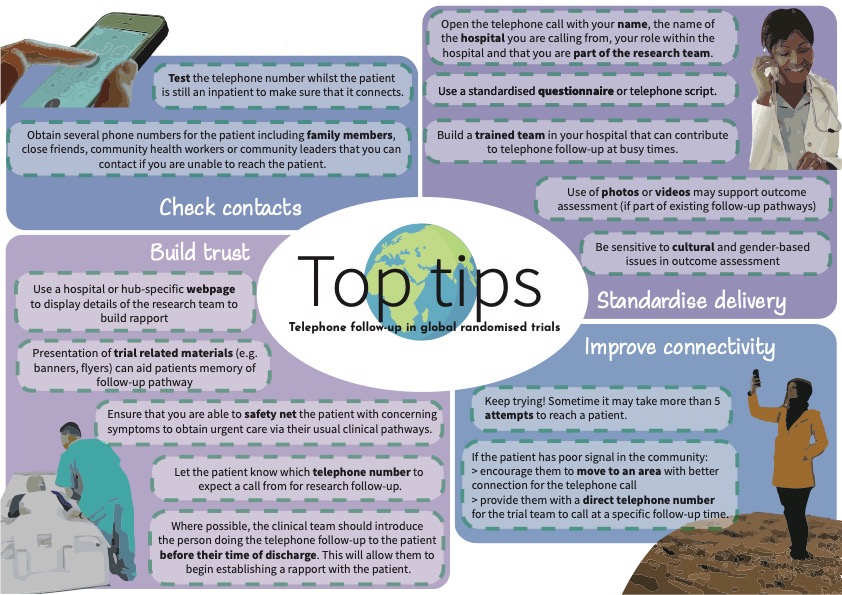


Download the full toolkit presentation at: <https://bit.ly/TALONtips>

# Table S1. Summary of qualitative data used in adaptation of ‘symptoms’ items (1 to 11) and item response categories

| **Thematic domain** | **Original Wound Healing Questionnaire** | | **Item 1** | **Item 2** | **Item 3** | **Item 4** | **Item 5** | **Item 6** | **Item 7** | **Item 8** | **Item 9** | **Item 10** | **Item 11** | |  |
| --- | --- | --- | --- | --- | --- | --- | --- | --- | --- | --- | --- | --- | --- | --- | --- |
|  | **Introductory statement** | **Item response categories: 1 = Not at all; 2 = A little; 3 = Quite a bit; 4 = A lot.** | **Was there redness spreading away from the wound?** | **Was the area around the wound warmer than the surrounding skin?** | **Has any part of the wound leaked clear fluid?** | **Has any part of the wound leaked blood-stained fluid?** | **Has any part of the wound leaked thick and yellow or green fluid?** | **Have the edges of any part of the wound separated or gaped open of their accord?** | **If the wound edges opened, did the deeper tissue also separate?** | **Has the area around the wound become swollen?** | **Has the wound been smelly?** | **Has the wound been painful to touch?** | **Have you had, or felt like you have had, a raised temperature or fever (>38oC)?** | |  |
| **Ghana** | | | | | | | | | | | | | | | |
| Comprehension | None | Four categories hard for questions about symptoms (Interview GH001I). Patients will not understand a four-point scale (Focus group GH001F). | Colour of wound may be challenging for patients with dark skin type (Interview GH002I). | Patients might struggle to answer this question as they are often not health literate (Focus group GH001F). | Patients describe this as 'water' from the wound in Ghana (Interview GH001I). | None | None | None | From FALCON experience, patients will be able to see differences in separation of the deeper part of the wound versus the skin; well reported symptom (Interview GH002I). | Patients should be able to report (Interview GH001I). | None | None | Lots of experience of patients reporting temperature as Ghana is a malarial environment; patients are usually able to report this well (Interview GH002I). | |  |
| Response mapping | N/A | Patients may be more likely to respond appropriately to yes or no item response categories, or not at all, a little, a lot (Interview GH001I). | | | | | | | | | | | | None |  |
| Retrieval | 'Since leaving hospital after having your operation' should be repeated at the top of the second section in order to ensure this is remembered by the assessor and patient. | None | None | The wound may only be open when a nurse/doctor is changing a dressing (Interview GH001I). | None | Family members may also notice leakage from the skin and will be useful collaborators in assessment (Interview GH002I). | None | None | None | None | Patients hate to feel abnormal and they will definitely report something so disturbing (Interview GH002I). | None | None | |  |
| Judgment | N/A | None | From experience within previous randomised trials, may be challenging for patients to judge (Interview GH001I). May only be if answered correctly if the doctor tells patient their observation (Focus group GH001F). | May be that people only notice heat if doctor has told them this (Interview GH001I). | For children in research studies, parents they might have to answer this question for them Interview GH001I). | None | None | Patients would notice during wound dressing or ward round. May see that the wound is open and see flesh below (Interview GH001I). | Patients will not understand the word tissue, or be able to tell the difference between the superficial and deeper tissues (Focus group GH001F). | Patients may confuse swelling/hardness of the wound related to regular healing (Interview GH002I). | None | May struggle in early phases to differentiate normal pain and pain related to SSI. It may help to use the timing of pain, for example excess pain after the first few days after surgery (if discharged early) (Interview GH001I) | Many commonly report 'feeling hot' as the weather often very warm. May be a poor differentiator of those with and without infection (Focus group GH001F) | |  |
| Translatability | None | No terms to differentiate a little & quite a bit in Ghanian languages (Interview GH002I). Particular concern about the differentiation between 'a little' and 'quite a bit' which were perceived to be very 'English English' terms (Focus group GH001F). | None | None | None | None | None | None | None | None | None | None | None | |  |
| Other context | None | None | None | None | None | Patients in Ghana typically stay around the location of the hospital while they’re recovering (Interview GH002I). Most of the time wounds are re-dressed in local hospital (Interview GH001I). | None | None | None | None | Patients will be comfortable to report, did not perceive to have stigma attached (Interview GH001I). | None | Patients rarely have a thermometer so quantifying fever is unhelpful here (Interview GH001I). | |  |
| **Nigeria** | | | | | | | | | | | | | | | |
| Comprehension | None | Patients will not be able to tell the difference between A little and Quite a bit in Nigeria (Interview NG003I). | None | No problems with assessment of this related to CDC criteria to date. Some interviewers may help with understanding in a real world context (Interview NG003). | Colourless, texture may be important (e.g. thin) (Interview NG001I). | None | Memorable & patients will be happy to report this (Interview NG002). | None | None | None | None | None | None | |  |
| Response mapping | N/A | Difficulty differentiating a little and quite a bit over the telephone, as doesn’t appear to be scalar for patients in Nigeria. Might be possible with additional help from person administering the questionnaire if they were able to quantify these levels (Interview NG001I). Quite a bit and a lot not possible to see the difference in. Three point scale would be more appropriate (Focus group NG001F). | | | | | | | | | | | | Typically patients either feel Y/N, very hot or feel normal (Interview NG002I). Patients unable to differentiate between several response categories for fever ((Interview NG003I). |  |
| Retrieval | None | None | None | None | None | None | None | May have opened, then later closed, so may need to ask others/family members. Accuracy may be decreased if family member not available (Interview NG001I). May use having dressing for a longer time than normal or came back to hospital more frequently if wound opened - may help with retrieval (Interview NG003I). | Depending on level of education of the patient, they make not understand difference between deeper tissue (beyond the surface of the skin). Unsure whether they will be able to see other layers of below their skin (Interview NG001I).  Patients won’t understand this, would have to be more can see the deeper layer (fat or flesh or bowel) underneath. Deeper tissue too scientific. (Interview NG002I)  May be too complex for some patients to understand. (Focus group NG001F) | None | None | None | None | |  |
| Judgment | N/A | None | Could be helpful to highlight 'on the surface' of the skin. Redness very challenging in patients in dark skin tone (Interview NG002I). Can be difficult to recognise for patients with dark skin types (Interview NG003I). Difficult to identify in dark skin tones. Patients instead report 'shining' or 'irritated' skin, which would be more appropriate (Interview NG001I). | None | Patients should be asked to think of every part of the wound, particularly the main sutured part of the wound (Interview NG001I). | None | None | Patients might sometimes need an assistant for a lower abdominal wound (dependent on incision site) (Interview NG001I). Patients will report this according to disruption of the line of sutures or separately (Interview NG002I). | Some patients will not be aware, or not able to see whether this has occurred (Focus group NG002F). | Patients would be able to gauge intensity & size of swelling (Interview NG001I). May notice that the wound has grown in size. (Interview NG002) | Patients would picture the smell before and while dressing of the wound, whether an odour was perceived & whether that could be traced back to the wound. They would compare this to a usual smell. May realise if a friend or family member around them had made such compliant. Most likely to notice during wound dressing/changing by relative or health workers, or observed herself/himself. Some wound dressings e.g. iodine may come with their own smell which they won’t be familiar with (Interview NG001I). Patient will consider any offensive odour from the wound (Interview NG002I). | Patients advised not to frequently touch wound, but likely to notice this symptom (Interview NG001I). | At times patients feel odd, but actually have not no temperature. A little bit/quite a bit not directly related to temperature (Interview NG002I). | |  |
| Translatability | N/A | Not all all (i.e. completely zero) and a lot easy to answer. Quite a bit compared to a little very hard in Pidgin English (Interview NG002I). | N/A | | | | | | | | | | | |  |
| Other context | Questionnaire completion in Nigeria is more like a conversation than a very structured interaction in order to keep the patient engaged - the questionnaire can be used to guide this discussion. Patients may 'complete questionnaire' without realising they've answered very structured questions (Interview NG001I). May need skilled administrator. May need to give to relative to help support with responses (Focus group NG001F) | None | None | None | None | None | None | None | Patients with this symptom will want a ‘solution’, ‘next-steps’, so their needs to be clear pathway for safety netting. Currently within FALCON, the research nurse asks when is the next clinic appointment, and tells the patient to mention & the doctor will examine, instructed to continue current medications e.g. antibiotics, and eat a balanced diet with sufficient protein (Interview NG001I). | None | Patients may be very reluctant to share this information/ashamed, and only provide the answer if trust is given to the interviewer, especially females and young adults (Interview NG001I). |  | Patients will not be able to measure their temperature in the community (Focus group NG001F). No patients have access to a thermometer (Focus group NG002F). | |  |
| **India** | | | | | | | | | | | | | | | |
| Comprehension | None | None | None | None | None | None | None | None | None | None | None | None | None | |  |
| Response mapping | None | Patients will not be able to differentiate a little and quite a bit. Recommend a three point scale (Interview IN001I). Should be with a three point, rather than a four point scale for responses for patients in India (Interview IN002I). | | | | | | | | | | | | None |  |
| Retrieval | None | None | None | None | None | None | None | None | None | None | None | None | None | |  |
| Judgment | None | None | None | None | None | None | None | None | Patients will not be able to differentiate between separation of the skin and deeper tissue (Interview IN001I). Can only report Gap in tissues, unless they are medical/been told (Interview IN002I) | Patients will easily be able to report areas of swelling and whether or not this is around the wound (Interview IN002I). | None | The wound may also be painful during daily activities e.g. if patients had chosen not to touch the wound or area around it (Interview IN001I) | None | |  |
| Translatability | None | Most languages will not facilitate nuance around several middle response categories (Focus group IN001F) | None | None | None | None | None | None | None | None | None | None | None | |  |
| Other context | A note should be added to confirm verbal consent before proceeding with the questionnaire. | None | None | None | None | None | None | None | None | None | None | None | Patients do not have access to thermometers – so temperature recording would not be accurate. Better to remove 38oC reference value (Focus group IN001F) | |  |
| **Mexico** | | | | | | | | | | | | | | |  |
| Comprehension | None | None | None | None | None | None | None | None | Some patient groups (e.g. lower literacy) will be unable to appreciate differences between superficial and deep tissues of the wound | None | None | None | None | |  |
| Response mapping | None | Likely to be some problems with patients differentiating between a little & quite a bit (Focus group MX001F). | | | | | | | | | | | | Concern about several levels in the answer to this item, and would prefer as a Yes or No response (Focus group MX001F). |  |
| Retrieval | None | None | None | None | None | None | None | None | None | None | None | None | None | |  |
| Judgment | None | None | None | None | None | None | None | None | None | None | None | None | None | |  |
| Translatability | None | Four scalar options work reasonably well in Spanish language (Interview MX001I). | None | None | None | None | None | None | In Spanish language, no good translation for 'deeper tissues' and may prove a challenge to comprehension (Interview MX001I). | None | None | None | None | |  |
| Other context | None | None | None | None | None | None | None | None | None | None | Useful symptom to collect as commonly reported in patients with SSI in Mexico and not part of current SSI assessment schema (Interview MX002I) | None | Patients do not have access to thermometer, so the number of degrees is unhelpful (Interview MX001I). | |  |
| **Rwanda** | | | | | | | | | | | | | | |  |
| Comprehension | None | None | None | None | None | None | None | Likely to be difficult for patients to understand, and many misunderstand 'opening' as healing (i.e., taking off dressing) or as suture removal. 'Of their own accord' is an important clarification and might need further explanation by the trained assessor (Interview RW001I). | In Rwanda, commonly ask patients 'did you also see the blue sutures' (deep sutures) to help make this clear. Patients do not understand of layered closure, or that there are several layers of the abdominal anatomy (Interview RW002I). | None | None | None | Patients all feel that they have 'temperature rises', as climate very warm and heighten anxiety during recovery, but unless they have a thermometer very difficult to record (Interview RW002I). | |  |
| Response mapping | None | It will be too challenging for patients in Rwanda to differentiate between 'Quite a bit' and "A little' in local languages - the concepts do not transfer well (Interview RW001I) | | | | | | | | | | | | None |  |
| Retrieval | None | None | None | None | None | None | None | None | None | None | None | None | None | |  |
| Judgment | None | None | Not very contextually relevant in Rwanda as patients have very dark skin tones. Will be very hard to judge (Interview RW002I). | None | None | None | None | None | None | None | None | None | May not be capturing a useful concept to wound healing in Rwanda. Doesn't adapt well to Rwandan patients (Focus group RW001F). | |  |
| Translatability | None | Very difficult to translate Quite a Bit versus A little in Kinyarwanda. | Challenging to translate 'redness' - common words in Kinyarwanda relate to 'skin changing from normal' which may be more relevant to patients in Rwanda (Interview RW001I). | None | None | None | None | None | None | None | None | None | None | |  |
| **Benin** | | | | | | | | | | | | | | |  |
| Comprehension | None | None | None | None | None | None | None | None | None | None | None | None | None | |  |
| Response mapping | None | None | None | None | None | None | None | None | None | None | None | None | None | |  |
| Retrieval | None | None | None | None | None | None | None | None | None | None | None | None | None | |  |
| Judgment | None | None | Not possible to identify redness in black skin (Interview BN001I). | None | None | None | None | None | None | None | None | None | None | |  |
| Translatability | None | None | Concept hard to translate in a way that French speaking patients in Benin will understand (Focus group BN001F). | None | None | None | None | None | Tissue is hard to translate for patients in Benin and they may not understand this (Interview BN001I). | None | None | None | None | |  |
| **Summary of recommendations for adaptation and implementation** | No changes to be made. Consent would be confirmed as usual at the start of the interview. | Consensus in qualitative data to reduce the number of response options for symptoms questions to three levels, and to two levels for fever. Decision to triangulate with qualitative data using original response levels in order to make final recommendations for adaptation. | Addition of 'shining of the skin' to support data completion for patients with dark skin tones where redness is difficult to identify. | No changes | Clarification of thin clear fluid | No changes | No changes | No changes.  Assessor can ask family member or friend for help to complete questionnaire where needed. | Mixed opinions about ability of patients to tell the difference between the skin and fleshy part beneath. Therefore decision to reword question, but maintain item in adapted Round 1 questionnaire.   Assessor ensure safety netting via a formal clinician pathway if any concerns. | No changes | No changes | No changes | Consensus to drop >38oC as patients will not have the ability to record this in a global setting so is redundant.  Should be triangulated with data from pilot study will specifically examine whether this should be considered a Yes / No or three-level item. | |  |
| **Adapted Wound Healing Questionnaire** | **Introductory statement** | **1 = Not at all; 2 = A little; 3 = A lot.** | **Was there redness (or shining of the skin) spreading away from the wound?** | **Was the area around the wound warmer than the surrounding skin?** | **Has any part of the wound leaked thin clear fluid?** | **Has any part of the wound leaked blood-stained fluid?** | **Has any part of the wound leaked thick and yellow or green fluid?** | **Have the edges of any part of the wound separated or gaped open of their accord?** | **If the wound edges opened, did the flesh beneath the skin or the inside sutures also separate?** | **Has the area around the wound become swollen?** | **Has the wound been smelly?** | **Has the wound been painful to touch?** | **Have you had, or felt like you have had, a raised temperature or fever?** | |  |

# Table S2. Summary of qualitative data used in adaptation of ‘treatment’ items (12 to 19)

| **Thematic domain** | **Item 12** | **Item 13** | **Item 14** | **Item 15** | **Item 16** | **Item 17** | **Item 18** | **Item 19** |
| --- | --- | --- | --- | --- | --- | --- | --- | --- |
|  | **Have you sought advice because of a problem with your wound, other than at a planned follow-up appointment?** | **Has anything been put on the skin to cover the wound? (dressing)** | **Have you been back into hospital for a problem with your wound?** | **Have you been given antibiotics for a problem with your wound?** | **Have the edges of your wound been deliberately separated by a doctor or nurse?** | **Has your wound been scraped or cut to remove any unwanted tissue?** | **Has your wound been drained? (drainage of pus or an abscess)** | **Have you had an operation under general anaesthetic for treatment of a problem with your wound?** |
| **Ghana** | | | | | | | | |
| Comprehension | Easy to answer for patients. Will be given a routine review date before discharge, a will be told to come back early if needed (Interview GH001I). | Dressing is not a common term to use in Ghana (Interview GH002I). | None | Patients may not know which medications are antibiotics and which are not. May be an assumption for the interviewer based on other answers (Interview GH002I). | Doctor may have removed a stitch to open the wound – patients should find this easy to answer (Interview GH002I). | None | Patients may stay ‘water coming from wound’, but would be able to identify whether this is bloody, or yellow/green colour 'water' (Interviewer GH001I) | Patients will not understand what a 'general anaesthetic' is. Suggest term 'put to sleep for an operation' (Interviewer GH001I). |
| Response mapping | None | None | None | None | None | None | None | None |
| Retrieval | None | None | None | Patients might struggle to remember which medications they were on early after surgery (Focus group 001F). | None | None | None | Patients will definitely remember as this is such a big event. May be difficult to differentiate 'for a wound problem' versus other reason (Interviewer GH002I) |
| Judgment | None | None | Telling the difference between seeking advice related to a wound problem and going back to hospital difficult (Interview GH001I). | Most patients will be discharged on prophylactic antibiotics from hospital. Unsure whether patients will be able to tell the difference between antibiotics for treatment or prevention (Interview GH001I). | May be some confusion around removal of stitches and opening of a wound (Interview GH001I). | None | Might be hard to differentiate between wound being actively drained and passively draining 'water' (Items 3,4,5) (Interviewer GH002I). | Very likely to be able to answer, as a serious event to return to surgery. Burst abdomen able to report easily (Interviewer GH001I). |
| Translatability | None | Patients in Ghana use the term 'washing the wound' to describe wound care and dressing, rather than the term dressing (Interview GH001I). | None | None | None | The word tissue is not widely used in Ghana. Suggest the term 'flesh' or 'skin' e.g. the fleshy part underlying the skin (Interview GH001I). | Abscess is not a word that is commonly used or understood by Ghanian patients (Focus group 001F). | None |
| Other context | It would interesting to explore where the patient had sought advice from, for example from a traditional healer, community healthworker or local health centre (Focus group 001F). | None | Patients will always return to the hospital with any postoperative problems, as traditional healers wont tamper with surgical wounds. Patients will either go back to either district or main hospital (Focus group 001F). | Patients unlikely to be able to read out a medication name over the telephone. Relatives may be able to help answer if more literate (Interview GH001I). | None | None | None | None |
| **Nigeria** | | | | | | | | |
| Comprehension | None | Dressings is a term used by patients (Focus group NG001F). | None | Reasonable to assume they will be able to tell whether an antibiotic prescription is due to wound problems (Interview NG003I). | Can happen both on ward & in-theatre, needs to ensure that this is different to Item 19 (Focus group NG002F). Need to be clearly differentiated from reoperation (e.g. in a minors or majors theatre) versus ward/community based wound opening. For example any point in time when doctors/nurses need to separate wound (Interview NG001I). | None | Suggested that yellow or green pus would improve the description (Focus group NG002F). Abscess is not a terminology well known for patients in Nigeria (Focus group NG001F). | May be difficulty in understanding general anaesthetic (Focus group NG001F). Patients won't understand general anaesthesia, a better terminology would be ‘put to sleep' (Interview NG002I). Difficult to understand 'general anaesthesia' for Nigerian patients. May be difficult to differentiate re-operation for wound and another cause (Focus group NG002F). |
| Response mapping | None | None | None | None | None | None | None | None |
| Retrieval | None | Not many patients would leave hospital with wound dressings still in place, so patients likely to remember if so (Interview NG002I). | Easy for patients to recall as a set event (Interview NG003I). | In general patients will know which drug they are on, and typically will ask what they are for, so likely to be able to answer this (Interview NG001I). | None | None | None | GA will definitely be something a patient remembers (Interview NG001I). |
| Judgment | None | None | None | However patients may get confused by postoperative prophylactic antibiotics which can be for a prolonged period in Nigeria, but specifically for problem with a wound can be clarified to clear their doubts (Interview NG001I). Patients might find it hard to tell the difference between antibiotics and other medications - this may require furrther explanation or the names of the antibiotics (if literate) (Focus group NG002F). | None | Patients will only be able to answer this if the doctor explains it to them at the time of debridement (Interview NG003I). | None | None |
| Translatability | N/A | N/A | N/A | N/A | N/A | N/A | N/A | N/A |
| Other context | 1st point of contact would be known health worker (doctor/nurse). The question would capture community healers. Other than planned follow-up appointment is useful phrasing to support this (Interview NG001I). This would be a clinic, local hospital, main hospital or community health worker at house (80-90%). <10% of patients would go to traditional healer. (Interview NG002I) | Change of dressing wound be performed by community health worker. Patients may use own dressings (non-standardised) which might include this, of varying materials (Interview NG001I). | Patient may return to hospital where it was performed, or may be sent elsewhere if live a long distance away (Focus group NG002F). | None | Deliberately may have bad connotation in Nigeria - this sounds like a malicious act (Interview NG003I). | Often performed during the process of wound dressing (Interview NG001I). | Pus often releases through pressure on edges or when cut to release pus (Interview NG001I). | None |
| **India** | | | | | | | | |
| Comprehension | None | None | None | Patients should know if antibiotic has been provided to them e.g. from pharmacy/doctor (Interview IN002I). | None | None | Patients in general will know the terminology pus but probably not the term 'abscess' (Interview IN002I). | None |
| Response mapping | None | None | None | None | None | None | None | None |
| Retrieval | None | None | None | None | None | None | None | None |
| Judgment | None | None | None | Accuracy may be variable depending on their level of education and the area in which they live (Interview IN001I). | None | None | None | None |
| Translatability | None | None | None | None | None | None | When translating into Hindi, pus translates as 'bad blood' (Focus group IN001F). | None |
| Other context | Some patients with complications would travel back to CMC Vellow, others live farther away and would either attend primary or secondary care depending on the area in which they live (Interview IN001I). | Patients often go home with wound care packages, or visit the hospital or healthcare centre for wound care (Interview IN001I). | None | None | None | None | None | Some patients will have spinal anaesthetic for treatment of problesm with their wounds in an operating room but this would not be captured (Focus group IN001F) |
| **Mexico** | | | | | | | | |
| Comprehension | None | None | None | None | None | None | Pus an acceptable and comprehensible term (Interview MX002I). | Patients unlikely to understand the term general anaesthesia (Foucs group MX001F). |
| Response mapping | None | None | None | None | None | None | None | None |
| Retrieval | None | None | None | Antibiotics are one of the key things they 'like' to receive after an operation (Interview MX002I). | None | None | None | None |
| Judgment | None | None | None | Patients will be very aware of antibiotics they received & timing of administration (Interview MX001I). | None | None | None | None |
| Translatability | None | None | None | None | None | None | Patients won't understand the word abscess in Spanish language - concept translates poorly. | None |
| Other context | Location of help sought will vary depending on the patient's living location; this might be returning to the original hospital or an alternative primary or secondary care location (Interview MX001I) | None | None | None | None | None | None | None |
| **Rwanda** | | | | | | | | |
| Comprehension | None | None | None | Confusion between pain medications and antiobiotics for some patients (interview RW002I). | None | None | Concept of pus well understood, and patients can differentiate abnormal fluid. Well differentiated from spontaneous drainage of thick green fluid in item 5 (Interview RW001I). | Second operation' helps with clarification (Interview RW002I). Patients understand the concept of general anaesthesia (Interview RW001). |
| Response mapping | None | None | None | None | None | None | None | None |
| Retrieval | None | None | None | None | None | None | None | None |
| Judgment | None | None | None | When clarifying the type of medication, the form of delivery is a useful addition (e.g., capsule versus tablets). Abx usually take the form of capsules (Focus group RW001). | None | None | None | None |
| Translatability | None | None | None | None | None | None | Pus translates well into Kinyarwanda (Focus group RW001F). | None |
| Other context | None | None | None | None | None | This often occurs in a minor operating room or clinical room in Rwanda (Focus group RW001F). | None | None |
| **Benin** | | | | | | | | |
| Comprehension | None | None | None | None | Deliberately is easy to understand in French and would not hold negative connotations in Benin (Interview BN001I). | None | None | None |
| Response mapping | None | None | None | None | None | None | None | None |
| Retrieval | None | None | None | None | None | None | None | None |
| Judgment | None | None | None | None | None | None | None | None |
| Translatability | None | None | None | None | None | None | None | None |
| Other context | None | None | None | None | None | None | None | None |
| **Adapted Wound Healing Questionnaire** | **Have you sought advice because of a problem with your wound, other than at a planned follow-up appointment?** | **Has anything been put on the skin to cover the wound? (dressing)** | **Have you been back into hospital for a problem with your wound?** | **Have you been given medicines (antibiotics) for a problem with your wound?** | **Have the edges of your wound been separated by a doctor or nurse?** | **Has your wound been scraped or cut to remove any unwanted flesh?** | **Has thick, yellow or green fluid (pus) been drained from your wound by a doctor or nurse (abscess)?** | **Have you had to go back to the operating room for treatment of a problem with your wound?** |
| **Summary of recommendations for adaptation and implementation** | No changes | No changes | No changes | Change to medicines (antibiotics) in line with other UROM-style items.  Assessors recommended to ask patients to read out their medications if possible to double check that they are antibiotics. | Clarification that this question is specifically examining separation of the wound edges, not under general anaesthesia. Word 'deliberately' removed due to perceived negative connotations. | Change to the word tissue to 'flesh' as consensus that this was medical terminology | Limited understanding of the terminology abscess. Concern about crossover with fluid drainage (yellow/green) in Item 5 - decision from working group to highlight drainage 'by doctor or nurse' as an active event. | Consensus that general anaesthesia will not be a concept well understood by LMIC populations. Theme that spinal anaesthesia very common in some LMICs, so would not capture these operations. Terminology adapted to collect information about any procedure carried out in the OR. |

# Table S3. Class interval structure in Rasch analysis

|  |  | **Class interval** | | | | |
| --- | --- | --- | --- | --- | --- | --- |
| **Item** | **Description** | **1** | **2** | **3** | **4** | **5** |
| **I0001** | Redness | 110 | 96 | 75 | 74 | 14 |
| **I0002** | Heat | 110 | 96 | 75 | 74 | 14 |
| **I0003** | Clear fluid | 110 | 96 | 75 | 74 | 14 |
| **I0004** | Blood-stained fluid | 110 | 96 | 75 | 74 | 14 |
| **I0005** | Purulent fluid | 110 | 96 | 75 | 74 | 14 |
| **I0006** | Wound opening | 110 | 96 | 74 | 74 | 14 |
| **I0007** | Deep wound opening | 10 | 11 | 10 | 9 | 6 |
| **I0008** | Local swelling | 110 | 96 | 75 | 74 | 14 |
| **I0009** | Smell | 110 | 96 | 75 | 75 | 12 |
| **I0010** | Tenderness | 110 | 96 | 75 | 74 | 14 |
| **I0011** | Fever | 110 | 96 | 74 | 74 | 14 |
| **I0012** | Advice | 110 | 96 | 75 | 74 | 14 |
| **I0013** | Dressing | 110 | 96 | 75 | 74 | 14 |
| **I0014** | Readmission | 110 | 96 | 75 | 74 | 14 |
| **I0015** | Antibiotics | 110 | 96 | 75 | 74 | 14 |
| **I0016** | Deliberate opening | 110 | 96 | 75 | 74 | 14 |
| **I0017** | Wound scraping | 110 | 96 | 75 | 74 | 14 |
| **I0018** | Wound drained | 110 | 96 | 75 | 74 | 14 |
| **I0019** | Reoperated | 110 | 96 | 75 | 74 | 14 |

# Table S4. Individual item fit in Rasch analysis

| Item | Description | Location | SE | Fit residual | DF | χ^2^ | DF | Prob | F-statistic | DF1 | DF2 | Prob |
| --- | --- | --- | --- | --- | --- | --- | --- | --- | --- | --- | --- | --- |
| I0001 | Redness | -0.54 | 0.106 | -0.459 | 346.42 | 6.129 | 4 | 0.189686 | 1.28 | 4 | 364 | 0.277285 |
| I0002 | Heat | 0.148 | 0.123 | -0.574 | 346.42 | 5.142 | 4 | 0.27303 | 0.965 | 4 | 364 | 0.426828 |
| I0003 | Clear fluid | -0.012 | 0.125 | -0.402 | 346.42 | 2.158 | 4 | 0.706659 | 0.387 | 4 | 364 | 0.817702 |
| I0004 | Blood-stained fluid | 2.358 | 0.156 | -1.863 | 346.42 | 5.396 | 4 | 0.249023 | 1.461 | 4 | 364 | 0.213624 |
| ***I0005*** | ***Purulent fluid*** | -0.063 | 0.126 | -2.837 | 346.42 | 11.977 | 4 | 0.017525 | 4.809 | 4 | 364 | 0.000864 |
| I0006 | Wound opening | 0.166 | 0.137 | -1.769 | 345.49 | 7.386 | 4 | 0.116833 | 1.513 | 4 | 363 | 0.197776 |
| I0007 | Deep wound opening | 0.294 | 0.243 | 0.407 | 43.19 | 5.71 | 4 | 0.221911 | 1.232 | 4 | 41 | 0.31248 |
| I0008 | Local swelling | 2.196 | 0.134 | -1.251 | 346.42 | 7.844 | 4 | 0.097462 | 1.905 | 4 | 364 | 0.108954 |
| ***I0009*** | ***Smell*** | 0.115 | 0.137 | -3.519 | 345.49 | 14.846 | 4 | 0.005032 | 6.62 | 4 | 363 | 0.000044 |
| ***I0010*** | ***Tenderness*** | -1.332 | 0.083 | 2.535 | 346.42 | 31.471 | 4 | 0.000002 | 7.273 | 4 | 364 | 0.000015 |
| ***I0011*** | ***Fever*** | -0.503 | 0.107 | 0.172 | 345.49 | 24.686 | 4 | 0.000059 | 6.607 | 4 | 363 | 0.000041 |
| ***I0012*** | ***Advice*** | -0.979 | 0.153 | -2.772 | 346.42 | 11.437 | 4 | 0.022073 | 3.403 | 4 | 364 | 0.009473 |
| I0013 | Dressing | -3.992 | 0.122 | 0.028 | 346.42 | 5.976 | 4 | 0.200922 | 1.691 | 4 | 364 | 0.151444 |
| ***I0014*** | ***Readmission*** | -0.433 | 0.178 | -4.5 | 346.42 | 18.184 | 4 | 0.001137 | 10.985 | 4 | 364 | 0 |
| ***I0015*** | ***Antibiotics*** | -1.617 | 0.134 | -3.891 | 346.42 | 25.537 | 4 | 0.000041 | 8.655 | 4 | 364 | 0 |
| ***I0016*** | ***Clinician opening*** | 0.847 | 0.277 | -3.213 | 346.42 | 9.51 | 4 | 0.04954 | 8.658 | 4 | 364 | 0.000002 |
| I0017 | Wound scraping | 1.489 | 0.361 | -2.335 | 346.42 | 4.568 | 4 | 0.334617 | 3.285 | 4 | 364 | 0.011551 |
| I0018 | Wound drained | 0.467 | 0.239 | -1.883 | 346.42 | 4.61 | 4 | 0.329672 | 1.781 | 4 | 364 | 0.132017 |
| ***I0019*** | ***Reoperated*** | 1.392 | 0.347 | -2.497 | 346.42 | 6.673 | 4 | 0.154187 | 6.222 | 4 | 364 | 0.000076 |

Items that appeared to misfit the Rasch model based on one or more assessment criteria highlighted in bold. P-values highlighted for Bonferroni-adjusted value of P<0.000877 (base alpha 0.05) to account for multiplicity in testing.

# Table S5. Exploration of item correlations and local dependency between items in Rasch analysis

| **Item** | **I0001** | **I0002** | **I0003** | **I0004** | **I0005** | **I0006** | **I0007** | **I0008** | **I0009** | **I0010** | **I0011** | **I0012** | **I0013** | **I0014** | **I0015** | **I0016** | **I0017** | **I0018** | **I0019** |
| --- | --- | --- | --- | --- | --- | --- | --- | --- | --- | --- | --- | --- | --- | --- | --- | --- | --- | --- | --- |
| **I0001** |  |  |  |  |  |  |  |  |  |  |  |  |  |  |  |  |  |  |  |
| **I0002** | 0.032 |  |  |  |  |  |  |  |  |  |  |  |  |  |  |  |  |  |  |
| **I0003** | -0.128 | -0.082 |  |  |  |  |  |  |  |  |  |  |  |  |  |  |  |  |  |
| **I0004** | -0.042 | -0.061 | 0.041 |  |  |  |  |  |  |  |  |  |  |  |  |  |  |  |  |
| **I0005** | -0.131 | -0.097 | 0.028 | -0.067 |  |  |  |  |  |  |  |  |  |  |  |  |  |  |  |
| **I0006** | -0.014 | -0.094 | 0.021 | 0.071 | 0.016 |  |  |  |  |  |  |  |  |  |  |  |  |  |  |
| **I0007** | -0.159 | -0.217 | -0.062 | 0.182 | -0.305 | 0.333 |  |  |  |  |  |  |  |  |  |  |  |  |  |
| **I0008** | -0.021 | 0.049 | -0.054 | -0.079 | -0.12 | -0.103 | 0.098 |  |  |  |  |  |  |  |  |  |  |  |  |
| **I0009** | -0.074 | -0.1 | -0.121 | 0.107 | 0.085 | -0.019 | -0.258 | -0.017 |  |  |  |  |  |  |  |  |  |  |  |
| **I0010** | -0.209 | -0.065 | -0.104 | -0.08 | -0.118 | -0.163 | -0.158 | -0.036 | -0.129 |  |  |  |  |  |  |  |  |  |  |
| **I0011** | -0.098 | -0.065 | -0.084 | -0.128 | -0.049 | -0.075 | -0.023 | -0.174 | -0.071 | -0.057 |  |  |  |  |  |  |  |  |  |
| **I0012** | -0.139 | -0.056 | -0.019 | -0.087 | 0.043 | -0.051 | -0.3 | -0.104 | -0.085 | -0.145 | -0.029 |  |  |  |  |  |  |  |  |
| **I0013** | 0.054 | -0.192 | -0.174 | -0.121 | -0.036 | -0.115 | -0.073 | -0.088 | 0.077 | -0.442 | -0.069 | 0.023 |  |  |  |  |  |  |  |
| **I0014** | -0.108 | -0.126 | -0.09 | -0.028 | 0.037 | 0.004 | 0.174 | -0.003 | -0.041 | -0.147 | -0.171 | 0.178 | 0.088 |  |  |  |  |  |  |
| **I0015** | -0.174 | -0.085 | 0.055 | -0.071 | 0.101 | -0.076 | -0.036 | -0.116 | -0.012 | -0.216 | -0.092 | 0.106 | -0.01 | 0.209 |  |  |  |  |  |
| **I0016** | -0.06 | -0.102 | -0.094 | -0.005 | -0.016 | 0.053 | 0.037 | -0.041 | 0.162 | -0.164 | -0.18 | 0.039 | 0.034 | 0.167 | 0.072 |  |  |  |  |
| **I0017** | 0.009 | -0.087 | -0.108 | -0.007 | -0.031 | 0.074 | -0.009 | -0.059 | 0.111 | -0.101 | -0.119 | -0.02 | 0.024 | 0.155 | -0.003 | 0.413 |  |  |  |
| **I0018** | -0.08 | -0.04 | 0.099 | 0.086 | -0.024 | -0.037 | -0.223 | -0.073 | -0.041 | -0.163 | -0.128 | 0.119 | 0.017 | 0.032 | 0.143 | 0.212 | 0.015 |  |  |
| **I0019** | -0.123 | -0.098 | -0.077 | -0.016 | -0.028 | 0.051 | 0.403 | -0.018 | 0.01 | -0.1 | -0.149 | -0.015 | 0.025 | 0.248 | 0.064 | 0.67 | 0.409 | 0.155 |  |

Highlighted residual correlation coefficients are +0.2 above the mean average of all item residual correlations (-0.027) (58)

# Table S6. Exploration of differential item functioning by country

|  |  | **Class interval (ANOVA)** | | | | | **Country  (uniform DIF)** | | | | **Class interval by country  (non-uniform DIF)** | | | |
| --- | --- | --- | --- | --- | --- | --- | --- | --- | --- | --- | --- | --- | --- | --- |
| **Item** | **Description** | **MS** | **F-statistic** | **DF** | **Prob** | **MS** | | **F-statistic** | **DF** | **Prob** | **MS** | **F-statistic** | **DF** | **Prob** |
| **I0001** | Redness | 1.10905 | 1.44985 | 4 | 0.217174 | 5.95565 | | 7.78575 | 4 | 0.000001 | 1.8406 | 2.40619 | 15 | 0.002484 |
| **I0002** | Heat | 0.81154 | 0.97723 | 4 | 0.419959 | 1.32731 | | 1.59832 | 4 | 0.17429 | 0.95976 | 1.15572 | 15 | 0.305279 |
| **I0003** | Clear fluid | 0.33813 | 0.40548 | 4 | 0.804693 | 5.13824 | | 6.1617 | 4 | 0.000076 | 0.63418 | 0.7605 | 15 | 0.721338 |
| **I0004** | Blood-stained fluid | 0.82023 | 1.58117 | 4 | 0.178817 | 0.58955 | | 1.13649 | 4 | 0.339085 | 1.53853 | 2.96583 | 15 | 0.000184 |
| **I0005** | Purulent fluid | 2.03576 | 5.2885 | 4 | 0.000383 | 2.55904 | | 6.64789 | 4 | 0.00004 | 0.736 | 1.91197 | 15 | 0.021178 |
| **I0006** | Wound opening | 0.97441 | 1.57546 | 4 | 0.180351 | 2.25915 | | 3.65269 | 4 | 0.006259 | 0.79936 | 1.29243 | 15 | 0.204236 |
| **I0007** | Deep wound opening | 1.42662 | 1.7364 | 4 | 0.169002 | 2.40595 | | 2.9284 | 3 | 0.050296 | 1.82772 | 2.2246 | 9 | 0.049837 |
| **I0008** | Local swelling | 1.47699 | 2.13467 | 4 | 0.076134 | 5.33207 | | 7.70634 | 4 | 0.000005 | 1.47582 | 2.13297 | 15 | 0.008346 |
| **I0009** | Smell | 2.47752 | 6.98484 | 4 | 0.000021 | 1.35706 | | 3.82596 | 4 | 0.004665 | 0.56007 | 1.57901 | 15 | 0.077303 |
| **I0010** | Tenderness | 7.85005 | 8.81738 | 4 | 0 | 12.63569 | | 14.19274 | 4 | 0.000006 | 2.34485 | 2.63379 | 15 | 0.000872 |
| **I0011** | Fever | 6.05665 | 6.72738 | 4 | 0.000032 | 2.17053 | | 2.4109 | 4 | 0.04896 | 0.95995 | 1.06626 | 15 | 0.386969 |
| **I0012** | Advice | 1.95626 | 3.47161 | 4 | 0.008488 | 0.55603 | | 0.98674 | 4 | 0.414748 | 0.83991 | 1.49052 | 15 | 0.106046 |
| **I0013** | Dressing | 1.58484 | 2.09556 | 4 | 0.080998 | 11.54038 | | 15.2593 | 4 | 0 | 2.27201 | 3.00417 | 15 | 0.000153 |
| **I0014** | Readmission | 3.30776 | 10.88698 | 4 | 0 | 0.18136 | | 0.59693 | 4 | 0.665092 | 0.27042 | 0.89004 | 15 | 0.575761 |
| **I0015** | Antibiotics | 4.74728 | 8.73443 | 4 | 0.000006 | 1.06867 | | 1.96623 | 4 | 0.099221 | 0.52497 | 0.96588 | 15 | 0.491144 |
| **I0016** | Clinician opening | 1.7993 | 9.72982 | 4 | 0.000005 | 0.59012 | | 3.19112 | 4 | 0.013583 | 0.63243 | 3.4199 | 15 | 0.000022 |
| **I0017** | Wound scraping | 0.7204 | 3.65057 | 4 | 0.006284 | 0.68523 | | 3.47234 | 4 | 0.008476 | 0.6001 | 3.04095 | 15 | 0.000128 |
| **I0018** | Wound drained | 0.83979 | 1.85386 | 4 | 0.118133 | 0.73257 | | 1.61718 | 4 | 0.16943 | 0.82874 | 1.82947 | 15 | 0.029577 |
| **I0019** | Reoperated | 1.28757 | 7.12483 | 4 | 0.000023 | 0.36665 | | 2.02889 | 4 | 0.089948 | 0.76782 | 4.24876 | 15 | 0 |

# Table S7. Exploration of differential item functioning by patient home location (urban versus rural)

|  |  | **Class interval (ANOVA)** | | | | | **Home location  (uniform DIF)** | | | | **Class interval by home location  (non-uniform DIF)** | | | |
| --- | --- | --- | --- | --- | --- | --- | --- | --- | --- | --- | --- | --- | --- | --- |
| **Item** | **Description** | **MS** | **F-statistic** | **DF** | **Prob** | **MS** | | **F-statistic** | **DF** | **Prob** | **MS** | **F-statistic** | **DF** | **Prob** |
| **I0001** | Redness | 1.10906 | 1.28119 | 4 | 0.276925 | 1.97513 | | 2.28168 | 1 | 0.131792 | 0.64867 | 0.74935 | 4 | 0.558954 |
| **I0002** | Heat | 0.81154 | 0.96149 | 4 | 0.428636 | 0.05993 | | 0.07101 | 1 | 0.790022 | 0.78441 | 0.92934 | 4 | 0.446887 |
| **I0003** | Clear fluid | 0.33813 | 0.38624 | 4 | 0.818478 | 0.00328 | | 0.00375 | 1 | 0.951239 | 0.86892 | 0.99256 | 4 | 0.411526 |
| **I0004** | Blood-stained fluid | 0.82023 | 1.45809 | 4 | 0.214462 | 0.55646 | | 0.9892 | 1 | 0.320601 | 0.47444 | 0.8434 | 4 | 0.498362 |
| **I0005** | Purulent fluid | 2.03576 | 4.8977 | 4 | 0.000742 | 0.30434 | | 0.73218 | 1 | 0.392754 | 1.13895 | 2.74012 | 4 | 0.028591 |
| **I0006** | Wound opening | 0.97441 | 1.51754 | 4 | 0.196467 | 0.61448 | | 0.95699 | 1 | 0.328607 | 0.82575 | 1.28603 | 4 | 0.275033 |
| **I0007** | Deep wound opening | 1.42662 | 1.30231 | 4 | 0.28761 | 2.07281 | | 1.8922 | 1 | 0.177453 | 1.49612 | 1.36576 | 4 | 0.265085 |
| **I0008** | Local swelling | 1.47699 | 1.92532 | 4 | 0.105648 | 1.86336 | | 2.42897 | 1 | 0.119991 | 1.22648 | 1.59877 | 4 | 0.174069 |
| **I0009** | Smell | 2.47751 | 6.56173 | 4 | 0.000034 | 0.04832 | | 0.12797 | 1 | 0.720756 | 0.15683 | 0.41536 | 4 | 0.797571 |
| **I0010** | Tenderness | 7.85005 | 7.53315 | 4 | 0.000005 | 6.50787 | | 6.24515 | 1 | 0.012894 | 3.064 | 2.94031 | 4 | 0.02055 |
| **I0011** | Fever | 6.05665 | 6.56487 | 4 | 0.000045 | 0.19971 | | 0.21647 | 1 | 0.642034 | 0.5747 | 0.62292 | 4 | 0.646432 |
| **I0012** | Advice | 1.95626 | 3.38917 | 4 | 0.009717 | 0.05805 | | 0.10057 | 1 | 0.751322 | 0.48865 | 0.84657 | 4 | 0.496397 |
| **I0013** | Dressing | 1.58484 | 1.70809 | 4 | 0.147585 | 0.66633 | | 0.71815 | 1 | 0.397315 | 1.84948 | 1.99331 | 4 | 0.095016 |
| **I0014** | Readmission | 3.30776 | 11.28915 | 4 | 0.000008 | 0.00005 | | 0.00016 | 1 | 0.989568 | 1.10343 | 3.76593 | 4 | 0.005144 |
| **I0015** | Antibiotics | 4.74728 | 9.06901 | 4 | 0.000008 | 1.85356 | | 3.54097 | 1 | 0.060681 | 2.47129 | 4.72105 | 4 | 0.001009 |
| **I0016** | Clinician opening | 1.7993 | 8.64645 | 4 | 0.000006 | 0.21679 | | 1.04179 | 1 | 0.308094 | 0.18073 | 0.86851 | 4 | 0.482919 |
| **I0017** | Wound scraping | 0.7204 | 3.30981 | 4 | 0.011091 | 0.35397 | | 1.62629 | 1 | 0.20304 | 0.33296 | 1.52974 | 4 | 0.192945 |
| **I0018** | Wound drained | 0.83979 | 1.78495 | 4 | 0.131242 | 0.02946 | | 0.06261 | 1 | 0.802543 | 0.678 | 1.44107 | 4 | 0.219887 |
| **I0019** | Reoperated | 1.28757 | 6.1947 | 4 | 0.000072 | 0.18986 | | 0.91344 | 1 | 0.339846 | 0.13069 | 0.62876 | 4 | 0.642267 |

# Figure S1. Differential item functioning by country for symptoms items 1 (Redness), 8 (Local swelling), 10 (Tenderness), 11 (Fever)

**
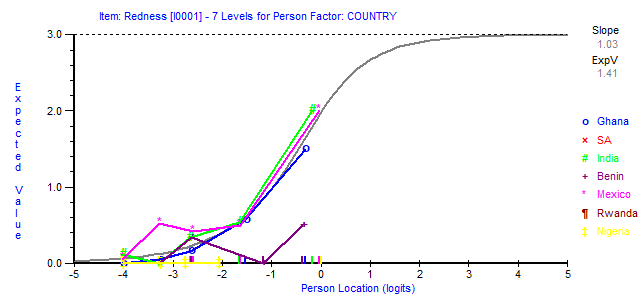
**

**
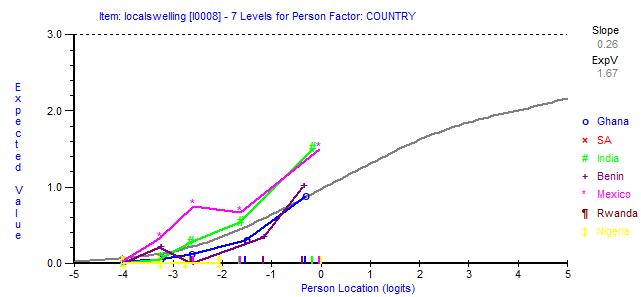
**

**
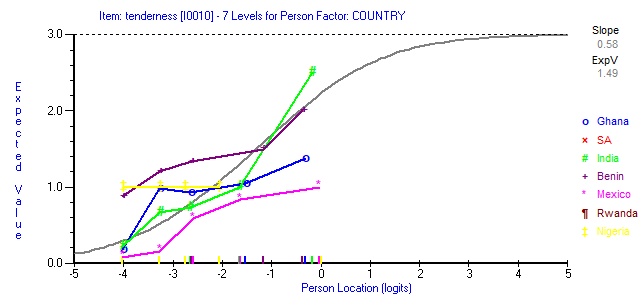
**

**
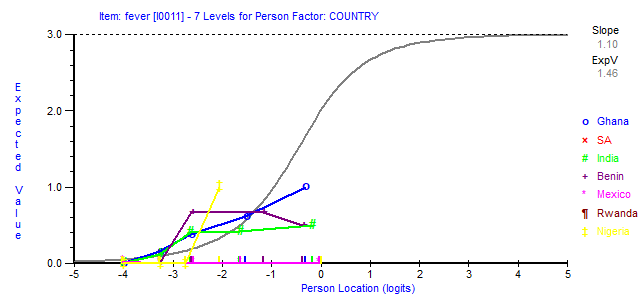
**

# Figure S2. Threshold probability map after subtest analysis of item 3 (clear fluid), item 4 (blood-stained fluid) and item 5 (purulent fluid)

**
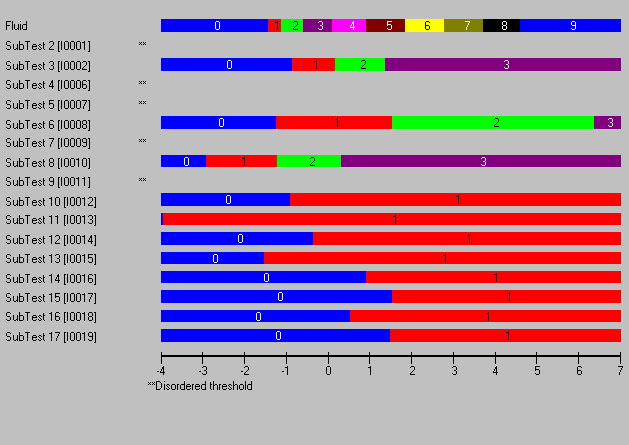
**

# Figure S3. Category probability curve upon subtest analysis of item 6 (wound opening) and item 7 (deep wound opening)

**
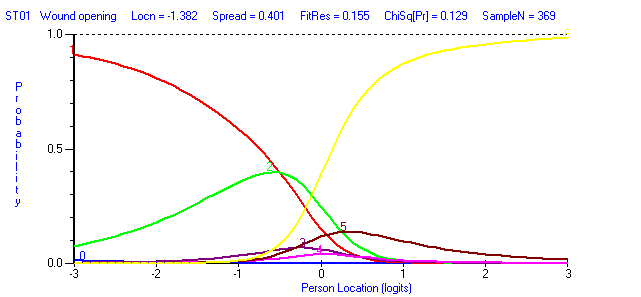
**

# Figure S4. Differential item functioning by country for pathway items 14 (Readmission), 15 (Antibiotics), 16 (Clinician opening), 19 (Reoperated)

**
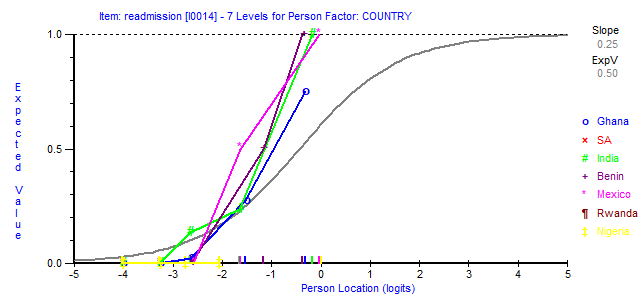

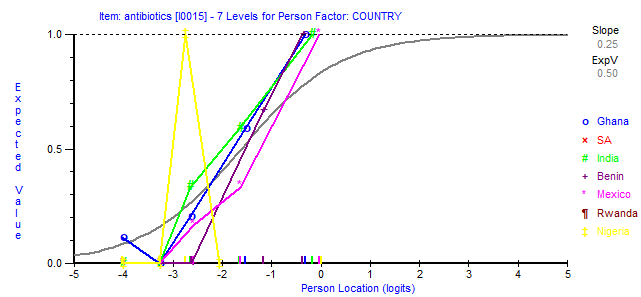
**

**
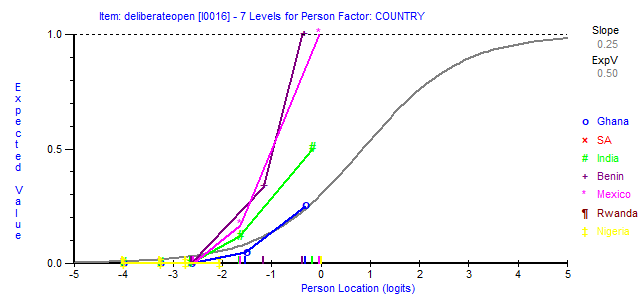
**

**
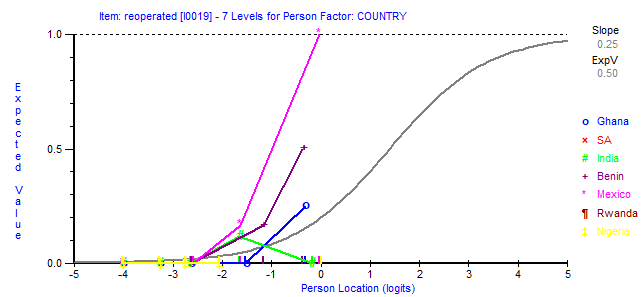
**

# References

1. Baena I, Padilla J. Cognitive interviewing in mixed research. Cognitive interviewing methodology. London: John Wiley & Sons; 2014. p. 133-52.

2. Wild D, Grove A, Martin M, Eremenco S, McElroy S, Verjee-Lorenz A, et al. Principles of Good Practice for the Translation and Cultural Adaptation Process for Patient-Reported Outcomes (PRO) Measures: report of the ISPOR Task Force for Translation and Cultural Adaptation. Value Health. 2005;8(2):94-104.

3. Death following pulmonary complications of surgery before and during the SARS-CoV-2 pandemic. Br J Surg. 2021;108(12):1448-64.

4. Mortality and pulmonary complications in patients undergoing surgery with perioperative SARS-CoV-2 infection: an international cohort study. Lancet. 2020;396(10243):27-38.

5. Macefield RC, Reeves BC, Milne TK, Nicholson A, Blencowe NS, Calvert M, et al. Development of a single, practical measure of surgical site infection (SSI) for patient report or observer completion. J Infect Prev. 2017;18(4):170-9.

6. Bluebelle-Study-Group. Validation of the Bluebelle Wound Healing Questionnaire for assessment of surgical-site infection in closed primary wounds after hospital discharge. Br J Surg. 2019;106(3):226-35.

7. Elliott D, Bluebelle-Study-Group. Developing outcome measures assessing wound management and patient experience: a mixed methods study. BMJ Open. 2017;7(11):e016155.

8. Ponterotto J. Brief Note on the Origins, Evolution, and Meaning of the Qualitative Research Concept Thick Description. The Qualitative Report. 2006;11.

9. Willis G. Cognitive Interviewing, Chapter 7: Analyzing and Documenting Cognitive Interview Results. SAGE Publications, Inc2011.

10. Tembo D, Hickey G, Montenegro C, Chandler D, Nelson E, Porter K, et al. Effective engagement and involvement with community stakeholders in the co-production of global health research. Bmj. 2021;372:n178.

11. Tennant A, Penta M, Tesio L, Grimby G, Thonnard JL, Slade A, et al. Assessing and adjusting for cross-cultural validity of impairment and activity limitation scales through differential item functioning within the framework of the Rasch model: the PRO-ESOR project. Med Care. 2004;42(1 Suppl):I37-48.

12. Tesio L, Scarano S, Hassan S, Kumbhare D, Caronni A. WHY QUESTIONNAIRE SCORES ARE NOT MEASURES: A QUESTION-RAISING ARTICLE. Am J Phys Med Rehabil. 2022.

13. Hughes SE, Haroon S, Subramanian A, McMullan C, Aiyegbusi OL, Turner GM, et al. Development and validation of the symptom burden questionnaire for long covid (SBQ-LC): Rasch analysis. Bmj. 2022;377:e070230.

14. Andrich D. Rating scales and Rasch measurement. Expert Rev Pharmacoecon Outcomes Res. 2011;11(5):571-85.
